# Genome analysis and virulence gene expression profile of a multi drug resistant *Salmonella enterica* serovar Typhimurium ms202

**DOI:** 10.1186/s13099-022-00498-w

**Published:** 2022-06-28

**Authors:** Nirmal Kumar Mohakud, Rakesh Kumar Panda, Saumya Darshana Patra, Bikash Ranjan Sahu, Mrinmoy Ghosh, Gajraj Singh Kushwaha, Namrata Misra, Mrutyunjay Suar

**Affiliations:** 1 School of Biotechnology, KIIT University, Bhubaneswar, 751024 India; 2 Kalinga Institute of Medical Sciences (KIMS), KIIT University, Bhubaneswar, 751024 India; 3 SCB Medical College, Cuttack, India; 4 KIIT-Technology Business Incubator (KIIT-TBI), KIIT University, Bhubaneswar, 751024 India

**Keywords:** Whole genome sequence, *Salmonella* Typhimurium, ms202, Virulence

## Abstract

**Background:**

In India, multi-drug resistance in *Salmonella enterica* serovar Typhimurium poses a significant health threat. Indeed, *S.* Typhimurium has remained unknown for a large portion of its genome associated with various physiological functions including mechanism of drug resistance and virulence. The whole-genome sequence of a Salmonella strain obtained from feces of a patient with gastroenteritis in Odisha, India, was analyzed for understanding the disease association and underlying virulence mechanisms.

**Results:**

The de novo assembly yielded 17 contigs and showed 99.9% similarity to *S. enterica* sub sp *enterica* strain LT2 and *S. enteric* subsp *salamae* strain DSM 9220. *S.* Typhimurium ms202 strain constitutes six known *Salmonella* pathogenicity islands and nine different phages. The comparative interpretation of pathogenic islands displayed the genes contained in SPI-1 and SPI-2 to be highly conserved. We identified *sit ABCD* cluster regulatory cascade in SPI-1. Multiple antimicrobial resistance genes were identified that directly implies antibiotic-resistant phenotype. Notably, seven unique genes were identified as "acquired antibiotic resistance". These data suggest that virulence in *S. enterica* Typhimurium ms202 is associated with SPI-1 and SPI-2. Further, we found several virulent genes encoding SPI regions belonging to type III secretion systems (T3SS) of bacteria were significantly upregulated in ms202 compared to control LT2. Moreover, all these genes were significantly downregulated in *S. enterica* Typhimurium ms202 as compared to control LT2 on adding Mn^2+^ exogenously.

**Conclusions:**

Our study raises a vital concern about the potential diffusion of a novel multi-drug resistant *S. enterica* Typhimurium ms202. It justifies this clinical pathogen to demonstrate a higher degree survival due to higher expression of virulent genes and enhanced ability of metallic ion acquisition.

**Supplementary Information:**

The online version contains supplementary material available at 10.1186/s13099-022-00498-w.

## Background

Salmonellosis, one of the primary causes of food-borne infections resulting from gram-negative enteropathogenic bacteria *Salmonella spp*, is a global threat to human health [1]. Besides typhoidal *Salmonella* that causes enteric fever in humans, the non-typhoidal *Salmonella* (NTS) results in acute/chronic gastroenteritis. Annually, the NTS epidemic reports ~ 93.8 million infection cases and ~ 155,000 deaths [2]. Indeed, NTS infections not only typically cause diarrhoea but also result in non-specific febrile illness that is clinically indistinguishable from other febrile illnesses [3].

*Salmonella enterica* subspecies *enterica* comprises more than 2600 serovars [4, 5] with unique somatic (O) and flagellar (H) antigenic formulae used to identify the serovar types [6]. ~ 53 serovars of Salmonella have been identified from India, and the numbers are still counting [7, 8]. Remarkably, among different serovars, *Salmonella enterica* Typhimurium (henceforth *S*. Typhimurium) and *Salmonella enterica* Enteritidis are lead pathogens responsible for causing gastroenteritis in humans [9, 10] typically involved in self-limiting diarrheal illness. Globally as well as in India, invasive diseases cause a high degree of mortality and morbidity. The typhoidal serovars cause invasive disease and are humanly restricted. However, *S.* Typhimurium, also associated with gastroenteritis in cattle, chicken and a wide range of hosts, is considered a relevant zoonotic pathogen [11]. Notably, the epidemiology of *S*. Typhimurium has remained unknown for a large portion of the genome associated with various physiological functions of the bacteria. Indeed, the systemic disease association and mechanism of transmission of virulent factors to other hosts have not been completely understood [12].

The Pathogenicity islands (PAIs) and Resistance islands (REIs) are key regulatory regions that play complementary roles in bacterial pathogenesis. The PAIs, including large gene clusters [10 to 100 kb], play a vital role in mediating pathogenicity through invasion into epithelial cells followed by replication and survival inside phagocytic cells [13, 14]. Interestingly, due to Direct Repeated Sequences, few PAIs can excise from and re-integrate into the bacterial chromosome. Indeed, *Salmonella* Pathogenicity Islands (SPIs) belonging to T3SS play a crucial role in host-cell interaction. Therefore, it is essential to gain detailed knowledge about serotype distribution and the SPI pattern to formulate appropriate therapeutic and intervention strategies against Salmonella induced diseases.

In the current study, we sequenced and annotated the whole genome of a multi-drug-resistant (MDR) *Salmonella enterica* subsp. enterica serovar Typhimurium ms202 (henceforth termed as *S. enterica* Typhimurium ms202) isolated from feces of a patient with gastroenteritis admitted to a hospital in Odisha, India. The in-depth genomic analysis of MDR *S.* Typhimurium and annotation elucidated the genomic features for the distribution of SPI regions, virulence factors and identified candidate genes for antimicrobial resistance. We found that several virulent genes encoding the T3SS were significantly upregulated in *S. enterica* Typhimurium ms202 as compared to control strain, suggesting a higher degree of virulence and fitness of this drug-resistant bacteria. We hypothesize that, due to greater degree of virulence, these bacteria probably utilize metallic ions more efficiently, as shown by a diminished expression of Mn^2+^ transporter genes in *S. enterica* Typhimurium ms202 with respect to control in the presence of Mn^2+^. The genome sequence data provides us a better insight into the mechanisms by which species or serovars have evolved and helps to know the status of antibiotic resistance patterns. The genetic divergence in *S.* Typhimurium reared under diverse geographical locations may help understand the genomic architecture of this newly isolated species.

## Results

### Identification of multi-drug resistant *S. enterica* Typhimurium ms202

The demographic details of 221 patients are shown in Additional file 1: Table S1. As shown in the workflow of 'methods' section, a total of 221 fecal samples was examined by microscopic analysis and biochemical assays to detect *S.* Typhimurium. The microscopic analysis revealed presence of nematode and cestodes in stool of 35 cases (15.8%) indicating presence of parasitic diarrhoea. Major parasites observed through microscopy included Strongyloides, Entamoeba, Giardia, Ascaris, Ankylostoma, Trichuris, Enterobius and Taenia. Biochemical analysis suggested that, nine fecal samples containing bacterial isolates showed an adverse urea reaction along with a positive Triple Sugar Iron (TSI) and citrate reaction identifying the isolates as *Salmonella*. To confirm the genus and species, all nine isolates were examined using VTEK 2 ID card, out of which 6 of them were identified as *Salmonella enterica.* It must be noted that, the remaining three bacterial isolates could not be recognized for any species, possibly due to sample handling error. Further, serotype analysis revealed four out of six strains as *Salmonella enterica* serovar Typhimurium. (The remaining two strains were identified as *S. enterica* Weltevreden). All four isolates of *S. enterica* serovar Typhimurium were subjected to antibiotic susceptibility test to examine drug resistance. The results showed three out of four to be resistant to more than three antibiotic groups considering these pathogens as multidrug-resistant. Of three MDR strains, one isolate (*S. enterica* Typhimurium ms202 in this manuscript) showing resistance to four antibiotic groups was selected for genome analysis study. The resistance pattern of the strain to antibiotics showed in Additional file 2: Table S2. Different antibiotics and antibiotic groups (in brackets) to which the strain was found resistant are as follows: Cefuroxime (Cephalosporin 2nd generation), Nalidixic acid and Ciprofloxacin (Quinolones), Amikacin and Gentamycin (Aminoglycosides) and Tigecycline (Tetracyclines).

### Genome drafting of *S. enterica* Typhimurium ms202

#### Whole genome assembly and assessment

High-throughput sequencing generated a total of 4,894,062 bp with 318-depth coverage determined by accomplishing the Gap closure in the *S. enterica* Typhimurium ms202 entire genome sequence. The details on assembly are shown in Table-1. SOAPdenovo2 software suggested the most appropriate assembler in terms of genome completeness [15]. QC plots, GC content versus contig coverage showed a single cluster, indicating the absence of contamination in data. The total raw reads of 11,410,205 and the HQ read of 5,969,942 were recorded. All contigs were mapped to the reference genome successfully with slight variations in the alignments. An assembled genome was submitted to the Comprehensive Genome Analysis service.

#### Completeness of the genome annotation

**Fig. 1:**
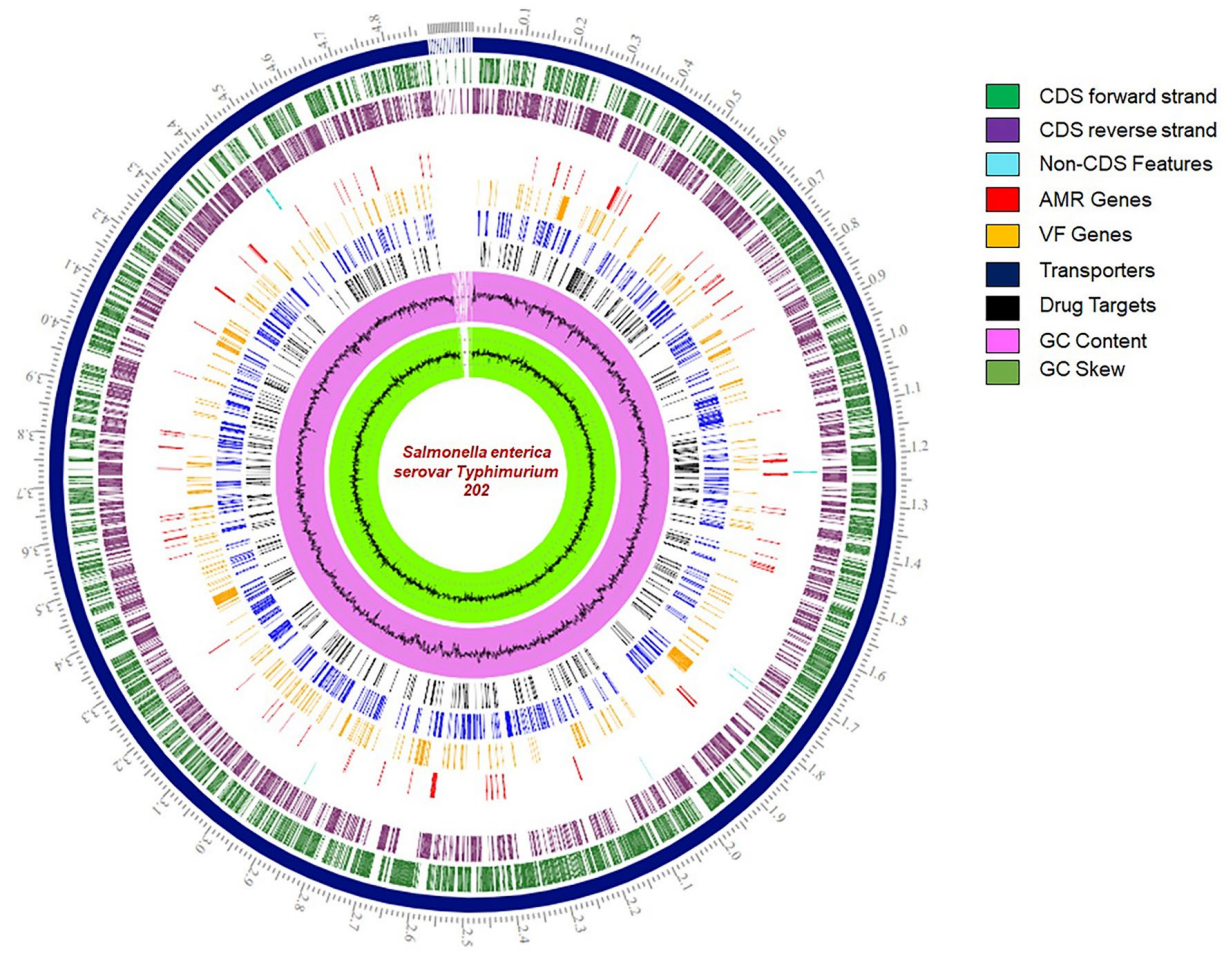
A circular graphical illustration depicting the genome annotations distribution. It includes, from outer to inner rings, the contigs, CDS on the forward strand, CDS on the reverse strand, RNA genes, CDS with homology to known antimicrobial resistance genes, CDS with homology to known virulence factors (yellow), GC content (pink), and GC skew (green)

The genome of *S. enterica* Typhimurium ms202 was annotated using the RAST tool kit (RASTtk) and was assigned a unique genome identifier: 90,371.4806. This genome is in the super kingdom bacteria and is annotated using genetic code 11 (Table 1). The annotation included 669 hypothetical proteins and 4,303 proteins with functional assignments, 83 transfer RNA (tRNA) genes and 10 ribosomal RNA (rRNA) genes (Table 2). 1,286 proteins with functional assignments and Enzyme Commission (EC) numbers, 1,052 with Gene Ontology (GO) assignments, 174 pseudogenes and 903 proteins were mapped to KEGG pathways. A circular graphical representation illustrated the distribution of the genome annotations (Fig. 1).

**Table 1:** Assembled genome of *S. enterica* Typhimurium ms202 showing 17 contigs with the total length of 4,894,062 bp and an average G + C content of 51.98%

Assembly	Details
Contigs	17
GC content	51.98
Plasmids	0
Contig L50	5
Genome length	4,894,062 bp
Contig N50	4,880,192

**Table 2:** *S. enterica* Typhimurium ms202 showing annotated genome using RAST tool kit (RASTtk)

Annotated genome	Features
Predicted genes	5073
Repeat regions	37
rRNA	10
tRNA	83
Miscellaneous RNA	0
Hypothetical proteins	669
Proteins with functional assignments	4303
Proteins with EC number assignments	1286
Proteins with GO assignments	1052
Proteins with pathway assignments	903

#### Phylogenetic relationship of *S. enterica* Typhimurium ms202 with other bacterial species

**Fig. 2:**
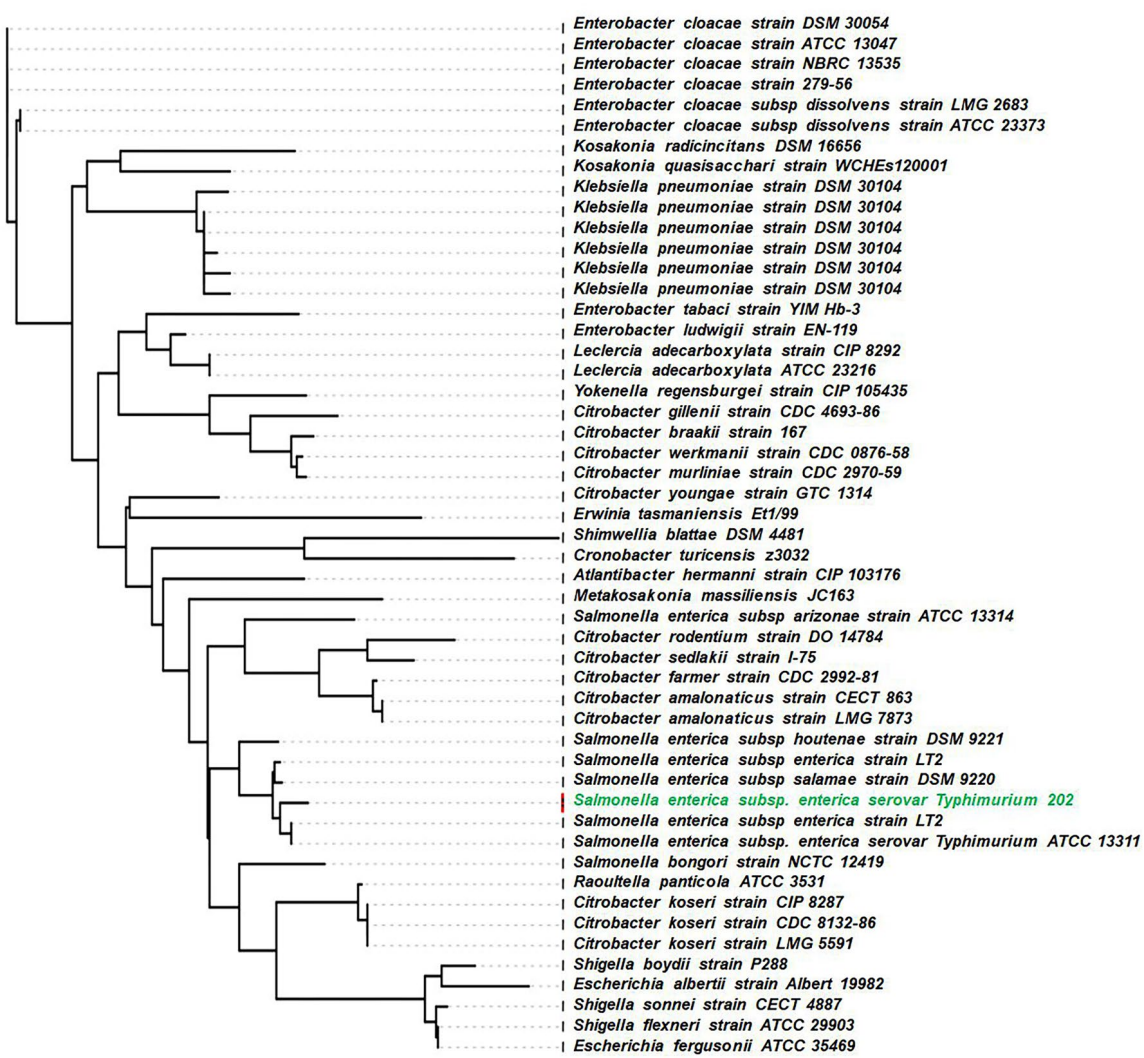
Phylogenetic position of *S. enterica* Typhimurium ms202 by neighbour-joining method for the evolutionary history of alignment. The tree contains several branches with low bootstrap support (> 50%)

The phylogenetic analysis was carried out by concatenated sequence and multiple alignments using conserved sites phylogeny of the distinct *Salmonella* species and subspecies. The Neighbour-Joining method for the evolutionary history of alignment was inferred as shown in Fig. 2. However, this tree contains several branches with low bootstrap support (> 50%) and places the two sequenced Salmonella strains such as, *Salmonella enterica* subsp*. arizonae serovar* 62:z4,z23 and *Salmonella enterica* subsp. *salamae strain* DSM 9220. Further, elaboration of phylogram shows that there are two different clusters of which one cluster contains various *S. enterica* serovars and the other has *Citrobacter freundii* CFNIH1 1,333,848.3. The phylogenetic regions indicate that *Salmonella bongori NCTC 12,419 218,493.5* is the last common ancestor, and the newly isolated strains evolved faster.

#### Comparison of Amino Acid Identity (AAI) and Average Nucleotide Identity (ANI) between *S. enterica* Typhimurium ms202 and reference

**Fig. 3:**
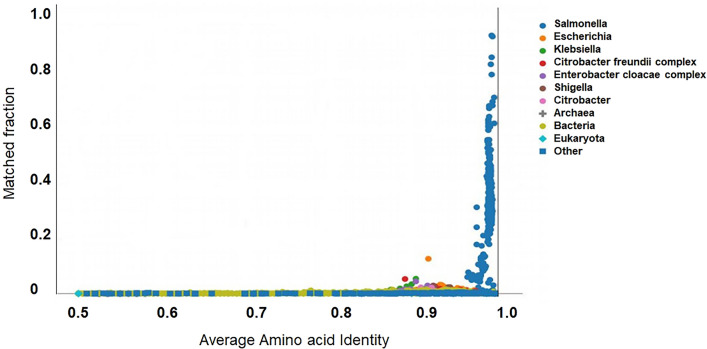
Average Aminoacid Identity (AAI)-profiler scatterplot for *S. enterica* Typhimurium ms202. The query proteome was obtained from NCBI genomes. The dot nearest coordinates (1, 1) is the query species and is closely related group which is distinct from other genera

The AAI profiler summarizes results for proteome-wide sequence search with two clear parameters obtained from pairwise genome comparisons and takes the average sequence identities of Salmonella's shared orthologous genes. The variations in the taxonomic groups indicated by colour (Fig. 3) suggested that the genome of *S. enterica* Typhimurium ms202 appears as a distinct clade with the *Citrobacter* genus. The ANI relationships between type assemblies and selection of the species boundaries are supported by 96% ANI cut-off. Further, we evaluated all taxa between taxonomy-matching (concordant) and taxonomy-non-matching (discordant) pairs selected from the taxonomically united 16S rRNA database and whole-genome assemblies. The genetic relatedness of newly isolated *S. enterica* Typhimurium ms202 was examined with two control strains such as, *S. enterica* subsp. *enterica* serovar Typhimurium*_st_*LT2 ('control 'LT2' in the current study) and *S. enterica* subsp. *enterica* serovar Typhimurium*_st_*SL1344 with (Table 3). Indeed, there was an evident distinction at 96% for most taxonomic groups observed between intra and inter-species relationship.

**Table 3:** Average Nucleotide Identity (ANI) of *S. enterica* Typhimurium ms202 and two reference genomes

Parameters	*S. enterica* subsp. *enterica* serovar Typhimurium ms 202	*S. enterica* subsp. *enterica* serovar Typhimurium_st_LT2	*S. enterica* subsp. *enterica* serovar Typhimurium_st_SL 1344
Genome total length (bp)	4,894,062	4,807,350	4,565,462
Genome coverage (%)	64.84	68.72	69.42
OrthoANIu valule (%)	98.55	99.45	98.76
Average aligned length (bp)	3,239,533	3,287,153	3,195,764

#### Functional characterization and subsystem annotation of *S. enterica* Typhimurium ms202

**Fig. 4:**
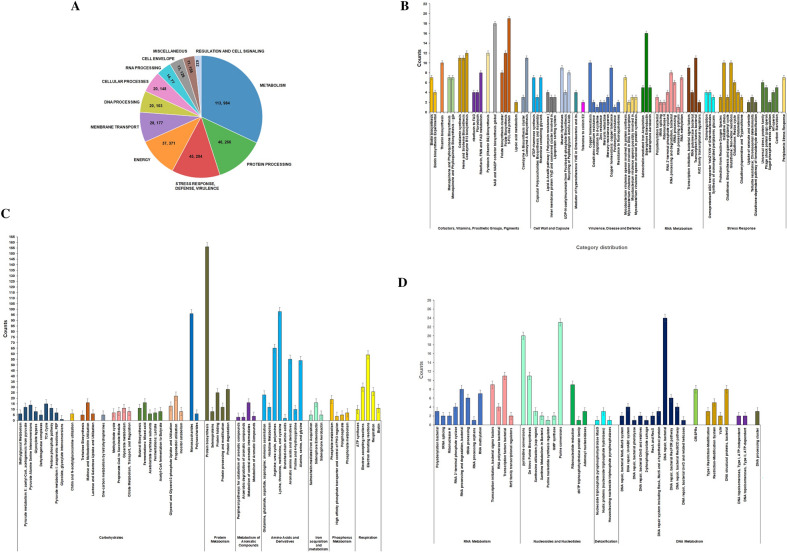
Functional Characterization and subsystem annotation of *S. enterica* Typhimurium ms202. **a** A pie chart showing gene numbers for several biological processes/physiological functions of *S. enterica* Typhimurium ms202. **b** and **c** Genes involved in sequential metabolism, **d** Genes involved in genetic material metabolism

The functional annotation of genomes was investigated. The number of protein-encoding genes associated with their active role is estimated using RAST-SEED viewer and results are shown in Fig. 4. A subsystem is a set of proteins that implements biological processes or structural complexes, and annotations include an analysis of the subsystems unique to the genome. Figure 4a represents a pie chart showing genes related to several physiological functions of bacteria. The complex processes include Metabolism (33.75%), followed by Protein processing (13.70%) and Stress response, Defense, and Virulence (13.38%). Further, we elaborated 'Metabolism' by dividing it into three sections as sequential metabolism (Fig. 4b and c) and genetic material metabolism (Fig. 4d). Most of the identified genes were supposed to be involved in carbohydrate metabolism and notably, many genes were predicted to show association with virulence and pathogenesis. The Multilocus Sequence Typing (MLST) method was used to identify seven matching genes. The length (in base pairs) of the alignment between the MLST allele referred to the database (DB) and the equivalent sequence in the input genome. (Additional file 3: Table S3).

#### Phage analyses and region annotation

**Fig. 5:**
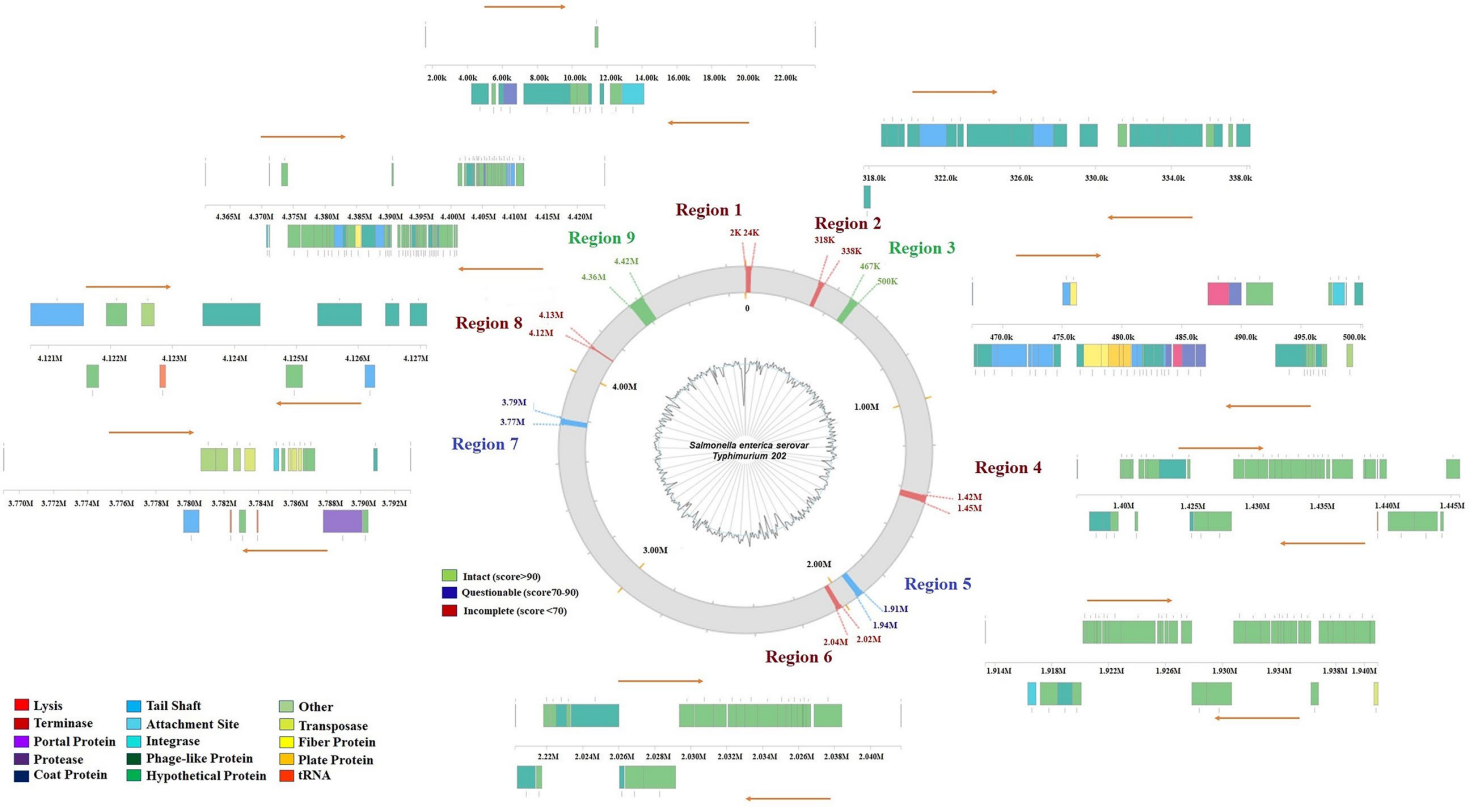
Circular presentation for distribution of phage in *S. enterica* Typhimurium ms202genome island. 9 prophage regions have been identified, of which 2 regions are intact (score > 90), 5 regions are incomplete (score < 70), and 2 regions are questionable (score 70–90) likely corresponding to phage remnants

Bacteriophages are viruses that infect bacteria and are abundant in the natural environment. They are important in regulating bacterial growth and proliferation. Further, Phages are necessary for the transmission of genetic information. In the draft genome of *S. enterica* Typhimurium ms202, nine prophage regions were found, of which two are entire, five are partial, and the other two are contested; most likely relate to phage remnants (Fig. 5, Table 4) (Additional file 4: Table S4). These phage remains are almost of same length and G + C content. Future studies are directed to compare these identified bacteriophages with phages previously reported in other bacterial species.

**Table 4:** Details of Phage sequences annotated in *S. enterica* Typhimurium ms202 genome

Region	Length	Completeness	Score	Total Proteins	Position	Most common phage	GC%
1	22.3 kb	Incomplete	40	13	1595–23,915	PHAGE_Entro_P4_NC_001609(5)	49.18
2	21.4 kb	Incomplete	10	23	2,020,249–2,041,684	PHAGE_Cronob_ESSI_2_NC_047854(14)	48.55
3	32.7 kb	Intact	150	45	467,458–500,199	PHAGE_Yersin_L_413C_NC_004745 (22)	51.75
4	29 kb	Incomplete	20	33	141,640–1,445,664	PHAGE_Cronob_ESSI_2_NC_047854 (18)	50.17
5	27.6 kb	Questionable	80	35	1,913,318–1,940,979	PHAGE_Klebsi_4LV2017_NC_047818 (19)	48.85
6	21.4 kb	Incomplete	10	23	2,020,249–2,041,684	PHAGE_Cronob_ESSI_2_NC_047854(14)	48.55
7	23.9 kb	Questionable	80	19	3,769,075–3,793,051	PHAGE_Agroba_Atu_ph07_NC_042013(2)	53.23
8	6.3 kb	Incomplete	30	10	4,120,712–4,127,102	PHAGE_Salmon_118970_sal4_NC_030919(2)	45.29
9	63.3 kb	Intact	130	66	4,361,059–4,424,378	PHAGE_Enteri_UAB_Phi20_NC_031019(19)	48.61

#### Distribution of SPIs in *S. enterica* Typhimurium ms202 genome and pathway analysis

**Fig. 6:**
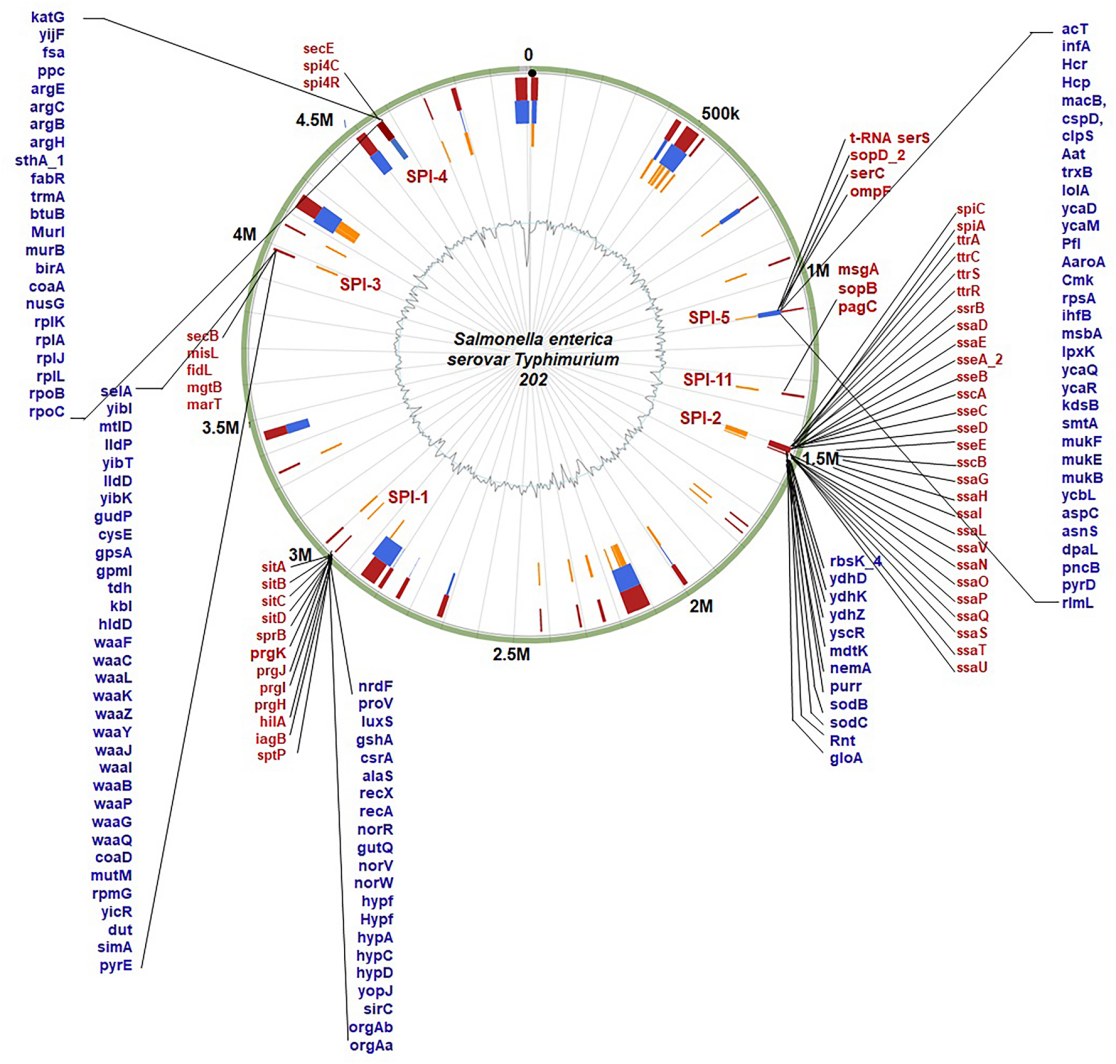
Distribution of SPIs in *S. enterica* Typhimurium ms202 genome island. Three methods such as IslandPath-DIMOB, Integrated and SIGI-HMM were utilized for virulence/resistance gene annotations. The grey colour circle represents GC content

The interpretation of virulence/resistance gene annotations in genomic islands was predicted. The whole-genome analysis revealed that *S. enterica* Typhimurium ms202 consists of six known *Salmonella* pathogenicity islands (SPI-1, SPI-2, SPI-3, SPI-4, SPI-5, and SPI-11). The distribution of the genes in SPI regions has homology to known transporters, drug targets, and antibiotic resistance (Fig. 6) and the subset of genomic islands, which facilitates the horizontal transfer of genes encoding numerous resistance and virulence factors of T3SS. Irrespective of their origin, the gene content in SPI-1 to SPI-2 is highly conserved in all the reference strains selected from the PAIs database. The *sit A* to *D genes* clusters a regulatory cascade in SPI-1 promoting virulence of bacteria. Indeed, according to the PAIs database, a maximum number of genes are encoded at SPI-2 (~ 28) followed by SPI-1 (~ 12).

Moreover, three unique genes such as *sopD_2, serC,* and *ompF* found in the SPI-5 region are mainly responsible for pathogenicity. Many SPIs are located near a tRNA gene, and differences are observed in their G + C content from the remaining part of the genome. Indeed, other virulent genes were also determined in SPI regions.

**Fig. 7:**
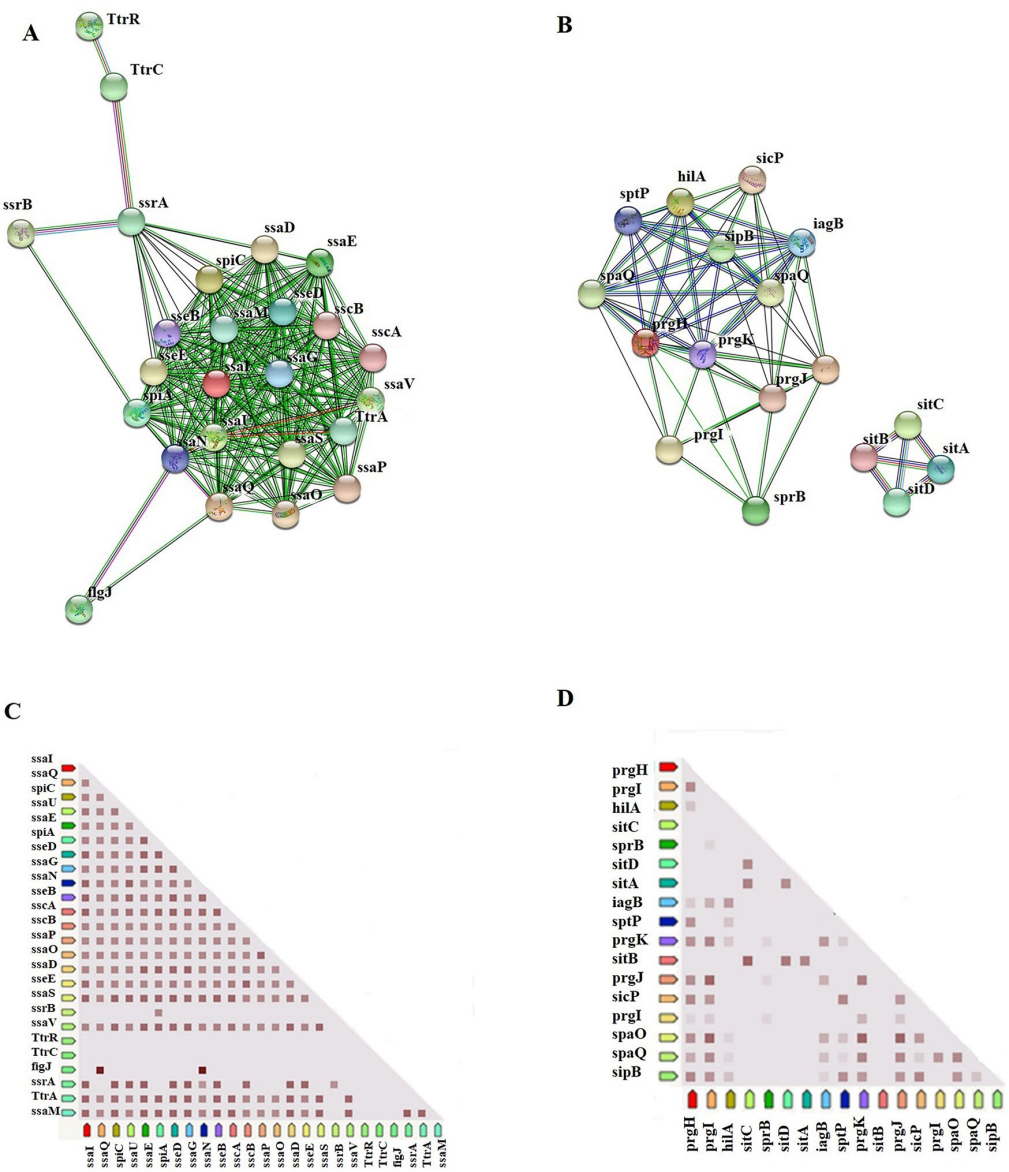
The protein–protein network cluster and co-expression profiling of the genes from SPI-1 and SPI-2 regions of *S. enterica* Typhimurium ms202. **A** and **C** SPI-2; **B** and **D** SPI-1. The red squares inside **C** and **D** represent interaction scores

The KEGG analysis showed two common pathways associated with *Salmonella* pathogenesis: the pathway of invasion into epithelial cells and flagellar assembly. However, nine different pathways were identified in SPI-5 (Table 5). For further insights, protein–protein interaction was studied by the network and co-expression based annotations. The nodes in SPI-2 [40 nodes] with average local clustering coefficient (0.904, p-value: < 1.0e-16) showed the single cluster content of approximately 20 effectors. However, *TtrR* and *TtrC* have shown string characters (Fig. 7a). None of the co-expression profiling for *TtrC* and *ssrB* was found in the SPI-2 cluster (Additional file 5: Table S5). The two different network clusters are observed in the SPI-1 region gene content (Fig. 7b). A smaller number of co-expression was identified among the effector genes consisting of *sitA, sitB, sitC, sitD*. The nodes (22 nodes) with the average local clustering coefficient (0.821, p-value: < 1.0e-16) and the co-expression profiling showed the pathogenic property and the virulence factors. For instance, the GTPase-activating protein *SptP* (Salmonella protein tyrosine phosphatase), activates SPI-4 genes and weakly represses SPI-1 genes and cell invasion protein B that are required for invasion of bacteria to host cells (Additional file 6: Table S6).

**Table 5:** The pathway analysis of the pathogenic region SPI-1, 2 and 5 in *S. enterica* Typhimurium ms202

Region	Pathways	Description	Count in network	Strength	False discovery rate
SPI-1	seni05100	Bacterial invasion of epithelial cells	5 of 9	2.05	1.04e–08
	seni05132	Salmonella infection	7 of 26	1.74	5.79e–10
	seni03070	Bacterial secretion system	2 of 38	1.03	0.0242
	seni02040	Flagellar assembly	2 of 38	1.03	0.0242
	seni02010	ABC transporters	4 of 168	0.68	0.0181
SPI-2	seni03070	Bacterial secretion system	10 of 38	1.47	2.45e–11
	seni02040	Flagellar assembly	10 of 38	1.47	2.45e–11
	seni05132	Salmonella infection	4 of 26	1.23	0.00021
SPI-5	seni00750	Vitamin B6 metabolism	2 of 9	2.15	0.00035
	seni00680	Methane metabolism	3 of 29	1.82	0.00010
	seni00260	Glycine, serine and threonine metabolism	3 of 35	1.74	0.00010
	seni01200	Carbon metabolism	3 of 116	1.22	0.0014
	seni01230	Biosynthesis of amino acids	3 of 128	1.17	0.0014
	seni02020	Two-component system	3 of 168	1.05	0.0026
	seni01130	Biosynthesis of antibiotics	3 of 216	0.95	0.0046
	seni01120	Microbial metabolism in diverse environments	3 of 265	0.86	0.0071
	seni01100	Metabolic pathways	4 of 733	0.54	0.0172

#### Annotation of antimicrobial resistance genes and virulence factors

The genes associated with virulence and pathogenicity were carried out using blast search with specific sources, and databases such as CARD, NDARO, VFDB, DrugBank were applied to avoid the limited list of identifiers and genes available in the database. The average number of genes were encoded with transporter (689), virulent factors (141) (Additional file 7: Table S7), drug target specific genes (530) (Additional file 8: Table S8), and antimicrobial resistance specific (Table 6).

**Table 6:** The genes of *S. enterica* Typhimurium ms202 with their specific functions and source database where homology was found

Specificity	Source	Genes
Antibiotic resistance	CARD	68
Antibiotic resistance	NDARO	6
Antibiotic resistance	PATRIC	60
Drug target	Drug Bank	348
Drug target	TTD	53
Transporter	TCDB	689
Virulence factor	PATRIC_VF	318
Virulence factor	VFDB	122
Virulence factor	Victors	310

The Genome Annotation Service in PATRIC uses a *k*mer-based method to detect antimicrobial resistance (AMR) gene variants. To each AMR gene, this service assigns functional annotation, comprehensive antibiotic resistance mechanism, different classes of drugs and occasionally, a specific antibiotic to which it confers resistance. It notes that the presence of AMR-related genes (even full length) in a given genome directly implies an antibiotic-resistant phenotype. It is essential to consider specific AMR mechanisms, especially regulator modulating expression, efflux pump conferring, and target in susceptible categories (Table 7). Noteworthy, seven genes such as *fosA7, tet(A), sul1, aadA7, aac('6')-iaa, aadA7,* and *qacE* were identified as the acquired antibiotic resistance genes in currently studied drug resistant strain. (Table 8).

**Table 7:** Genes of *S. enterica* Typhimurium ms202 involved with antimicrobial resistance mechanisms

AMR mechanism	Genes
Antibiotic activation enzyme	KatG
Antibiotic inactivation enzyme	AAC(6')-Ic,f,g,h,j,k,l,r-z
Antibiotic resistance gene cluster,cassette,or operon	MarA, MarB, MarR
Antibiotic target in susceptible species	Alr, Ddl, dxr, EF-G, EF-Tu, folA, Dfr, folP, gyrA, gyrB, inhA, fabI, Iso-tRNA, kasA, MurA, rho, rpoB, rpoC, S10p, S12p
Antibiotic target protection protein	BcrC
Efflux pump conferring antibiotic resistance	AcrAB-TolC, AcrAD-TolC, AcrEF-TolC, AcrZ, EmrAB-TolC, MacA, MacB, MdfA/Cmr, MdtABC-TolC, MdtL, MdtM, MexPQ-OpmE, OprM/OprM family, QacE, SugE, Tet(A), TolC/OpmH
Gene conferring resistance via absence	gidB
Protein altering cell wall charge conferring antibiotic resistance	GdpD, PgsA
Regulator modulating expression of antibiotic resistance genes	AcrAB-TolC, EmrAB-TolC, H-NS, OxyR

**Table 8:** Acquired antibiotic resistance genes in *S. enterica* Typhimurium ms202

Resistance gene	Identity	Alignment length/Gene length	Contig or Depth	Position in contig	Phenotype	PMID
fosA7	97.164	423/423	K141_120 flag = 0 multi = 158.20 len = 31,263	27,958..28380	Fosfomycin	26,083,489
sul1	100	840/840	K141_152 flag = 1 multi = 135.89 len = 61,328	5235..6074	Sulfamethoxazole	–
aadA7	98.99	798/798	K141_152 flag = 1 multi = 135.89 len = 61,328	6579..7976	Spectinomycin, str.eptomycin	10,817,710
aac(6’)-laa	97.94	438/438	K141_133 flag = 0 multi = 135.89 len = 1,937,198	151,206..151643	Amikacin, tobramycin	11,677,609
tet(A)	100	1200/1200	K141_2 flag = 0 multi = 132.29 len = 55,544	50,235..51522	Doxyxyxline, tetra cycline	12,654,659
aadA7	98.99	798/798	K141_152 flag = 1 multi = 135.89 len = 61,328	6579..7376	Spectinomycin, str.eptomycin	10,817,710
qacE	100	282/333	K141_152 flag = 1 multi = 135.89 len = 61,328	6134..6415	Benzylkonium chlorie, chlorhexidine, cetylpyridinium chloride	8,494,372

#### Expression profile of virulence genes in *S. enterica* Typhimurium ms202

**Fig. 8:**
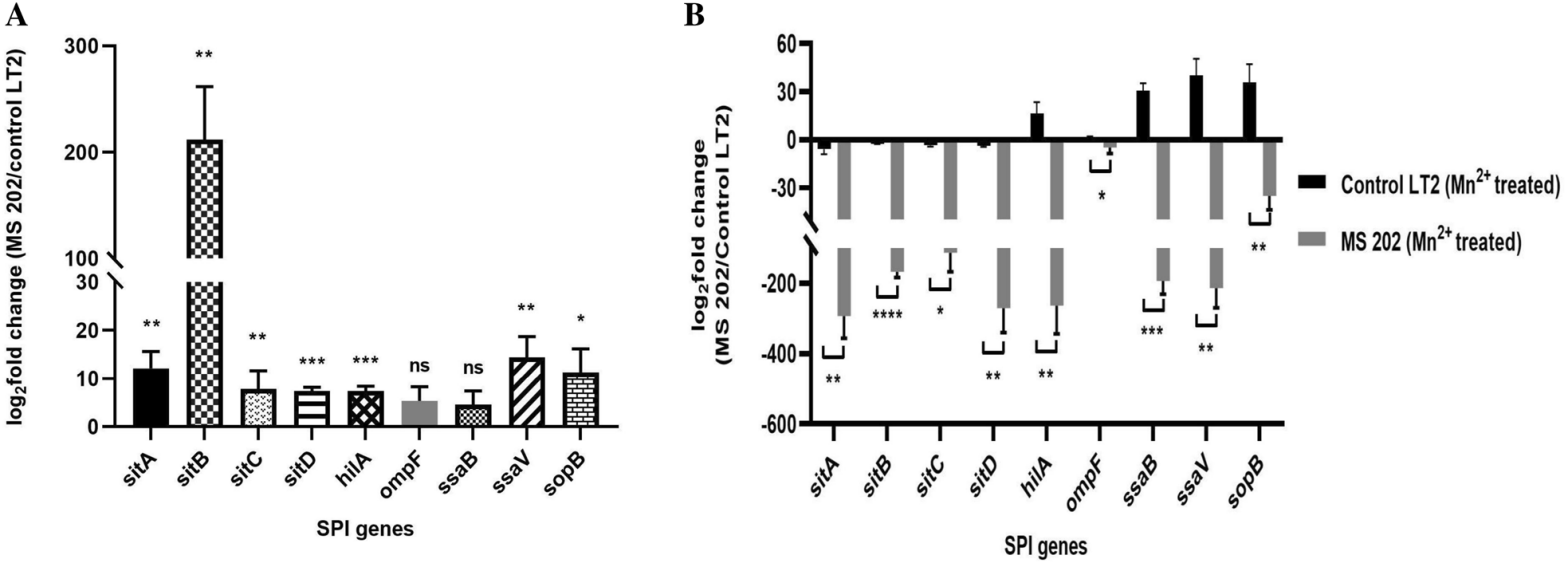
Expression profile of virulent genes in *S. enterica* Typhimurium ms202 under the influence of Mn^2+^. **A** Expression levels of nine different virulent genes encoding SPI1 (*sit A, B, C, D*, *hil*A, *ompF* and *ssaV*) and SPI2 (*ssaB* and *sopB*) regions of *S*. Typhimurium by qRT-PCR. Results are expressed as log_2_ fold change in *S. enterica* Typhimurium ms202 with respect to control LT2. 16 s rRNA gene was considered as a housekeeping gene in qRT-PCR. Error bars indicate Mean ± SE. The student's t-test determined statistical significance, and P < 0.05 was considered significant. **B** Expression levels of nine different virulent genes encoding SPI1 (*sit A, B, C, D*, *hilA*, *ompF* and *ssaV*) and SPI2 (*ssaB* and *sopB*) regions of *S*. Typhimurium by qRT-PCR after treatment of with 5uM MnCl_2_ compared to corresponding untreated controls. The 16 s rRNA gene was taken as the housekeeping gene in qRT-PCR. Error bars indicate Mean ± SE from three independent experiments. Statistical significance was determined by Student's t-test, and P < 0.05 was considered as significant

To examine if *S. enterica* Typhimurium ms202 has higher degree of virulence as compared to drug sensitive strain, we compared the expression profile of 9 different genes (Identified in the genome draft) associated with virulence of bacteria encoding SPI-1 (6 genes), SPI-2 (2 genes) and SPI-5 (1 gene) regions of the bacteria with that of control LT2. These genes were selected arbitrarily from the genome draft results, emphasizing SPI-1 encoding genes due to their vital role in the invasion and colonization of *Salmonella* in host gut. Specifically, four specific SPI-1 encoding genes such as, *sitA, B, C* and *D* are known to play a role in transporting ions such as Mn^2+^ and Fe^2+^ along with virulence function. Figure 8A shows that genes such as *sitA, sitB, sitC, sitD, hilA* and *sopB* encoding SPI-1 and *ssaV* under SPI-2 were increased by 12.1 fold, 212 fold, 14.4 fold, 7.3 fold, 7.4 fold, 11.3 fold and 14.3 fold respectively in *S. enterica* Typhimurium ms202 as compared to control LT2 indicating a significant upregulation of gene expression in the drug-resistant strain (P < 0.05). Although genes such as *ompF* (encoded by SPI-5) and *ssaB* (encoded by SPI-2) were upregulated by 5.5 fold and 4.9 fold, the difference was not statistically significant. An overall increase in virulent gene expression in *S. enterica* Typhimurium ms202as compared to control suggested a higher virulence potential of the MDR strain.

To understand if greater virulent potential in *S. enterica* Typhimurium ms202 results in an efficient utilization of metallic ions available in hosts, we examined the expression profile of all 9 genes in the drug resistant and control strain in the presence of exogenously added Mn^2+^ in culture media. Results shown in Fig. 8B revealed that *sitA, B, C,* and *D* (Mn^2+^ transporter genes) were downregulated in Mn^2+^ treated control LT2 than the untreated control. There was a 5.8 fold, 2.6 fold, 3.5 fold, and 3.7 fold decreased expression of these four genes in control LT2 exposed to Mn^2+^ compared to untreated ones. On the contrary, levels of *hilA*, *ssaB*, *ssaV* and *sopB* genes were significantly upregulated in treated control LT2 (P < 0.05). *OmpF* gene was found marginally increased (P > 0.05). Importantly, we found all 9 virulent genes significantly downregulated in the Mn^2+^ treated drug-resistant strain as compared to untreated *S. enterica* Typhimurium ms202. There was a 292.6 fold decreased expression of *sitA* in treated ms202 compared to untreated strain. In contrast, other genes such as *sitB, C, D*, *hilA, ssrB, ssaV* and *sopB* were downregulated by 167.5 fold, 113.4 fold, 270.6 fold, 263.7 fold, 193.5 fold, 214 fold and 35.3 fold in the drug-resistant strain. A marginal decrease in the *ompF* level (4.9 fold) was observed (P > 0.05). Ultimately, when compared between treated *S. enterica* Typhimurium ms202 vs treated LT2, a notably decreased expression of all 9 genes was observed in drug-resistant strain (P < 0.05).

## Discussion

Studies using whole-genome sequence showed the serovar-level distribution in *Salmonella enterica* subsp *enterica* [16–18]. However, it has often been neglected and studies on in-depth pathway analysis regarding the NTS infections are rarely reported from India [19]. As noted earlier, *S*. Typhimurium is the most common pathogen for NTS infection, significantly contributing to gastroenteritis with a greater intensity of infection in immunocompromised hosts [20, 21]. As the resistance of *Salmonella* to multiple antibiotics has become a global issue disabling treatment options, detailed analysis of the genome of drug resistant strain is crucial to analyse new virulence factors and AMR genes. Accordingly, we were interested in exploring genome annotation of the multidrug-resistant clinical isolate, *S. enterica* Typhimurium ms202. Here, the *de-novo* genome assembly has shown better annotation with fewer gaps, genome coverage and better scaffold statistics. The study sheds light to identify the distributions of SPIs and the operon of virulent factors that might help to understand the pathogenic nature of this newly identified strain and information on antimicrobial-resistance [22].

In general, 16 s rRNA sequencing-based phylogenetic-tree analysis is the most frequently used method to classify organisms. In our phylogenetic tree analysis, we have identified that *S. enterica* Typhimurium ms202 has 99.9% similarity to *S. enterica* subsp. *salamae* strain DSM 9220 and *Salmonella enterica* subsp. *enterica* strain LT2. The study on comparative analysis indicated a close relationship among these bacteria [23] and suggested the association with disease in humans and animals [24]. Remarkably, *S. enterica* Typhimurium ms202 and *S. enterica* serovar Arizonae have also supported coalescing and sharing core serological properties, but the latter is not a common pathogen in humans and shows occasional disease association. From phylogenetic tree analysis, we find that *S. enterica* Typhimurium ms202 is far from *S. bongori* The distinct taxonomic grouping defines the genealogical species concept for *S. enterica* Typhimurium ms202 [25] and emphasizes the understanding of genetic diversity and phylogenicity among *S. enterica* serovars [18].

The previous study has shown that *S.* Typhimurium carried the 90 kb virulence plasmid [26]. However, the role of the virulence plasmid in pathogenesis is not clear [27]. It is suggested that phage remnants in the *Salmonella* genomes contribute towards both inter-species and inter-strain variability. Indeed, we didn't find any phage plasmid in *S. enterica* Typhimurium ms202. According to earlier reports, plasmids are often identified in *S.* Typhimurium and *S.* Enteritidis strains isolated from blood and other extra-intestinal sources than strains isolated from faeces [27–29]. The absence of phage plasmid in our MDR strain may be due to its isolation from feces. Moreover, a different framework is needed on the sequence, database, and tool limitations to overcome assessing the plasmid populations within *S. enterica* Typhimurium ms202.

MDR *Salmonella* has become a global threat, including India. Therefore, non-antibiotic approaches such as the use of bacteriophages (phages) has gained attention and may be effective in the treatment and prevention of infection caused by antibiotic-resistant bacteria. *Salmonella* consists of various phage receptors such as flagella, O-antigen, OmpC, FhuA, and TolC. The complex nature of lipopolysaccharide (LPS) of *S.* Typhimurium and the receptor diversity may block access of phage to some outer membrane proteins and receptors, which causes the host *S.* Typhimurium to become resistant to phage [30]. Herein, we identified nine phages and the genes mapping to NC_004745 and NC_031019 revealed the presence of a set of virulent factors translocated into the bacterial cytoplasm through the outer membrane [31, 32]. Further studies may insight the receptor interaction mechanisms between the phage and host that may lead to optimal phage therapy for protection against the MDR *S.* Typhimurium.

We summarized the distribution of the genes (*k*mer-based method) based on their antimicrobial resistance mechanisms. The maximum number of genes were identified in efflux pumps conferring antibiotic resistance and antibiotic targets in susceptible species categories. The efflux pumps play a vital role in drug resistance and serve other *Salmonella* functions [33]. Extensive studies on the AcrAB-ToIC system in *Escherichia coli* and *Salmonella* confer innate resistance to many antibiotics [34, 35]. Further evidence suggested that, *S.* Typhimurium isolate derived from a patient with ciprofloxacin treatment overproduced AcrAB [36]. We have also identified seven acquired antibiotic resistance genes. A previous study has reported that phage populations in *S. enterica* contribute to horizontal gene transfer, including virulence and virulence-related genes within the subspecies [37–40].

The objective of our study was to reveal the genome elements of the newly isolated *S. enterica* Typhimurium ms202 that may contribute to virulence and resistance**.** Prior reports suggested that SPI-1, SPI-2 encoding T3SS, SPI-3, SPI-4, SPI-5 and type VI secretion systems play an essential role in association with the host cell, invasion, and the intracellular lifestyle of the Salmonella [32, 41]. The SPI 1–5, 9, 13 and 14 are the core part of the genome, whereas SPI-6, 10–12, 16–19 are variable among genomes, and SPI-7, 8 and 15 are known to be absent in invasive non-typhoidal *Salmonella* [42]. In our case, the whole-genome comparison results revealed *S. enterica* Typhimurium ms202to harbour six pathogenicity islands (SPI-1 to SPI-5 and SPI-11). However, SPI-9, 13 and 14 were not detected. Indeed, the absence of a few SPIs has also been reported earlier. For example, Blondel et al. (2009) reported the lack of SPI-19 to 21 in both *S.* Typhimurium and *S.* Typhi [43]. Hence, these differences with previous reports may be due to different isolates and serotypes, methodologies, and SPI- host specificity [44, 45]. Our report has shown the significant diversity within the SPI-1, 2 and 5 in gene content. The *sit A* to *sit D* genes cluster regulates/triggers the growth and virulence of *Salmonella* Typhimurium by receiving specific environmental cues such as organic iron, calcium, and magnesium [46, 47].

**Fig. 9:**
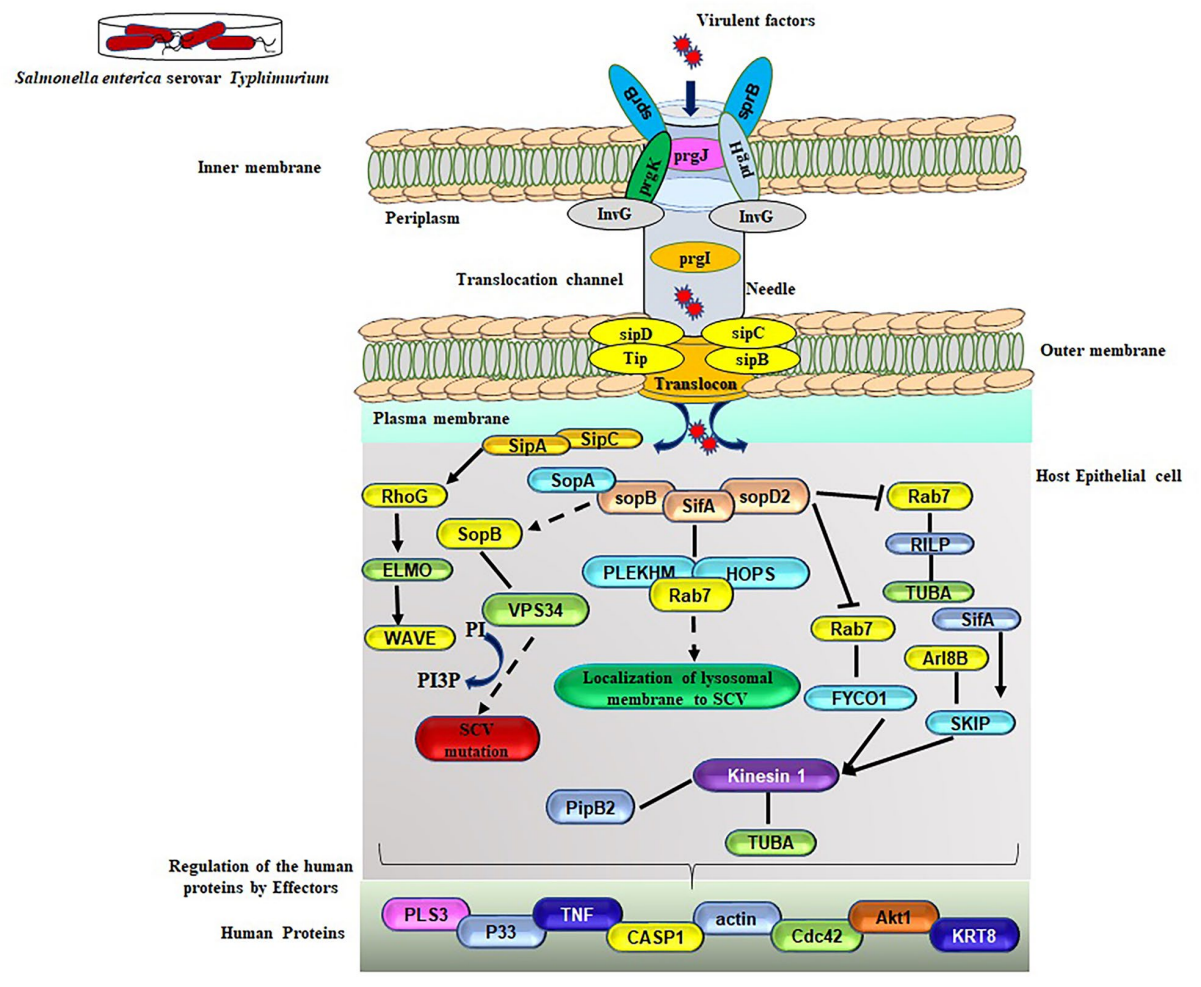
Mechanism of sequential activation of *Salmonella* infection and regulation pathways. *HilA* is the central regulator of SPI-1 and T3SS needle apparatus in association with the host cell. Salmonella invasion proteins (Sips), A–D mediate SPI-1 effectors secretion and translocation. After translocation into host cells, the Salmonella outer proteins (Sops) are involved in polymorphonuclear leukocyte influx. After crossing the epithelial barrier, T3SS encoded SPI-2 effector proteins are secreted into the cell's cytosol for gene expression. The pathogen first recognizes these influence factors in the distal gut lumen and later in the intra-macrophage environment

SPI-1 plays a vital role in the colonization and invasion of *Salmonella* in the gut and the recruitment of neutrophils [44, 45]. We identified the *IagA* and *hilA* encoded in pathogenicity island SPI-1, that are associated with regulation of invasion and activate the expression of *prgHIJK*. The protein *prgH* is unique to the *Salmonella* SPI-1 and makes up the membrane-spanning needle complex with *InvG*, *prgI*, and *prg K*. This complex regulates the production of effector proteins from *Salmonella* into the host cell [48, 49]. Indeed, the central regulator of SPI-1 expression is correlated with the *hilA* transcription factor [50]. To gain further insights into the organization of the this MDR strain genomic SPI-2 region, we further analyzed the virulent proteins. We found that SPI-2 effector proteins such as *sseB, sseD* and *sse F* are essential for the appropriate localization of *sseC* and *sseD* on the cell surface of bacteria, causing translocation of effecter protein into the host cell and aggregation of the host endosomes, respectively (Fig. 9). Several studies have indicated that the protein encoded genes from ssa family members belong to T3SS involved in Salmonella's replication and intracellular survival in the host. In contrast, *ssrA* and *ssrB* are indispensable for the SPI-2 encoded virulence gene expression. The transcriptional regulator of *sprB* is primarily reported in SPI-4 and weakly represses SPI-1 too. This study not only has recapitulated reports from the previous investigations about the serotypes and distribution of genes in SPI regions but also verified their profiles using the database for *S. enterica* Typhimurium ms202 [19, 51, 52].

The survival of bacteria inside hosts is solely dependent on its virulence potential and ability to utilize host resources. Pathogenic drug-resistant bacteria are known to display a better degree of survival advantage inside hosts than a drug-sensitive strain and one obvious reason is inherent ability of the former to resist antibiotics. *Salmonella* Typhimurium acquires different micronutrients (Examples—Mn^2+^, Fe^2+^, Zn^2+^) from the host milieu through metal transporters, and these ions contribute to the virulence and survival of bacteria [53]. However, it is not known how drug-resistant *Salmonella* with a better survival strategy in hosts behaves to this physiological function (acquiring metal ions from the host) and we addressed the issue in the current study. [We deciphered that, probably this drug resistant bacterium is more efficient in acquiring metal ions (Mn^2+^) from an external source due to higher degree of virulence. The entire issue is addressed as follows.

It is known that, in the presence of Mn^2+^ in the host milieu, *S*. Typhimurium acquires these divalent ions and *hilA* is activated that further regulates other SPI-1 associated virulence genes [53]**.** However, when Mn^2+^ is absent in the host environment, the pathogen cannot acquire the required ions leading to the inactivation of *hilA*. In this case, Mn^2+^ transporter genes such as *sit A, B, C* and *D* modulate virulence and adaptation to stress response. They are found to be upregulated in bacteria and serve as SOS responses. In our study, we found a significant upregulation of 7 out of 9 different SPI-1, SPI-2 and SPI-5 genes in *S. enterica* Typhimurium ms202 strain as compared to control LT2, indicating a possible role of these genes in contributing to a higher degree of virulence of ms202 (Fig. 8A). However, future studies are directed to evaluate the degree of virulence of these bacteria by in vivo phenotype validation experiments.

Our second important observation was a significant reduction in *sit A, B, C* and *D* genes in control LT2 in the presence of Mn^2+^ in M9 media as compared to ion deficient condition (Fig. 8B). These results are in accordance with previous studies by [54], which showed significant repression of *sit ABCD* operon of S. Typhimurium on the addition of Mn^2+^ to bacterial culture due to regulatory activity of mntR gene. During data analysis for *S. enterica* Typhimurium ms202, we found a similar trend (reduction of these four Mn^2+^ transporter genes in Mn^2+^ supplemented ms202 as compared to ion deficient condition), however to a greater extent. Importantly, we observed a significant fold reduction for all four *sit* genes in *S. enterica* Typhimurium ms202 compared to control strain LT2 when both types of bacteria were exposed to Mn^2+^. In this context, two possibilities can be discussed to explain higher fold reduction of genes in drug resistant stain on manganese exposure. First, reduction of gene expression may be due to the higher efficiency of *S. enterica* Typhimurium ms202 to acquire these divalent ions from external sources due to their higher virulent potential compared to control LT2 (as shown in Fig. 8A). Second, it is known that, the regulatory gene, *mntR* serves as a primary sensor for Mn^2+^ abundance leading to repression of *sit* ABCD genes in *S.* Typhimurium [54]. Therefore, *mntR* could be playing a regulatory role resulting in higher fold reduction of *sit* gene expression in Mn^2+^ supplemented *S. enterica* Typhimurium ms202, supposedly through an unknown mechanism that needs to be looked at in future.

Although our data showed an apparent down-regulation of four *sit* genes (possessing a role for both Mn^2+^ transport and virulence) in control LT2 on exposure to Mn^2+^. Four other genes, such as *hilA*, *ssaB, ssaV* and *sopB* (possessing a role in virulence but not in ion transport), showed a significant upregulation in bacteria upon exposure to Mn^2+^. Although the reason is not very clear to us, previous studies have indicated a firm requirement of different micronutrients by bacteria for their metabolism and virulence factor expression [47, 55]. Thus, it is assumed that adding Mn^2+^ to the culture might have contributed to the higher expression levels of few virulent genes, supposedly playing no role in ion transport. In contrast to these results, our observation showing a significant downregulation of these four specific genes (*hilA, ssaB, ssaV* and *sopB*) in Mn^2+^ exposed drug-resistant *S. enterica* Typhimurium ms202 compared to untreated bacteria is not properly understood.

## Conclusions

This study sheds light on the genome draft of a new MDR *Salmonella enterica* serovar Typhimurium ms202 strain isolated from fecal sample of a patient with gastroenteritis and emphasizes the contribution of genetics architecture to antibiotic resistance and virulence. We found that the isolate commonly carried six SPIs, virulence operon, several MDR genes and phages. These genetic elements pose insight on the strain characteristics and allows further understanding of the diversity, host specificity of SPIs, and pathogenicity of *S. enterica* Typhimurium ms202. These features may promote a quicker and more accurate control of the food-borne illness outbreaks in eastern coastal areas of India. Further, the study also unravels a clue for a higher degree of virulence property as demonstrated by enhanced expression of several virulent genes. It is further observed that, due to a higher virulent property, this MDR bacterium showed an increased efficiency for acquiring Mn^2+^ ions from the external environment as indicated by a drastic reduction in Mn^2+^ transporter genes. Future study may be designed to find gene targets in drug resistant *Salmonella* using to which host derived molecules (as sensors) may activate causing reduction of virulent features of drug-resistant bacteria ultimately resulting in decreased pathogenesis.

## Methods

### Study design


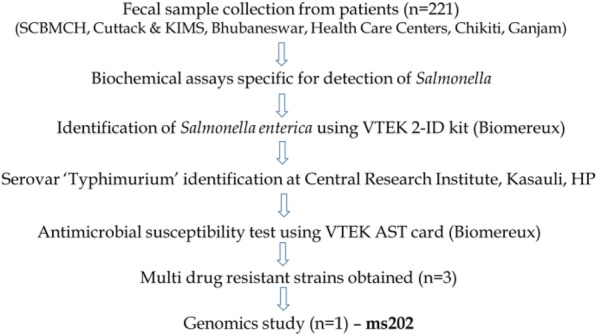


The overall design of our study is mentioned as a workflow shown above. During a study period from September 2018 to December 2019, fecal samples could be collected from 221 patients (aged 1–80 years of age) suffering from acute gastroenteritis (Subjects with loose motion > 3 times or one large voluminous stool in 24 h, with/without dysentery) admitted in SCB medical college, Cuttack, India and Pradyumna Bal Memorial Hospitals (KIMS), Bhubaneswar, India and. The study only included in-house patients and OPD cases were excluded as sample collection was not possible from later case due to their temporary stay in hospitals.—Among 221 study subjects, 6 of them had concurrent complication such as acute renal failure presenting anuria or oliguria with altered mental status due to electrolyte deficiency. Upon admission into hospital, symptomatic treatment (drugs for oral rehydration, probiotics etc..) was initiated for patients and all subjects were undergone routine microscopic and biochemical analysis in feces. Patients demonstrating bacteria in stool samples were undergone treatment with appropriate antibiotics after analysing the test reports. All study aspects were performed following National Ethics Regulations and were approved by Institutional Review Boards of the hospitals taken under the study.

### Identification of *S.* Typhimurium in feces of patients

Stool samples from patients suffering from gastroenteritis were collected in Cary-Blair transport media (M202, Hi Media), and were brought to laboratory within 2–3 h of collection.. To detect Salmonella, procedure by [56] was followed. Briefly, feces were first pre-enriched in Selenite Cysteine Selective enrichment broth (LQ070, HiMedia) for 18–24 h at 35^ °^C. This process enables the detection of *Salmonella* in the non-acute stages of illness and further inhibits the growth of competing non-*Salmonella* faecal coliforms and enterococci. After appropriate growth, bacteria were sub-cultured two times on plate with XLT4 selective agar (M1147, HiMedia); first sub-cultured after 6–8 h and then after 18–24 h of incubation at 35^ °^C. *Salmonella* was identified by examining a black centred colony on the agar plate surrounded by a yellow-coloured halo. These colonies were further inoculated in the Triple Sugar Iron agar (TSI) (MM021, HiMedia), Citrate agar (M728, HiMedia) and Urea agar (M112S, HiMedia). After overnight incubation, organisms demonstrating an adverse urea reaction and a positive TSI and citrate reaction were considered *Salmonella*. These isolates were further analyzed using VTK 2 ID card (Biomeriuex, Germany) in VITEK II COMPACT instrument to confirm *Salmonella enterica*. To identify serovar ''Typhimurium', *S.* enterica isolates were sent to Central Research Institute, Kasauli, Himachal Pradesh, for serotype analysis. The collected isolates were stored at − 80^ °^C with 30% glycerol in phosphate-buffered saline until further use.

### Antimicrobial susceptibility test for *S.* Typhimurium

The resistance pattern of *Salmonella* Typhimurium to several antibiotics was examined using VITEK II COMPACT AST card (a device to examine antibiotic susceptibility of bacteria belonging to Gram -ve Enterobacteriaceae family; Biomereux, France) in VITEK II COMPACT instrument. Bacteria showing resistance to three or more than three groups of antibiotics were considered MDR strains. One MDR isolate (*S. enterica* Typhimurium ms202) was used for current genome analysis study that showed resistance to the highest numbers (four) of antibiotic groups.

### De novo genome sequencing, quality control and pre-processing of FASTQ files

According to the manufacturer's instructions, genomic DNA extraction was performed from overnight cultures using QIAamp 96 DNA QIAcube HT Kit (Qiagen, Gaithersburg, MD). The DNA integrity was determined by agarose gel electrophoresis and the A260/280 ratio used for purity check. Followed by the usage of Illumina TruSeq DNA LT library kit was used for preparing the genomic library. Whole-genome sequencing was conducted utilizing the Illumina MiSeq system (Illumina, San Diego, CA) using the SBS sequencing kit V3.0, with a 2 × 100 paired-end option.

Downstream analysis requires Quality control and pre-processing of FastQ files to obtain clean data. The FastQC program [14] developed in C^++^ containing multi-threading support, is used for quality control and features for data-filtering. The quality of the raw reads was checked using FastQC (0.11.5), followed by trimming of adapters and low-quality bases with a Phred quality score of less than 20. Briefly, Trimmomatic [57] (*k*-mer 41) were applied to remove the low-quality reads (average quality scores < 20) and adapter sequences and the clean reads assembled.

### Primary assembly of the raw data validation

The purpose of a sequence assembler is to create lengthy continuous sections of sequence (contigs) from several reads. Contigs are sometimes arranged and positioned in reference to one another to build scaffolds. The high quality (HQ) filtered reads from sample were assembled using the SPAdes assembler with the default parameters [58]. Furthermore, three distinct assemblers, ALLPATHS-LG, ABySS, and SOAPdenovo2 were used and an assembly assessment was done, despite the fact that the assembly methodology differed in approaches.

RNAmmer 1.2 4 was used to predict the closest reference check based on the 16 s rRNA gene sequence from the generated main genome assembly [59]. The projected 16 s rRNA gene sequence was used to find the closest genome reference for the newly generated genome assembly using EzBiocloud. The assembled genome FASTA file was used as a reference, and the filtered HQ reads from sample were aligned with respective assembled genome using the aligner Bowtie2 (v 2.2.2) to validate the percentage of HQ reads used to make the respective draft genome assembly.

### Assembly of the final genome draft and prediction of phylogeny position

The assembled primary genome FASTA and the closest reference for the assembled primary genome were aligned with MAUVE aligner version 2.4.0 using the progressive algorithm with default parameters. The contigs of the primary assembled genome are rearranged using the web-based tool MeDuSa for ordering and orientating assembled genome contigs FASTA file into scaffold with the default web interface parameters. [MeDuSa uses information obtained from a set of (draft or closed) genomes from related organisms to determine the correct order and orientation of the contigs]. This process results in a total length of 2.4 Gb (contig N50: 48.8 kb and scaffold 12.8 Mb). The projected 16S rRNA sequence was aligned to the NCBI 16S rRNA database, and the top 50 aligned hits were used to construct a tree. All sequences were concatenated, and several alignments were made through the use of conserved locations. MAFFT version 7 was used to create the phylogeny. The scale reflects the predicted number of substitutions per site based on the best-fitting GTR2 + F0 + R2 (binary) model.

### Prediction of Average Nucleotide Identity (ANI) and Average Amino Acid Identity (AAI)

An Average Nucleotide Identity (ANI) above 95% between two genomes indicates that they belong to the same species. ANI was calculated with the nearest reference genome for the assembled genome of the sample using ChunLab's online Average Nucleotide Identity (ANI) calculator.

One-sided and bidirectional AAI profiles were generated for a query proteome (protein sequences in FASTA format). To extract homologous proteins from Uniprot, SANSparallel. Taxonomic information was employed obtained via the DictServer. Each query protein was matched to all database species with the highest bitscore. One-sided AAI profiles are based on a many-to-one mapping from query proteins to database species target proteins. Multiplicity was defined as the number of query proteins showing a match in the target species divided by the number of different target proteins. Bidirectional AAI profiles are based on a one-to-one mapping; in which we eliminate the match of a query protein to a database protein if a better score match occurs for either sequence. AAI is the average of all matches between the query proteome and a database species, with each query protein having a weight of one. SANSparallel reports that query proteins with no matches have zero weight.

### Draft for gene prediction, annotation, and functional characterization

RAST is a fully automated service that provides high-quality genome annotations for annotating complete or nearly complete bacterial and archaeal genomes across the whole phylogenetic tree. The draft genome was subjected to RAST annotation server (Rapid Annotation using Subsystem Technology) for gene prediction & annotation [60] with annotation parameters such as Genetic code = 11, E Value cut-off for selection 0 pinned Metricd CDSs = 1e–20.

### Plasmid and phage screening

A tool, PlasmidFinder 2.0.8, was used to identify the plasmid sequence, if any, in the ms202 draft genome.—Sequences of phages were screened in the draft genome using the tool PHASTER (PHAge Search Tool Enhanced Release) [61].

### Identification of antimicrobial resistance genotypes and genes for virulence

We compiled a list of genes associated with drug resistance and virulence identified from earlier reports in the NCBI database, Perl scripts to perform local BLASTn with ResFinder (https://cge.cbs.dtu.dk/services/ResFinder) and AMR Finder Plus with the default settings. Besides, the predicted protein sequence from the assembled genome was screened individually for antibiotic resistance genes using the Web portal—RGI 5.1.1, CARD 3.1.0. The sequences showing < 100% identity or sequence length were further examined by additional BLAST analysis.

### Detection of SPI regions in the genome of *S. enterica* serovar Typhimurium ms202

The genes in the genomic island use strategies for genomic comparison analysis associated with specific and valuable functions in bacteria. The SPIs were detected from the assembled scaffold using Pathogenicity Island (PAI) database and bioinformatics pipeline. In brief, the sequences of all the 23 SPIs were subjected to the BLAST search of the assembled genomes versus the SPI local database [62]. Further, IslandViewer4 integrates the Islander curated database to identify unique regions in the related organism's genomes and reveals genes showing features for virulence, pathogenicity, drug resistance, and homologous factors.—The IslandPath-DIMOB, Integrated, SIGI-HMM methods were used for annotations of virulence/resistance genes.

### Protein–protein interaction cluster and co-expression profiling

Kyoto Encyclopedia of Genes and Genomes (KEGG) was used for pathway mapping (www.genome.jp/kegg). DAVID's Expression Analysis Systematic Explorer (EASE) tool was used to investigate and represent functional groups. The Interaction networks and the co-expression profiling were carried out using STRING online software [63]. All associations in STRING are provided with a probabilistic confidence score representing a functional linkage among the proteins. We have selected proteins from the SPI regions whose genes are observed to be correlated in the expression.

### Bacteria and growth conditions

*Salmonella enterica* Typhimurium MDR strain, ms202 and *Salmonella enterica* Typhimurium strain LT2 (hereafter termed as 'control LT2 'strain', ATCC, 700,720) were grown in M9 minimal media (20% 5X M9 salts) (G013, HiMedia), or M9 supplemented with 5 µM MnCl_2._ [This concentration of MnCl2 selected following study by [64]. 1% of overnight culture inoculum of bacterial strains were then sub-cultured in minimal media and grown at 37℃ and 150 rpm. The absorbance at 600 nm was monitored at every 30 min interval using UV–Vis spectrophotometer till optical density (O.D.) = 0.6 was achieved.

### RNA Isolation from *S. enterica* Typhimurium ms202

Both LT2 and *S. enterica* Typhimurium ms202 strains attending log-phase of growth in M9 minimal medium (G013, HiMedia) or M9 supplemented with Mn^2+^ were collected. RNA was isolated following the methodology by [65]. RNA was quantified using a Colibri microvolume spectrometer (Titertek Berthold) and normalized for cDNA synthesis. cDNA synthesis was carried out using the Verso cDNA synthesis kit (Thermo Scientific, catalog no. AB-1453/A).

### qRT-PCR

qRT-PCR was performed following the protocol reported by [65]. Briefly, synthesized cDNA was used as the template and DyNAmo ColorFlash SYBR green qPCR kit (Thermo Scientific, catalog no. F416-L) in a Realplex4 ep gradient Mastercycler (Eppendorf). The annealing temperature was set at 56 °C. The primers used for qRT-PCR analysis are listed in Additional file 9: Table S9. For forward and reverse sequences of few primers, references [65 and 66] were followed. The 16S rRNA gene was used as the housekeeping gene in this study. Relative gene expression was calculated by the threshold cycle (ΔΔCT) method. Genes with a log_2_ fold change of ≥ 1.5 were considered upregulated, and those with a log_2_ fold change of ≤ -1.5 were considered downregulated. Experiments were carried out in biological triplicates with statistical analyses (*t*-test) to validate the fold change in expression of the genes. P < 0.05 was considered statistically significant.

## Supplementary Information

**Additional file 1: Table S1.** Socio-Demographic details of patients with acute gastroenteritis.(n=221) admitted in SCB Medical college, Cuttack, Odisha, India and KIMS, Bhubaneswar, Odisha, India.

**Additional file 2: Table S2.** *S. enterica* Typhimurium ms202 showing resistance to four antibiotic groups. Antibiotic groups are shown yellow highlighted as mentioned in VTEK AST Card (AST N280, Biomereux)

**Additional file 3:** **Table S3.** Multi locus Sequence Typing of the genes in *S. enterica *Typhimurium ms202

**Additional file 4: Table S4.** Details of Phages identified in *S. enterica* Typhimurium ms202. A total of 9 different regions and each region containing different numbers of CDS are shown.

**Additional file 5:** **Table S5.** The interaction networks and the co-expression details of the SPI-2 cluster proteins in *S. enterica* Typhimurium ms202

**Additional file 6: Table S6.** The interaction networks and the co-expression details of the SPI-1 cluster proteins in *S. enterica* Typhimurium ms202

**Additional file 7:** **Table S7.** The whole-genome annotation for virulent genes for *S. enterica* Typhimurium ms202. Genome – *Salmonella enterica* subsp. enterica serovar Typhimurium ms202; Genome ID - 90371.4806, Accession - 90371.4806.con.0001, Annotation – PATRIC, Feature Type – CDS.

**Additional file 8:** **Table S8.** The whole-genome annotation for drug target specific genes for *S. enterica* Typhimurium ms202. Genome – Salmonella enterica subsp. enterica serovar Typhimurium MDR; Genome ID - 90371.4806, Accession - 90371.4806.con.0001, Annotation – PATRIC, Feature Type – CDS

**Additional file 9: Table S9.** List of primers used for qRT-PCR

## Data Availability

Not applicable.
