# Supplementary material for: Genome analysis and virulence gene expression profile of a multi drug resistant Salmonella enterica serovar Typhimurium ms202
Source: Gut Pathog. 2022 Jun 28;14:28. doi: 10.1186/s13099-022-00498-w (PMC9237969; doi:10.1186/s13099-022-00498-w)
Supplement: Supplementary file 1 — Additional file 1: Table S1. Socio-Demographic details of patients with acute gastroenteritis.(n=221) admitted in SCB Medical college, Cuttack, Odisha, India and KIMS, Bhubaneswar, Odisha, India. [file 13099_2022_498_MOESM1_ESM.pdf]

Table S1 – Socio-Demographic details of patients with acute gastroenteritis (n=221) admitted in SCB Medical college, Cuttack, Odisha, India and KIMS, Bhubaneswar, Odisha, India.

**Parameters**	**Numbers**	**Percentages of total**
Male	140	63.3
Female	81	37.7
**Age (In years)**		
1-18 (Paediatric population)	47	21.2
19-40	93	42.3
41-60	53	24.1
61-80	28	12.4
**Religion**		
Hindu	148	66.9
Muslim	65	29.6
Christian	8	3.5
**Place of residence**		
Urban	90	40.6
Rural	131	59.4
**Education**		
Under high school level	70	31.7
Above high school level	129	58.4
Illiterate	22	9.9
**Source of drinking water**		
Use of wells	36	16.1
Use of tube wells	72	32.6
Water tap at home	113	51.3
