# Supplementary material for: Genome analysis and virulence gene expression profile of a multi drug resistant Salmonella enterica serovar Typhimurium ms202
Source: Gut Pathog. 2022 Jun 28;14:28. doi: 10.1186/s13099-022-00498-w (PMC9237969; doi:10.1186/s13099-022-00498-w)
Supplement: Supplementary file 2 — Additional file 2: Table S2. S. enterica Typhimurium ms202 showing resistance to four antibiotic groups. Antibiotic groups are shown yellow highlighted as mentioned in VTEK AST Card (AST N280, Biomereux) [file 13099_2022_498_MOESM2_ESM.pdf]

Table S2 – *S. enterica* Typhimurium ms202 showing resistance to four antibiotic groups. Antibiotic groups are shown yellow highlighted as mentioned in VTEK AST Card (AST N280, Biomereux).

AST N280 (Lower to Higher drug)

Aminopencillins	
Ampicillin	
Beta lactamase inhibitor combination	
Amoxicillin/Clav.acid	
Cefoperazone/Sulbactam	
Piperacillin/Tazobactam	
Cephalosporin 2nd generation	R
Cefuroxime	
Cephalosporin 3rd generation	
Ceftriaxone	
Cephalosporin 4th generation	
Cefepime	
Quinolone	R
Nalidixic-Acid	
Fluoroquinolones	
Ciprofloxacin	
Folate Pathway Antagonist	
Trimethoprim/Sulfamethoxazole	
Nitrofuran	
Nitrofurantoin	
Aminoglycosides	R
Amikacin	
Gentamicin	
Tetracycline	R
Tigecycline	
Carbapenems	
Ertapenem	
Imipenem	
Meropenem	
Polymyxins	
Colistin	

R - Resistance
