# Supplementary material for: Genome analysis and virulence gene expression profile of a multi drug resistant Salmonella enterica serovar Typhimurium ms202
Source: Gut Pathog. 2022 Jun 28;14:28. doi: 10.1186/s13099-022-00498-w (PMC9237969; doi:10.1186/s13099-022-00498-w)
Supplement: Supplementary file 3 — Additional file 3: Table S3. Multi locus Sequence Typing of the genes in S. enterica Typhimurium ms202 [file 13099_2022_498_MOESM3_ESM.pdf]

TABLE S3 Multi locus Sequence Typing of the genes in *S. enterica* Typhimurium ms202.

**Gene[1]**	**Alignment Length[2] = DB Allele Length**
aroc-3	501
dnan-3	501
hemd-7	432
hisd-4	501
pure-3	399
suca-3	501
thra-7	501

[1] Individual gene is added separately to the identified Sequence Type unless all alleles in the input genome match perfectly to a database allele. % Identify = 100, and Gaps = 0 for sequence of all genes.

[2] Length (in bp) of the alignment between the MLST allele in the database (DB) and the corresponding sequence in the input genome.
