# Supplementary material for: Genome analysis and virulence gene expression profile of a multi drug resistant Salmonella enterica serovar Typhimurium ms202
Source: Gut Pathog. 2022 Jun 28;14:28. doi: 10.1186/s13099-022-00498-w (PMC9237969; doi:10.1186/s13099-022-00498-w)
Supplement: Supplementary file 5 — Additional file 5: Table S5. The interaction networks and the co-expression details of the SPI-2 cluster proteins in S. enterica Typhimurium ms202 [file 13099_2022_498_MOESM5_ESM.pdf]

Table S5: The interaction networks and the co-expression details of the SPI2 cluster proteins of *S. enterica* Typhimurium ms202.

node1	node2	node1 accession	node2 accession	node1 annotation	node2 annotation	score
ssrB	ssrA	CY43_07080	CY43_07085	*Type III secretion system regulator; Is phosphorylated by SsrA; is involved in the expression of the virulence genes of Salmonella pathogenicity island-2; Derived by automated computational analysis using gene prediction method: Protein Homology*	*Histidine kinase; Phosphorylates the response regulator SsrB; is involved in the expression of the virulence genes of Salmonella pathogenicity island-2; Derived by automated computational analysis using gene prediction method: Protein Homology*	0.999
ssrB	sseF	CY43_07080	CY43_07145	*Type III secretion system regulator; Is phosphorylated by SsrA; is involved in the expression of the virulence genes of Salmonella pathogenicity island-2; Derived by automated computational analysis using gene prediction method: Protein Homology*	*Pathogenicity island 2 effector protein SseF; With SseG is involved in the aggregation of the host endosomes; Derived by automated computational analysis using gene prediction method: Protein Homology*	0.576
ssrB	sseD	CY43_07080	CY43_07130	*Type III secretion system regulator; Is phosphorylated by SsrA; is involved in the expression of the virulence genes of Salmonella pathogenicity island-2; Derived by automated computational analysis using gene prediction method: Protein Homology*	*SPI-2 type III secretion system translocon protein SseD; May be involved in the translocation of effector proteins into the host cell; Derived by automated computational analysis using gene prediction method: Protein Homology*	0.400
ssrB	sseB	CY43_07080	CY43_07115	*Type III secretion system regulator; Is phosphorylated by SsrA; is involved in the expression of the virulence genes of Salmonella pathogenicity island-2; Derived by automated computational analysis using gene prediction method: Protein Homology*	*SPI-2 type III secretion system translocon protein SseB; Necessary for the correct localization of SseC and SseD on the bacterial cell surface; Derived by automated computational analysis using gene prediction method: Protein Homology*	0.777
ssrB	sscB	CY43_07080	CY43_07140	*Type III secretion system regulator; Is phosphorylated by SsrA; is involved in the expression of the virulence genes of Salmonella pathogenicity island-2; Derived by automated computational analysis using gene prediction method: Protein Homology*	*Salmonella pathogenicity island 2 protein; member of a type III secretion system involved in the survival and replication of Salmonella in a host cell; chaperone for SseF; required for the efficient replication of Salmonella in host macrophages; Derived by automated computational analysis using gene prediction method: Protein Homology*	0.743
ssrB	sscA	CY43_07080	CY43_07120	*Type III secretion system regulator; Is phosphorylated by SsrA; is involved in the*	*CesD/SycD/LcrH family type III secretion system chaperone; May be involved in the*	0.804

				expression of the virulence genes of Salmonella pathogenicity island-2; Derived by automated computational analysis using gene prediction method: Protein Homology	*translocation of effector proteins into the host cell; Derived by automated computational analysis using gene prediction method: Protein Homology*	
ssrB	ssaV	CY43_07080	CY43_07195	*Type III secretion system regulator; Is phosphorylated by SsrA; is involved in the expression of the virulence genes of Salmonella pathogenicity island-2; Derived by automated computational analysis using gene prediction method: Protein Homology*	*SPI-2 type III secretion system apparatus protein SsaV; With SsaC forms part of a protein export system across the inner and outer cell membranes; part of the Salmonella pathogenicity island 2; part of the type III secretion system; Derived by automated computational analysis using gene prediction method: Protein Homology*	0.474
ssrB	ssaU	CY43_07080	CY43_07235	*Type III secretion system regulator; Is phosphorylated by SsrA; is involved in the expression of the virulence genes of Salmonella pathogenicity island-2; Derived by automated computational analysis using gene prediction method: Protein Homology*	*Member of a type III secretion system which is part of a pathogenicity island in Salmonella, Yersinia and pathogenic Escherichia coli; Derived by automated computational analysis using gene prediction method: Protein Homology*	0.519
ssrB	ssaS	CY43_07080	CY43_07225	*Type III secretion system regulator; Is phosphorylated by SsrA; is involved in the expression of the virulence genes of Salmonella pathogenicity island-2; Derived by automated computational analysis using gene prediction method: Protein Homology*	*SPI-2 type III secretion system apparatus protein SsaS; Derived by automated computational analysis using gene prediction method: Protein Homology*	0.493
ssrB	ssaR	CY43_07080	CY43_07220	*Type III secretion system regulator; Is phosphorylated by SsrA; is involved in the expression of the virulence genes of Salmonella pathogenicity island-2; Derived by automated computational analysis using gene prediction method: Protein Homology*	*SPI-2 type III secretion system export apparatus protein SsaR; Part of a set of proteins involved in the infection of eukaryotic cells; in plant pathogens involved in the hypersensitivity response; Derived by automated computational analysis using gene prediction method: Protein Homology*	0.779
ssrB	ssaQ	CY43_07080	CY43_07215	*Type III secretion system regulator; Is phosphorylated by SsrA; is involved in the expression of the virulence genes of Salmonella pathogenicity island-2; Derived by automated computational analysis using gene prediction method: Protein Homology*	*SPI-2 type III secretion system apparatus protein SsaQ; Derived by automated computational analysis using gene prediction method: Protein Homology*	0.687

ssrB	ssaN	CY43_07080	CY43_07200	*Type III secretion system regulator; Is phosphorylated by SsrA; is involved in the expression of the virulence genes of Salmonella pathogenicity island-2; Derived by automated computational analysis using gene prediction method: Protein Homology*	*EscN/YscN/HrcN family type III secretion system ATPase; Derived by automated computational analysis using gene prediction method: Protein Homology*	0.558
ssrB	ssaG	CY43_07080	CY43_07155	*Type III secretion system regulator; Is phosphorylated by SsrA; is involved in the expression of the virulence genes of Salmonella pathogenicity island-2; Derived by automated computational analysis using gene prediction method: Protein Homology*	*EscF/YscF/HrpA family type III secretion system needle major subunit; Salmonella pathogenicity island 2 protein; member of a type III secretion system involved in the survival and replication of Salmonella in a host cell; Derived by automated computational analysis using gene prediction method: Protein Homology*	0.919
ssrB	ssaE	CY43_07080	CY43_07105	*Type III secretion system regulator; Is phosphorylated by SsrA; is involved in the expression of the virulence genes of Salmonella pathogenicity island-2; Derived by automated computational analysis using gene prediction method: Protein Homology*	*Salmonella pathogenicity island 2 protein; member of a type III secretion system involved in the survival and replication of Salmonella in a host cell; involved in the secretion of SseB; Derived by automated computational analysis using gene prediction method: Protein Homology*	0.674
ssrB	ssaD	CY43_07080	CY43_07100	*Type III secretion system regulator; Is phosphorylated by SsrA; is involved in the expression of the virulence genes of Salmonella pathogenicity island-2; Derived by automated computational analysis using gene prediction method: Protein Homology*	*Salmonella pathogenicity island 2 protein; member of a type III secretion system involved in the survival and replication of Salmonella in a host cell; Derived by automated computational analysis using gene prediction method: Protein Homology*	0.717
ssrB	spiC	CY43_07080	CY43_07090	*Type III secretion system regulator; Is phosphorylated by SsrA; is involved in the expression of the virulence genes of Salmonella pathogenicity island-2; Derived by automated computational analysis using gene prediction method: Protein Homology*	*SPI-2 type III secretion system protein SpiC; Involved in macrophage infection; inhibits phagosome-lysosome fusion and cellular trafficking; Derived by automated computational analysis using gene prediction method: Protein Homology*	0.856
ssrB	spiA	CY43_07080	CY43_07095	*Type III secretion system regulator; Is phosphorylated by SsrA; is involved in the expression of the virulence genes of Salmonella pathogenicity island-2; Derived by automated*	*SPI-2 type III secretion system protein SpiA; Derived by automated computational analysis using gene prediction method: Protein Homology*	0.806

				computational analysis using gene prediction method: Protein Homology		
ssrB	DD95_20700	CY43_07080	CY43_07230	*Type III secretion system regulator; Is phosphorylated by SsrA; is involved in the expression of the virulence genes of Salmonella pathogenicity island-2; Derived by automated computational analysis using gene prediction method: Protein Homology*	*Salmonella pathogenicity island 2 protein; member of a type III secretion system involved in the survival and replication of Salmonella in a host cell; Derived by automated computational analysis using gene prediction method: Protein Homology*	0.588
ssrA	ssrB	CY43_07085	CY43_07080	*Histidine kinase; Phosphorylates the response regulator SsrB; is involved in the expression of the virulence genes of Salmonella pathogenicity island-2; Derived by automated computational analysis using gene prediction method: Protein Homology*	*Type III secretion system regulator; Is phosphorylated by SsrA; is involved in the expression of the virulence genes of Salmonella pathogenicity island-2; Derived by automated computational analysis using gene prediction method: Protein Homology*	0.999
ssrA	sseF	CY43_07085	CY43_07145	*Histidine kinase; Phosphorylates the response regulator SsrB; is involved in the expression of the virulence genes of Salmonella pathogenicity island-2; Derived by automated computational analysis using gene prediction method: Protein Homology*	*Pathogenicity island 2 effector protein SseF; With SseG is involved in the aggregation of the host endosomes; Derived by automated computational analysis using gene prediction method: Protein Homology*	0.510
ssrA	sseE	CY43_07085	CY43_07135	*Histidine kinase; Phosphorylates the response regulator SsrB; is involved in the expression of the virulence genes of Salmonella pathogenicity island-2; Derived by automated computational analysis using gene prediction method: Protein Homology*	*Pathogenicity island 2 effector protein SseE; Derived by automated computational analysis using gene prediction method: Protein Homology*	0.630
ssrA	sseD	CY43_07085	CY43_07130	*Histidine kinase; Phosphorylates the response regulator SsrB; is involved in the expression of the virulence genes of Salmonella pathogenicity island-2; Derived by automated computational analysis using gene prediction method: Protein Homology*	*SPI-2 type III secretion system translocon protein SseD; May be involved in the translocation of effector proteins into the host cell; Derived by automated computational analysis using gene prediction method: Protein Homology*	0.579
ssrA	sseB	CY43_07085	CY43_07115	*Histidine kinase; Phosphorylates the response regulator SsrB; is involved in the expression of the virulence genes of Salmonella pathogenicity island-2; Derived by automated computational analysis using gene prediction method: Protein Homology*	*SPI-2 type III secretion system translocon protein SseB; Necessary for the correct localization of SseC and SseD on the bacterial cell surface; Derived by automated computational analysis using gene prediction method: Protein Homology*	0.841

ssrA	sscB	CY43_07085	CY43_07140	*Histidine kinase; Phosphorylates the response regulator SsrB; is involved in the expression of the virulence genes of Salmonella pathogenicity island-2; Derived by automated computational analysis using gene prediction method: Protein Homology*	*Salmonella pathogenicity island 2 protein; member of a type III secretion system involved in the survival and replication of Salmonella in a host cell; chaperone for SseF; required for the efficient replication of Salmonella in host macrophages; Derived by automated computational analysis using gene prediction method: Protein Homology*	0.924
ssrA	sscA	CY43_07085	CY43_07120	*Histidine kinase; Phosphorylates the response regulator SsrB; is involved in the expression of the virulence genes of Salmonella pathogenicity island-2; Derived by automated computational analysis using gene prediction method: Protein Homology*	*CesD/SycD/LcrH family type III secretion system chaperone; May be involved in the translocation of effector proteins into the host cell; Derived by automated computational analysis using gene prediction method: Protein Homology*	0.924
ssrA	ssaV	CY43_07085	CY43_07195	*Histidine kinase; Phosphorylates the response regulator SsrB; is involved in the expression of the virulence genes of Salmonella pathogenicity island-2; Derived by automated computational analysis using gene prediction method: Protein Homology*	*SPI-2 type III secretion system apparatus protein SsaV; With SsaC forms part of a protein export system across the inner and outer cell membranes; part of the Salmonella pathogenicity island 2; part of the type III secretion system; Derived by automated computational analysis using gene prediction method: Protein Homology*	0.469
ssrA	ssaU	CY43_07085	CY43_07235	*Histidine kinase; Phosphorylates the response regulator SsrB; is involved in the expression of the virulence genes of Salmonella pathogenicity island-2; Derived by automated computational analysis using gene prediction method: Protein Homology*	*Member of a type III secretion system which is part of a pathogenicity island in Salmonella, Yersinia and pathogenic Escherichia coli; Derived by automated computational analysis using gene prediction method: Protein Homology*	0.698
ssrA	ssaS	CY43_07085	CY43_07225	*Histidine kinase; Phosphorylates the response regulator SsrB; is involved in the expression of the virulence genes of Salmonella pathogenicity island-2; Derived by automated computational analysis using gene prediction method: Protein Homology*	*SPI-2 type III secretion system apparatus protein SsaS; Derived by automated computational analysis using gene prediction method: Protein Homology*	0.439
ssrA	ssaR	CY43_07085	CY43_07220	*Histidine kinase; Phosphorylates the response regulator SsrB; is involved in the expression of the virulence genes of Salmonella pathogenicity island-2; Derived by automated computational*	*SPI-2 type III secretion system export apparatus protein SsaR; Part of a set of proteins involved in the infection of eukaryotic cells; in plant pathogens involved in the hypersensitivity*	0.747

				analysis using gene prediction method: Protein Homology	*response; Derived by automated computational analysis using gene prediction method: Protein Homology*	
ssrA	ssaQ	CY43_07085	CY43_07215	*Histidine kinase; Phosphorylates the response regulator SsrB; is involved in the expression of the virulence genes of Salmonella pathogenicity island-2; Derived by automated computational analysis using gene prediction method: Protein Homology*	*SPI-2 type III secretion system apparatus protein SsaQ; Derived by automated computational analysis using gene prediction method: Protein Homology*	0.889
ssrA	ssaN	CY43_07085	CY43_07200	*Histidine kinase; Phosphorylates the response regulator SsrB; is involved in the expression of the virulence genes of Salmonella pathogenicity island-2; Derived by automated computational analysis using gene prediction method: Protein Homology*	*EscN/YscN/HrcN family type III secretion system ATPase; Derived by automated computational analysis using gene prediction method: Protein Homology*	0.699
ssrA	ssaI	CY43_07085	CY43_07165	*Histidine kinase; Phosphorylates the response regulator SsrB; is involved in the expression of the virulence genes of Salmonella pathogenicity island-2; Derived by automated computational analysis using gene prediction method: Protein Homology*	*Salmonella pathogenicity island 2 protein; member of a type III secretion system involved in the survival and replication of Salmonella in a host cell; Derived by automated computational analysis using gene prediction method: Protein Homology*	0.556
ssrA	ssaG	CY43_07085	CY43_07155	*Histidine kinase; Phosphorylates the response regulator SsrB; is involved in the expression of the virulence genes of Salmonella pathogenicity island-2; Derived by automated computational analysis using gene prediction method: Protein Homology*	*EscF/YscF/HrpA family type III secretion system needle major subunit; Salmonella pathogenicity island 2 protein; member of a type III secretion system involved in the survival and replication of Salmonella in a host cell; Derived by automated computational analysis using gene prediction method: Protein Homology*	0.903
ssrA	ssaE	CY43_07085	CY43_07105	*Histidine kinase; Phosphorylates the response regulator SsrB; is involved in the expression of the virulence genes of Salmonella pathogenicity island-2; Derived by automated computational analysis using gene prediction method: Protein Homology*	*Salmonella pathogenicity island 2 protein; member of a type III secretion system involved in the survival and replication of Salmonella in a host cell; involved in the secretion of SseB; Derived by automated computational analysis using gene prediction method: Protein Homology*	0.690
ssrA	ssaD	CY43_07085	CY43_07100	*Histidine kinase; Phosphorylates the response regulator SsrB; is involved in the expression of*	*Salmonella pathogenicity island 2 protein; member of a type III secretion system involved*	0.923

				the virulence genes of Salmonella pathogenicity island-2; Derived by automated computational analysis using gene prediction method: Protein Homology	*in the survival and replication of Salmonella in a host cell; Derived by automated computational analysis using gene prediction method: Protein Homology*	
ssrA	spiC	CY43_07085	CY43_07090	*Histidine kinase; Phosphorylates the response regulator SsrB; is involved in the expression of the virulence genes of Salmonella pathogenicity island-2; Derived by automated computational analysis using gene prediction method: Protein Homology*	*SPI-2 type III secretion system protein SpiC; Involved in macrophage infection; inhibits phagosome-lysosome fusion and cellular trafficking; Derived by automated computational analysis using gene prediction method: Protein Homology*	0.902
ssrA	spiA	CY43_07085	CY43_07095	*Histidine kinase; Phosphorylates the response regulator SsrB; is involved in the expression of the virulence genes of Salmonella pathogenicity island-2; Derived by automated computational analysis using gene prediction method: Protein Homology*	*SPI-2 type III secretion system protein SpiA; Derived by automated computational analysis using gene prediction method: Protein Homology*	0.914
ssrA	DD95_20875	CY43_07085	CY43_07050	*Histidine kinase; Phosphorylates the response regulator SsrB; is involved in the expression of the virulence genes of Salmonella pathogenicity island-2; Derived by automated computational analysis using gene prediction method: Protein Homology*	*Histidine kinase; Derived by automated computational analysis using gene prediction method: Protein Homology*	0.798
ssrA	DD95_20700	CY43_07085	CY43_07230	*Histidine kinase; Phosphorylates the response regulator SsrB; is involved in the expression of the virulence genes of Salmonella pathogenicity island-2; Derived by automated computational analysis using gene prediction method: Protein Homology*	*Salmonella pathogenicity island 2 protein; member of a type III secretion system involved in the survival and replication of Salmonella in a host cell; Derived by automated computational analysis using gene prediction method: Protein Homology*	0.703
sseF	ssrB	CY43_07145	CY43_07080	*Pathogenicity island 2 effector protein SseF; With SseG is involved in the aggregation of the host endosomes; Derived by automated computational analysis using gene prediction method: Protein Homology*	*Type III secretion system regulator; Is phosphorylated by SsrA; is involved in the expression of the virulence genes of Salmonella pathogenicity island-2; Derived by automated computational analysis using gene prediction method: Protein Homology*	0.576
sseF	ssrA	CY43_07145	CY43_07085	*Pathogenicity island 2 effector protein SseF; With SseG is involved in the aggregation of the host endosomes; Derived by automated*	*Histidine kinase; Phosphorylates the response regulator SsrB; is involved in the expression of the virulence genes of Salmonella pathogenicity island-2; Derived by automated computational*	0.510

				computational analysis using gene prediction method: Protein Homology	analysis using gene prediction method: Protein Homology	
sseF	sseE	CY43_07145	CY43_07135	*Pathogenicity island 2 effector protein SseF; With SseG is involved in the aggregation of the host endosomes; Derived by automated computational analysis using gene prediction method: Protein Homology*	*Pathogenicity island 2 effector protein SseE; Derived by automated computational analysis using gene prediction method: Protein Homology*	0.828
sseF	sseD	CY43_07145	CY43_07130	*Pathogenicity island 2 effector protein SseF; With SseG is involved in the aggregation of the host endosomes; Derived by automated computational analysis using gene prediction method: Protein Homology*	*SPI-2 type III secretion system translocon protein SseD; May be involved in the translocation of effector proteins into the host cell; Derived by automated computational analysis using gene prediction method: Protein Homology*	0.966
sseF	sseB	CY43_07145	CY43_07115	*Pathogenicity island 2 effector protein SseF; With SseG is involved in the aggregation of the host endosomes; Derived by automated computational analysis using gene prediction method: Protein Homology*	*SPI-2 type III secretion system translocon protein SseB; Necessary for the correct localization of SseC and SseD on the bacterial cell surface; Derived by automated computational analysis using gene prediction method: Protein Homology*	0.886
sseF	sscB	CY43_07145	CY43_07140	*Pathogenicity island 2 effector protein SseF; With SseG is involved in the aggregation of the host endosomes; Derived by automated computational analysis using gene prediction method: Protein Homology*	*Salmonella pathogenicity island 2 protein; member of a type III secretion system involved in the survival and replication of Salmonella in a host cell; chaperone for SseF; required for the efficient replication of Salmonella in host macrophages; Derived by automated computational analysis using gene prediction method: Protein Homology*	0.994
sseF	sscA	CY43_07145	CY43_07120	*Pathogenicity island 2 effector protein SseF; With SseG is involved in the aggregation of the host endosomes; Derived by automated computational analysis using gene prediction method: Protein Homology*	*CesD/SycD/LcrH family type III secretion system chaperone; May be involved in the translocation of effector proteins into the host cell; Derived by automated computational analysis using gene prediction method: Protein Homology*	0.855
sseF	ssaV	CY43_07145	CY43_07195	*Pathogenicity island 2 effector protein SseF; With SseG is involved in the aggregation of the host endosomes; Derived by automated computational analysis using gene prediction method: Protein Homology*	*SPI-2 type III secretion system apparatus protein SsaV; With SsaC forms part of a protein export system across the inner and outer cell membranes; part of the Salmonella pathogenicity island 2; part of the type III*	0.608

					secretion system; Derived by automated computational analysis using gene prediction method: Protein Homology	
sseF	ssaU	CY43_07145	CY43_07235	*Pathogenicity island 2 effector protein SseF; With SseG is involved in the aggregation of the host endosomes; Derived by automated computational analysis using gene prediction method: Protein Homology*	*Member of a type III secretion system which is part of a pathogenicity island in Salmonella, Yersinia and pathogenic Escherichia coli; Derived by automated computational analysis using gene prediction method: Protein Homology*	0.671
sseF	ssaS	CY43_07145	CY43_07225	*Pathogenicity island 2 effector protein SseF; With SseG is involved in the aggregation of the host endosomes; Derived by automated computational analysis using gene prediction method: Protein Homology*	*SPI-2 type III secretion system apparatus protein SsaS; Derived by automated computational analysis using gene prediction method: Protein Homology*	0.791
sseF	ssaR	CY43_07145	CY43_07220	*Pathogenicity island 2 effector protein SseF; With SseG is involved in the aggregation of the host endosomes; Derived by automated computational analysis using gene prediction method: Protein Homology*	*SPI-2 type III secretion system export apparatus protein SsaR; Part of a set of proteins involved in the infection of eukaryotic cells; in plant pathogens involved in the hypersensitivity response; Derived by automated computational analysis using gene prediction method: Protein Homology*	0.587
sseF	ssaQ	CY43_07145	CY43_07215	*Pathogenicity island 2 effector protein SseF; With SseG is involved in the aggregation of the host endosomes; Derived by automated computational analysis using gene prediction method: Protein Homology*	*SPI-2 type III secretion system apparatus protein SsaQ; Derived by automated computational analysis using gene prediction method: Protein Homology*	0.730
sseF	ssaP	CY43_07145	CY43_07210	*Pathogenicity island 2 effector protein SseF; With SseG is involved in the aggregation of the host endosomes; Derived by automated computational analysis using gene prediction method: Protein Homology*	*Salmonella pathogenicity island 2 protein; member of a type III secretion system involved in the survival and replication of Salmonella in a host cell; Derived by automated computational analysis using gene prediction method: Protein Homology*	0.758
sseF	ssaO	CY43_07145	CY43_07205	*Pathogenicity island 2 effector protein SseF; With SseG is involved in the aggregation of the host endosomes; Derived by automated computational analysis using gene prediction method: Protein Homology*	*Salmonella pathogenicity island 2 protein; member of a type III secretion system involved in the survival and replication of Salmonella in a host cell; Derived by automated computational analysis using gene prediction method: Protein Homology*	0.726

sseF	ssaN	CY43_07145	CY43_07200	*Pathogenicity island 2 effector protein SseF; With SseG is involved in the aggregation of the host endosomes; Derived by automated computational analysis using gene prediction method: Protein Homology*	*EscN/YscN/HrcN family type III secretion system ATPase; Derived by automated computational analysis using gene prediction method: Protein Homology*	0.689
sseF	ssaI	CY43_07145	CY43_07165	*Pathogenicity island 2 effector protein SseF; With SseG is involved in the aggregation of the host endosomes; Derived by automated computational analysis using gene prediction method: Protein Homology*	*Salmonella pathogenicity island 2 protein; member of a type III secretion system involved in the survival and replication of Salmonella in a host cell; Derived by automated computational analysis using gene prediction method: Protein Homology*	0.894
sseF	ssaG	CY43_07145	CY43_07155	*Pathogenicity island 2 effector protein SseF; With SseG is involved in the aggregation of the host endosomes; Derived by automated computational analysis using gene prediction method: Protein Homology*	*EscF/YscF/HrpA family type III secretion system needle major subunit; Salmonella pathogenicity island 2 protein; member of a type III secretion system involved in the survival and replication of Salmonella in a host cell; Derived by automated computational analysis using gene prediction method: Protein Homology*	0.715
sseF	ssaE	CY43_07145	CY43_07105	*Pathogenicity island 2 effector protein SseF; With SseG is involved in the aggregation of the host endosomes; Derived by automated computational analysis using gene prediction method: Protein Homology*	*Salmonella pathogenicity island 2 protein; member of a type III secretion system involved in the survival and replication of Salmonella in a host cell; involved in the secretion of SseB; Derived by automated computational analysis using gene prediction method: Protein Homology*	0.576
sseF	ssaD	CY43_07145	CY43_07100	*Pathogenicity island 2 effector protein SseF; With SseG is involved in the aggregation of the host endosomes; Derived by automated computational analysis using gene prediction method: Protein Homology*	*Salmonella pathogenicity island 2 protein; member of a type III secretion system involved in the survival and replication of Salmonella in a host cell; Derived by automated computational analysis using gene prediction method: Protein Homology*	0.628
sseF	spiC	CY43_07145	CY43_07090	*Pathogenicity island 2 effector protein SseF; With SseG is involved in the aggregation of the host endosomes; Derived by automated computational analysis using gene prediction method: Protein Homology*	*SPI-2 type III secretion system protein SpiC; Involved in macrophage infection; inhibits phagosome-lysosome fusion and cellular trafficking; Derived by automated computational analysis using gene prediction method: Protein Homology*	0.803

sseF	spiA	CY43_07145	CY43_07095	*Pathogenicity island 2 effector protein SseF; With SseG is involved in the aggregation of the host endosomes; Derived by automated computational analysis using gene prediction method: Protein Homology*	*SPI-2 type III secretion system protein SpiA; Derived by automated computational analysis using gene prediction method: Protein Homology*	0.628
sseF	DD95_20700	CY43_07145	CY43_07230	*Pathogenicity island 2 effector protein SseF; With SseG is involved in the aggregation of the host endosomes; Derived by automated computational analysis using gene prediction method: Protein Homology*	*Salmonella pathogenicity island 2 protein; member of a type III secretion system involved in the survival and replication of Salmonella in a host cell; Derived by automated computational analysis using gene prediction method: Protein Homology*	0.544
sseE	ssrA	CY43_07135	CY43_07085	*Pathogenicity island 2 effector protein SseE; Derived by automated computational analysis using gene prediction method: Protein Homology*	*Histidine kinase; Phosphorylates the response regulator SsrB; is involved in the expression of the virulence genes of Salmonella pathogenicity island-2; Derived by automated computational analysis using gene prediction method: Protein Homology*	0.630
sseE	sseF	CY43_07135	CY43_07145	*Pathogenicity island 2 effector protein SseE; Derived by automated computational analysis using gene prediction method: Protein Homology*	*Pathogenicity island 2 effector protein SseF; With SseG is involved in the aggregation of the host endosomes; Derived by automated computational analysis using gene prediction method: Protein Homology*	0.828
sseE	sseD	CY43_07135	CY43_07130	*Pathogenicity island 2 effector protein SseE; Derived by automated computational analysis using gene prediction method: Protein Homology*	*SPI-2 type III secretion system translocon protein SseD; May be involved in the translocation of effector proteins into the host cell; Derived by automated computational analysis using gene prediction method: Protein Homology*	0.950
sseE	sseB	CY43_07135	CY43_07115	*Pathogenicity island 2 effector protein SseE; Derived by automated computational analysis using gene prediction method: Protein Homology*	*SPI-2 type III secretion system translocon protein SseB; Necessary for the correct localization of SseC and SseD on the bacterial cell surface; Derived by automated computational analysis using gene prediction method: Protein Homology*	0.957
sseE	sscB	CY43_07135	CY43_07140	*Pathogenicity island 2 effector protein SseE; Derived by automated computational analysis using gene prediction method: Protein Homology*	*Salmonella pathogenicity island 2 protein; member of a type III secretion system involved in the survival and replication of Salmonella in a host cell; chaperone for SseF; required for the*	0.933

					efficient replication of Salmonella in host macrophages; Derived by automated computational analysis using gene prediction method: Protein Homology	
sseE	sscA	CY43_07135	CY43_07120	*Pathogenicity island 2 effector protein SseE; Derived by automated computational analysis using gene prediction method: Protein Homology*	*CesD/SycD/LcrH family type III secretion system chaperone; May be involved in the translocation of effector proteins into the host cell; Derived by automated computational analysis using gene prediction method: Protein Homology*	0.951
sseE	ssaV	CY43_07135	CY43_07195	*Pathogenicity island 2 effector protein SseE; Derived by automated computational analysis using gene prediction method: Protein Homology*	*SPI-2 type III secretion system apparatus protein SsaV; With SsaC forms part of a protein export system across the inner and outer cell membranes; part of the Salmonella pathogenicity island 2; part of the type III secretion system; Derived by automated computational analysis using gene prediction method: Protein Homology*	0.592
sseE	ssaU	CY43_07135	CY43_07235	*Pathogenicity island 2 effector protein SseE; Derived by automated computational analysis using gene prediction method: Protein Homology*	*Member of a type III secretion system which is part of a pathogenicity island in Salmonella, Yersinia and pathogenic Escherichia coli; Derived by automated computational analysis using gene prediction method: Protein Homology*	0.682
sseE	ssaS	CY43_07135	CY43_07225	*Pathogenicity island 2 effector protein SseE; Derived by automated computational analysis using gene prediction method: Protein Homology*	*SPI-2 type III secretion system apparatus protein SsaS; Derived by automated computational analysis using gene prediction method: Protein Homology*	0.729
sseE	ssaR	CY43_07135	CY43_07220	*Pathogenicity island 2 effector protein SseE; Derived by automated computational analysis using gene prediction method: Protein Homology*	*SPI-2 type III secretion system export apparatus protein SsaR; Part of a set of proteins involved in the infection of eukaryotic cells; in plant pathogens involved in the hypersensitivity response; Derived by automated computational analysis using gene prediction method: Protein Homology*	0.838
sseE	ssaQ	CY43_07135	CY43_07215	*Pathogenicity island 2 effector protein SseE; Derived by automated computational analysis*	*SPI-2 type III secretion system apparatus protein SsaQ; Derived by automated*	0.871

				using gene prediction method: Protein Homology	*computational analysis using gene prediction method: Protein Homology*	
sseE	ssaP	CY43_07135	CY43_07210	*Pathogenicity island 2 effector protein SseE; Derived by automated computational analysis using gene prediction method: Protein Homology*	*Salmonella pathogenicity island 2 protein; member of a type III secretion system involved in the survival and replication of Salmonella in a host cell; Derived by automated computational analysis using gene prediction method: Protein Homology*	0.837
sseE	ssaO	CY43_07135	CY43_07205	*Pathogenicity island 2 effector protein SseE; Derived by automated computational analysis using gene prediction method: Protein Homology*	*Salmonella pathogenicity island 2 protein; member of a type III secretion system involved in the survival and replication of Salmonella in a host cell; Derived by automated computational analysis using gene prediction method: Protein Homology*	0.804
sseE	ssaN	CY43_07135	CY43_07200	*Pathogenicity island 2 effector protein SseE; Derived by automated computational analysis using gene prediction method: Protein Homology*	*EscN/YscN/HrcN family type III secretion system ATPase; Derived by automated computational analysis using gene prediction method: Protein Homology*	0.669
sseE	ssaI	CY43_07135	CY43_07165	*Pathogenicity island 2 effector protein SseE; Derived by automated computational analysis using gene prediction method: Protein Homology*	*Salmonella pathogenicity island 2 protein; member of a type III secretion system involved in the survival and replication of Salmonella in a host cell; Derived by automated computational analysis using gene prediction method: Protein Homology*	0.716
sseE	ssaG	CY43_07135	CY43_07155	*Pathogenicity island 2 effector protein SseE; Derived by automated computational analysis using gene prediction method: Protein Homology*	*EscF/YscF/HrpA family type III secretion system needle major subunit; Salmonella pathogenicity island 2 protein; member of a type III secretion system involved in the survival and replication of Salmonella in a host cell; Derived by automated computational analysis using gene prediction method: Protein Homology*	0.656
sseE	ssaE	CY43_07135	CY43_07105	*Pathogenicity island 2 effector protein SseE; Derived by automated computational analysis using gene prediction method: Protein Homology*	*Salmonella pathogenicity island 2 protein; member of a type III secretion system involved in the survival and replication of Salmonella in a host cell; involved in the secretion of SseB; Derived by automated computational analysis*	0.843

					using gene prediction method: Protein Homology	
sseE	ssaD	CY43_07135	CY43_07100	*Pathogenicity island 2 effector protein SseE; Derived by automated computational analysis using gene prediction method: Protein Homology*	*Salmonella pathogenicity island 2 protein; member of a type III secretion system involved in the survival and replication of Salmonella in a host cell; Derived by automated computational analysis using gene prediction method: Protein Homology*	0.884
sseE	spiC	CY43_07135	CY43_07090	*Pathogenicity island 2 effector protein SseE; Derived by automated computational analysis using gene prediction method: Protein Homology*	*SPI-2 type III secretion system protein SpiC; Involved in macrophage infection; inhibits phagosome-lysosome fusion and cellular trafficking; Derived by automated computational analysis using gene prediction method: Protein Homology*	0.844
sseE	spiA	CY43_07135	CY43_07095	*Pathogenicity island 2 effector protein SseE; Derived by automated computational analysis using gene prediction method: Protein Homology*	*SPI-2 type III secretion system protein SpiA; Derived by automated computational analysis using gene prediction method: Protein Homology*	0.738
sseE	DD95_20700	CY43_07135	CY43_07230	*Pathogenicity island 2 effector protein SseE; Derived by automated computational analysis using gene prediction method: Protein Homology*	*Salmonella pathogenicity island 2 protein; member of a type III secretion system involved in the survival and replication of Salmonella in a host cell; Derived by automated computational analysis using gene prediction method: Protein Homology*	0.556
sseD	ssrB	CY43_07130	CY43_07080	*SPI-2 type III secretion system translocon protein SseD; May be involved in the translocation of effector proteins into the host cell; Derived by automated computational analysis using gene prediction method: Protein Homology*	*Type III secretion system regulator; Is phosphorylated by SsrA; is involved in the expression of the virulence genes of Salmonella pathogenicity island-2; Derived by automated computational analysis using gene prediction method: Protein Homology*	0.400
sseD	ssrA	CY43_07130	CY43_07085	*SPI-2 type III secretion system translocon protein SseD; May be involved in the translocation of effector proteins into the host cell; Derived by automated computational analysis using gene prediction method: Protein Homology*	*Histidine kinase; Phosphorylates the response regulator SsrB; is involved in the expression of the virulence genes of Salmonella pathogenicity island-2; Derived by automated computational analysis using gene prediction method: Protein Homology*	0.579
sseD	sseF	CY43_07130	CY43_07145	*SPI-2 type III secretion system translocon protein SseD; May be involved in the*	*Pathogenicity island 2 effector protein SseF; With SseG is involved in the aggregation of the*	0.966

				translocation of effector proteins into the host cell; Derived by automated computational analysis using gene prediction method: Protein Homology	*host endosomes; Derived by automated computational analysis using gene prediction method: Protein Homology*	
sseD	sseE	CY43_07130	CY43_07135	*SPI-2 type III secretion system translocon protein SseD; May be involved in the translocation of effector proteins into the host cell; Derived by automated computational analysis using gene prediction method: Protein Homology*	*Pathogenicity island 2 effector protein SseE; Derived by automated computational analysis using gene prediction method: Protein Homology*	0.950
sseD	sseB	CY43_07130	CY43_07115	*SPI-2 type III secretion system translocon protein SseD; May be involved in the translocation of effector proteins into the host cell; Derived by automated computational analysis using gene prediction method: Protein Homology*	*SPI-2 type III secretion system translocon protein SseB; Necessary for the correct localization of SseC and SseD on the bacterial cell surface; Derived by automated computational analysis using gene prediction method: Protein Homology*	0.987
sseD	sscB	CY43_07130	CY43_07140	*SPI-2 type III secretion system translocon protein SseD; May be involved in the translocation of effector proteins into the host cell; Derived by automated computational analysis using gene prediction method: Protein Homology*	*Salmonella pathogenicity island 2 protein; member of a type III secretion system involved in the survival and replication of Salmonella in a host cell; chaperone for SseF; required for the efficient replication of Salmonella in host macrophages; Derived by automated computational analysis using gene prediction method: Protein Homology*	0.926
sseD	sscA	CY43_07130	CY43_07120	*SPI-2 type III secretion system translocon protein SseD; May be involved in the translocation of effector proteins into the host cell; Derived by automated computational analysis using gene prediction method: Protein Homology*	*CesD/SycD/LcrH family type III secretion system chaperone; May be involved in the translocation of effector proteins into the host cell; Derived by automated computational analysis using gene prediction method: Protein Homology*	0.960
sseD	ssaV	CY43_07130	CY43_07195	*SPI-2 type III secretion system translocon protein SseD; May be involved in the translocation of effector proteins into the host cell; Derived by automated computational analysis using gene prediction method: Protein Homology*	*SPI-2 type III secretion system apparatus protein SsaV; With SsaC forms part of a protein export system across the inner and outer cell membranes; part of the Salmonella pathogenicity island 2; part of the type III secretion system; Derived by automated computational analysis using gene prediction method: Protein Homology*	0.672

sseD	ssaU	CY43_07130	CY43_07235	*SPI-2 type III secretion system translocon protein SseD; May be involved in the translocation of effector proteins into the host cell; Derived by automated computational analysis using gene prediction method: Protein Homology*	*Member of a type III secretion system which is part of a pathogenicity island in Salmonella, Yersinia and pathogenic Escherichia coli; Derived by automated computational analysis using gene prediction method: Protein Homology*	0.676
sseD	ssaS	CY43_07130	CY43_07225	*SPI-2 type III secretion system translocon protein SseD; May be involved in the translocation of effector proteins into the host cell; Derived by automated computational analysis using gene prediction method: Protein Homology*	*SPI-2 type III secretion system apparatus protein SsaS; Derived by automated computational analysis using gene prediction method: Protein Homology*	0.868
sseD	ssaR	CY43_07130	CY43_07220	*SPI-2 type III secretion system translocon protein SseD; May be involved in the translocation of effector proteins into the host cell; Derived by automated computational analysis using gene prediction method: Protein Homology*	*SPI-2 type III secretion system export apparatus protein SsaR; Part of a set of proteins involved in the infection of eukaryotic cells; in plant pathogens involved in the hypersensitivity response; Derived by automated computational analysis using gene prediction method: Protein Homology*	0.599
sseD	ssaQ	CY43_07130	CY43_07215	*SPI-2 type III secretion system translocon protein SseD; May be involved in the translocation of effector proteins into the host cell; Derived by automated computational analysis using gene prediction method: Protein Homology*	*SPI-2 type III secretion system apparatus protein SsaQ; Derived by automated computational analysis using gene prediction method: Protein Homology*	0.738
sseD	ssaP	CY43_07130	CY43_07210	*SPI-2 type III secretion system translocon protein SseD; May be involved in the translocation of effector proteins into the host cell; Derived by automated computational analysis using gene prediction method: Protein Homology*	*Salmonella pathogenicity island 2 protein; member of a type III secretion system involved in the survival and replication of Salmonella in a host cell; Derived by automated computational analysis using gene prediction method: Protein Homology*	0.691
sseD	ssaO	CY43_07130	CY43_07205	*SPI-2 type III secretion system translocon protein SseD; May be involved in the translocation of effector proteins into the host cell; Derived by automated computational analysis using gene prediction method: Protein Homology*	*Salmonella pathogenicity island 2 protein; member of a type III secretion system involved in the survival and replication of Salmonella in a host cell; Derived by automated computational analysis using gene prediction method: Protein Homology*	0.738

sseD	ssaN	CY43_07130	CY43_07200	*SPI-2 type III secretion system translocon protein SseD; May be involved in the translocation of effector proteins into the host cell; Derived by automated computational analysis using gene prediction method: Protein Homology*	*EscN/YscN/HrcN family type III secretion system ATPase; Derived by automated computational analysis using gene prediction method: Protein Homology*	0.726
sseD	ssaI	CY43_07130	CY43_07165	*SPI-2 type III secretion system translocon protein SseD; May be involved in the translocation of effector proteins into the host cell; Derived by automated computational analysis using gene prediction method: Protein Homology*	*Salmonella pathogenicity island 2 protein; member of a type III secretion system involved in the survival and replication of Salmonella in a host cell; Derived by automated computational analysis using gene prediction method: Protein Homology*	0.878
sseD	ssaG	CY43_07130	CY43_07155	*SPI-2 type III secretion system translocon protein SseD; May be involved in the translocation of effector proteins into the host cell; Derived by automated computational analysis using gene prediction method: Protein Homology*	*EscF/YscF/HrpA family type III secretion system needle major subunit; Salmonella pathogenicity island 2 protein; member of a type III secretion system involved in the survival and replication of Salmonella in a host cell; Derived by automated computational analysis using gene prediction method: Protein Homology*	0.888
sseD	ssaE	CY43_07130	CY43_07105	*SPI-2 type III secretion system translocon protein SseD; May be involved in the translocation of effector proteins into the host cell; Derived by automated computational analysis using gene prediction method: Protein Homology*	*Salmonella pathogenicity island 2 protein; member of a type III secretion system involved in the survival and replication of Salmonella in a host cell; involved in the secretion of SseB; Derived by automated computational analysis using gene prediction method: Protein Homology*	0.722
sseD	ssaD	CY43_07130	CY43_07100	*SPI-2 type III secretion system translocon protein SseD; May be involved in the translocation of effector proteins into the host cell; Derived by automated computational analysis using gene prediction method: Protein Homology*	*Salmonella pathogenicity island 2 protein; member of a type III secretion system involved in the survival and replication of Salmonella in a host cell; Derived by automated computational analysis using gene prediction method: Protein Homology*	0.792
sseD	spiC	CY43_07130	CY43_07090	*SPI-2 type III secretion system translocon protein SseD; May be involved in the translocation of effector proteins into the host cell; Derived by automated computational*	*SPI-2 type III secretion system protein SpiC; Involved in macrophage infection; inhibits phagosome-lysosome fusion and cellular trafficking; Derived by automated*	0.863

				analysis using gene prediction method: Protein Homology	*computational analysis using gene prediction method: Protein Homology*	
sseD	spiA	CY43_07130	CY43_07095	*SPI-2 type III secretion system translocon protein SseD; May be involved in the translocation of effector proteins into the host cell; Derived by automated computational analysis using gene prediction method: Protein Homology*	*SPI-2 type III secretion system protein SpiA; Derived by automated computational analysis using gene prediction method: Protein Homology*	0.849
sseD	DD95_20700	CY43_07130	CY43_07230	*SPI-2 type III secretion system translocon protein SseD; May be involved in the translocation of effector proteins into the host cell; Derived by automated computational analysis using gene prediction method: Protein Homology*	*Salmonella pathogenicity island 2 protein; member of a type III secretion system involved in the survival and replication of Salmonella in a host cell; Derived by automated computational analysis using gene prediction method: Protein Homology*	0.656
sseB	ssrB	CY43_07115	CY43_07080	*SPI-2 type III secretion system translocon protein SseB; Necessary for the correct localization of SseC and SseD on the bacterial cell surface; Derived by automated computational analysis using gene prediction method: Protein Homology*	*Type III secretion system regulator; Is phosphorylated by SsrA; is involved in the expression of the virulence genes of Salmonella pathogenicity island-2; Derived by automated computational analysis using gene prediction method: Protein Homology*	0.777
sseB	ssrA	CY43_07115	CY43_07085	*SPI-2 type III secretion system translocon protein SseB; Necessary for the correct localization of SseC and SseD on the bacterial cell surface; Derived by automated computational analysis using gene prediction method: Protein Homology*	*Histidine kinase; Phosphorylates the response regulator SsrB; is involved in the expression of the virulence genes of Salmonella pathogenicity island-2; Derived by automated computational analysis using gene prediction method: Protein Homology*	0.841
sseB	sseF	CY43_07115	CY43_07145	*SPI-2 type III secretion system translocon protein SseB; Necessary for the correct localization of SseC and SseD on the bacterial cell surface; Derived by automated computational analysis using gene prediction method: Protein Homology*	*Pathogenicity island 2 effector protein SseF; With SseG is involved in the aggregation of the host endosomes; Derived by automated computational analysis using gene prediction method: Protein Homology*	0.886
sseB	sseE	CY43_07115	CY43_07135	*SPI-2 type III secretion system translocon protein SseB; Necessary for the correct localization of SseC and SseD on the bacterial cell surface; Derived by automated computational analysis using gene prediction method: Protein Homology*	*Pathogenicity island 2 effector protein SseE; Derived by automated computational analysis using gene prediction method: Protein Homology*	0.957

sseB	sseD	CY43_07115	CY43_07130	*SPI-2 type III secretion system translocon protein SseB; Necessary for the correct localization of SseC and SseD on the bacterial cell surface; Derived by automated computational analysis using gene prediction method: Protein Homology*	*SPI-2 type III secretion system translocon protein SseD; May be involved in the translocation of effector proteins into the host cell; Derived by automated computational analysis using gene prediction method: Protein Homology*	0.987
sseB	sscB	CY43_07115	CY43_07140	*SPI-2 type III secretion system translocon protein SseB; Necessary for the correct localization of SseC and SseD on the bacterial cell surface; Derived by automated computational analysis using gene prediction method: Protein Homology*	*Salmonella pathogenicity island 2 protein; member of a type III secretion system involved in the survival and replication of Salmonella in a host cell; chaperone for SseF; required for the efficient replication of Salmonella in host macrophages; Derived by automated computational analysis using gene prediction method: Protein Homology*	0.857
sseB	sscA	CY43_07115	CY43_07120	*SPI-2 type III secretion system translocon protein SseB; Necessary for the correct localization of SseC and SseD on the bacterial cell surface; Derived by automated computational analysis using gene prediction method: Protein Homology*	*CesD/SycD/LcrH family type III secretion system chaperone; May be involved in the translocation of effector proteins into the host cell; Derived by automated computational analysis using gene prediction method: Protein Homology*	0.976
sseB	ssaV	CY43_07115	CY43_07195	*SPI-2 type III secretion system translocon protein SseB; Necessary for the correct localization of SseC and SseD on the bacterial cell surface; Derived by automated computational analysis using gene prediction method: Protein Homology*	*SPI-2 type III secretion system apparatus protein SsaV; With SsaC forms part of a protein export system across the inner and outer cell membranes; part of the Salmonella pathogenicity island 2; part of the type III secretion system; Derived by automated computational analysis using gene prediction method: Protein Homology*	0.601
sseB	ssaU	CY43_07115	CY43_07235	*SPI-2 type III secretion system translocon protein SseB; Necessary for the correct localization of SseC and SseD on the bacterial cell surface; Derived by automated computational analysis using gene prediction method: Protein Homology*	*Member of a type III secretion system which is part of a pathogenicity island in Salmonella, Yersinia and pathogenic Escherichia coli; Derived by automated computational analysis using gene prediction method: Protein Homology*	0.644
sseB	ssaS	CY43_07115	CY43_07225	*SPI-2 type III secretion system translocon protein SseB; Necessary for the correct localization of SseC and SseD on the bacterial cell surface; Derived by automated*	*SPI-2 type III secretion system apparatus protein SsaS; Derived by automated computational analysis using gene prediction method: Protein Homology*	0.651

				computational analysis using gene prediction method: Protein Homology		
sseB	ssaR	CY43_07115	CY43_07220	*SPI-2 type III secretion system translocon protein SseB; Necessary for the correct localization of SseC and SseD on the bacterial cell surface; Derived by automated computational analysis using gene prediction method: Protein Homology*	*SPI-2 type III secretion system export apparatus protein SsaR; Part of a set of proteins involved in the infection of eukaryotic cells; in plant pathogens involved in the hypersensitivity response; Derived by automated computational analysis using gene prediction method: Protein Homology*	0.633
sseB	ssaQ	CY43_07115	CY43_07215	*SPI-2 type III secretion system translocon protein SseB; Necessary for the correct localization of SseC and SseD on the bacterial cell surface; Derived by automated computational analysis using gene prediction method: Protein Homology*	*SPI-2 type III secretion system apparatus protein SsaQ; Derived by automated computational analysis using gene prediction method: Protein Homology*	0.821
sseB	ssaP	CY43_07115	CY43_07210	*SPI-2 type III secretion system translocon protein SseB; Necessary for the correct localization of SseC and SseD on the bacterial cell surface; Derived by automated computational analysis using gene prediction method: Protein Homology*	*Salmonella pathogenicity island 2 protein; member of a type III secretion system involved in the survival and replication of Salmonella in a host cell; Derived by automated computational analysis using gene prediction method: Protein Homology*	0.660
sseB	ssaO	CY43_07115	CY43_07205	*SPI-2 type III secretion system translocon protein SseB; Necessary for the correct localization of SseC and SseD on the bacterial cell surface; Derived by automated computational analysis using gene prediction method: Protein Homology*	*Salmonella pathogenicity island 2 protein; member of a type III secretion system involved in the survival and replication of Salmonella in a host cell; Derived by automated computational analysis using gene prediction method: Protein Homology*	0.586
sseB	ssaN	CY43_07115	CY43_07200	*SPI-2 type III secretion system translocon protein SseB; Necessary for the correct localization of SseC and SseD on the bacterial cell surface; Derived by automated computational analysis using gene prediction method: Protein Homology*	*EscN/YscN/HrcN family type III secretion system ATPase; Derived by automated computational analysis using gene prediction method: Protein Homology*	0.880
sseB	ssaI	CY43_07115	CY43_07165	*SPI-2 type III secretion system translocon protein SseB; Necessary for the correct localization of SseC and SseD on the bacterial cell surface; Derived by automated*	*Salmonella pathogenicity island 2 protein; member of a type III secretion system involved in the survival and replication of Salmonella in a host cell; Derived by automated*	0.764

				computational analysis using gene prediction method: Protein Homology	*computational analysis using gene prediction method: Protein Homology*	
sseB	ssaG	CY43_07115	CY43_07155	*SPI-2 type III secretion system translocon protein SseB; Necessary for the correct localization of SseC and SseD on the bacterial cell surface; Derived by automated computational analysis using gene prediction method: Protein Homology*	*EscF/YscF/HrpA family type III secretion system needle major subunit; Salmonella pathogenicity island 2 protein; member of a type III secretion system involved in the survival and replication of Salmonella in a host cell; Derived by automated computational analysis using gene prediction method: Protein Homology*	0.878
sseB	ssaE	CY43_07115	CY43_07105	*SPI-2 type III secretion system translocon protein SseB; Necessary for the correct localization of SseC and SseD on the bacterial cell surface; Derived by automated computational analysis using gene prediction method: Protein Homology*	*Salmonella pathogenicity island 2 protein; member of a type III secretion system involved in the survival and replication of Salmonella in a host cell; involved in the secretion of SseB; Derived by automated computational analysis using gene prediction method: Protein Homology*	0.923
sseB	ssaD	CY43_07115	CY43_07100	*SPI-2 type III secretion system translocon protein SseB; Necessary for the correct localization of SseC and SseD on the bacterial cell surface; Derived by automated computational analysis using gene prediction method: Protein Homology*	*Salmonella pathogenicity island 2 protein; member of a type III secretion system involved in the survival and replication of Salmonella in a host cell; Derived by automated computational analysis using gene prediction method: Protein Homology*	0.851
sseB	spiC	CY43_07115	CY43_07090	*SPI-2 type III secretion system translocon protein SseB; Necessary for the correct localization of SseC and SseD on the bacterial cell surface; Derived by automated computational analysis using gene prediction method: Protein Homology*	*SPI-2 type III secretion system protein SpiC; Involved in macrophage infection; inhibits phagosome-lysosome fusion and cellular trafficking; Derived by automated computational analysis using gene prediction method: Protein Homology*	0.877
sseB	spiA	CY43_07115	CY43_07095	*SPI-2 type III secretion system translocon protein SseB; Necessary for the correct localization of SseC and SseD on the bacterial cell surface; Derived by automated computational analysis using gene prediction method: Protein Homology*	*SPI-2 type III secretion system protein SpiA; Derived by automated computational analysis using gene prediction method: Protein Homology*	0.859
sseB	DD95_20700	CY43_07115	CY43_07230	*SPI-2 type III secretion system translocon protein SseB; Necessary for the correct localization of SseC and SseD on the bacterial*	*Salmonella pathogenicity island 2 protein; member of a type III secretion system involved in the survival and replication of Salmonella in*	0.651

				cell surface; Derived by automated computational analysis using gene prediction method: Protein Homology	*a host cell; Derived by automated computational analysis using gene prediction method: Protein Homology*	
sscB	ssrB	CY43_07140	CY43_07080	*Salmonella pathogenicity island 2 protein; member of a type III secretion system involved in the survival and replication of Salmonella in a host cell; chaperone for SseF; required for the efficient replication of Salmonella in host macrophages; Derived by automated computational analysis using gene prediction method: Protein Homology*	*Type III secretion system regulator; Is phosphorylated by SsrA; is involved in the expression of the virulence genes of Salmonella pathogenicity island-2; Derived by automated computational analysis using gene prediction method: Protein Homology*	0.743
sscB	ssrA	CY43_07140	CY43_07085	*Salmonella pathogenicity island 2 protein; member of a type III secretion system involved in the survival and replication of Salmonella in a host cell; chaperone for SseF; required for the efficient replication of Salmonella in host macrophages; Derived by automated computational analysis using gene prediction method: Protein Homology*	*Histidine kinase; Phosphorylates the response regulator SsrB; is involved in the expression of the virulence genes of Salmonella pathogenicity island-2; Derived by automated computational analysis using gene prediction method: Protein Homology*	0.924
sscB	sseF	CY43_07140	CY43_07145	*Salmonella pathogenicity island 2 protein; member of a type III secretion system involved in the survival and replication of Salmonella in a host cell; chaperone for SseF; required for the efficient replication of Salmonella in host macrophages; Derived by automated computational analysis using gene prediction method: Protein Homology*	*Pathogenicity island 2 effector protein SseF; With SseG is involved in the aggregation of the host endosomes; Derived by automated computational analysis using gene prediction method: Protein Homology*	0.994
sscB	sseE	CY43_07140	CY43_07135	*Salmonella pathogenicity island 2 protein; member of a type III secretion system involved in the survival and replication of Salmonella in a host cell; chaperone for SseF; required for the efficient replication of Salmonella in host macrophages; Derived by automated computational analysis using gene prediction method: Protein Homology*	*Pathogenicity island 2 effector protein SseE; Derived by automated computational analysis using gene prediction method: Protein Homology*	0.933
sscB	sseD	CY43_07140	CY43_07130	*Salmonella pathogenicity island 2 protein; member of a type III secretion system involved in the survival and replication of Salmonella in*	*SPI-2 type III secretion system translocon protein SseD; May be involved in the translocation of effector proteins into the host*	0.926

				a host cell; chaperone for SseF; required for the efficient replication of Salmonella in host macrophages; Derived by automated computational analysis using gene prediction method: Protein Homology	*cell; Derived by automated computational analysis using gene prediction method: Protein Homology*	
sscB	sseB	CY43_07140	CY43_07115	*Salmonella pathogenicity island 2 protein; member of a type III secretion system involved in the survival and replication of Salmonella in a host cell; chaperone for SseF; required for the efficient replication of Salmonella in host macrophages; Derived by automated computational analysis using gene prediction method: Protein Homology*	*SPI-2 type III secretion system translocon protein SseB; Necessary for the correct localization of SseC and SseD on the bacterial cell surface; Derived by automated computational analysis using gene prediction method: Protein Homology*	0.857
sscB	sscA	CY43_07140	CY43_07120	*Salmonella pathogenicity island 2 protein; member of a type III secretion system involved in the survival and replication of Salmonella in a host cell; chaperone for SseF; required for the efficient replication of Salmonella in host macrophages; Derived by automated computational analysis using gene prediction method: Protein Homology*	*CesD/SycD/LcrH family type III secretion system chaperone; May be involved in the translocation of effector proteins into the host cell; Derived by automated computational analysis using gene prediction method: Protein Homology*	0.988
sscB	ssaV	CY43_07140	CY43_07195	*Salmonella pathogenicity island 2 protein; member of a type III secretion system involved in the survival and replication of Salmonella in a host cell; chaperone for SseF; required for the efficient replication of Salmonella in host macrophages; Derived by automated computational analysis using gene prediction method: Protein Homology*	*SPI-2 type III secretion system apparatus protein SsaV; With SsaC forms part of a protein export system across the inner and outer cell membranes; part of the Salmonella pathogenicity island 2; part of the type III secretion system; Derived by automated computational analysis using gene prediction method: Protein Homology*	0.773
sscB	ssaU	CY43_07140	CY43_07235	*Salmonella pathogenicity island 2 protein; member of a type III secretion system involved in the survival and replication of Salmonella in a host cell; chaperone for SseF; required for the efficient replication of Salmonella in host macrophages; Derived by automated computational analysis using gene prediction method: Protein Homology*	*Member of a type III secretion system which is part of a pathogenicity island in Salmonella, Yersinia and pathogenic Escherichia coli; Derived by automated computational analysis using gene prediction method: Protein Homology*	0.918

sscB	ssaS	CY43_07140	CY43_07225	*Salmonella pathogenicity island 2 protein; member of a type III secretion system involved in the survival and replication of Salmonella in a host cell; chaperone for SseF; required for the efficient replication of Salmonella in host macrophages; Derived by automated computational analysis using gene prediction method: Protein Homology*	*SPI-2 type III secretion system apparatus protein SsaS; Derived by automated computational analysis using gene prediction method: Protein Homology*	0.839
sscB	ssaR	CY43_07140	CY43_07220	*Salmonella pathogenicity island 2 protein; member of a type III secretion system involved in the survival and replication of Salmonella in a host cell; chaperone for SseF; required for the efficient replication of Salmonella in host macrophages; Derived by automated computational analysis using gene prediction method: Protein Homology*	*SPI-2 type III secretion system export apparatus protein SsaR; Part of a set of proteins involved in the infection of eukaryotic cells; in plant pathogens involved in the hypersensitivity response; Derived by automated computational analysis using gene prediction method: Protein Homology*	0.856
sscB	ssaQ	CY43_07140	CY43_07215	*Salmonella pathogenicity island 2 protein; member of a type III secretion system involved in the survival and replication of Salmonella in a host cell; chaperone for SseF; required for the efficient replication of Salmonella in host macrophages; Derived by automated computational analysis using gene prediction method: Protein Homology*	*SPI-2 type III secretion system apparatus protein SsaQ; Derived by automated computational analysis using gene prediction method: Protein Homology*	0.960
sscB	ssaP	CY43_07140	CY43_07210	*Salmonella pathogenicity island 2 protein; member of a type III secretion system involved in the survival and replication of Salmonella in a host cell; chaperone for SseF; required for the efficient replication of Salmonella in host macrophages; Derived by automated computational analysis using gene prediction method: Protein Homology*	*Salmonella pathogenicity island 2 protein; member of a type III secretion system involved in the survival and replication of Salmonella in a host cell; Derived by automated computational analysis using gene prediction method: Protein Homology*	0.863
sscB	ssaO	CY43_07140	CY43_07205	*Salmonella pathogenicity island 2 protein; member of a type III secretion system involved in the survival and replication of Salmonella in a host cell; chaperone for SseF; required for the efficient replication of Salmonella in host macrophages; Derived by automated*	*Salmonella pathogenicity island 2 protein; member of a type III secretion system involved in the survival and replication of Salmonella in a host cell; Derived by automated computational analysis using gene prediction method: Protein Homology*	0.844

				computational analysis using gene prediction method: Protein Homology		
sscB	ssaN	CY43_07140	CY43_07200	*Salmonella pathogenicity island 2 protein; member of a type III secretion system involved in the survival and replication of Salmonella in a host cell; chaperone for SseF; required for the efficient replication of Salmonella in host macrophages; Derived by automated computational analysis using gene prediction method: Protein Homology*	*EscN/YscN/HrcN family type III secretion system ATPase; Derived by automated computational analysis using gene prediction method: Protein Homology*	0.884
sscB	ssaI	CY43_07140	CY43_07165	*Salmonella pathogenicity island 2 protein; member of a type III secretion system involved in the survival and replication of Salmonella in a host cell; chaperone for SseF; required for the efficient replication of Salmonella in host macrophages; Derived by automated computational analysis using gene prediction method: Protein Homology*	*Salmonella pathogenicity island 2 protein; member of a type III secretion system involved in the survival and replication of Salmonella in a host cell; Derived by automated computational analysis using gene prediction method: Protein Homology*	0.720
sscB	ssaG	CY43_07140	CY43_07155	*Salmonella pathogenicity island 2 protein; member of a type III secretion system involved in the survival and replication of Salmonella in a host cell; chaperone for SseF; required for the efficient replication of Salmonella in host macrophages; Derived by automated computational analysis using gene prediction method: Protein Homology*	*EscF/YscF/HrpA family type III secretion system needle major subunit; Salmonella pathogenicity island 2 protein; member of a type III secretion system involved in the survival and replication of Salmonella in a host cell; Derived by automated computational analysis using gene prediction method: Protein Homology*	0.717
sscB	ssaE	CY43_07140	CY43_07105	*Salmonella pathogenicity island 2 protein; member of a type III secretion system involved in the survival and replication of Salmonella in a host cell; chaperone for SseF; required for the efficient replication of Salmonella in host macrophages; Derived by automated computational analysis using gene prediction method: Protein Homology*	*Salmonella pathogenicity island 2 protein; member of a type III secretion system involved in the survival and replication of Salmonella in a host cell; involved in the secretion of SseB; Derived by automated computational analysis using gene prediction method: Protein Homology*	0.663
sscB	ssaD	CY43_07140	CY43_07100	*Salmonella pathogenicity island 2 protein; member of a type III secretion system involved in the survival and replication of Salmonella in a host cell; chaperone for SseF; required for the*	*Salmonella pathogenicity island 2 protein; member of a type III secretion system involved in the survival and replication of Salmonella in a host cell; Derived by automated*	0.955

				efficient replication of Salmonella in host macrophages; Derived by automated computational analysis using gene prediction method: Protein Homology	*computational analysis using gene prediction method: Protein Homology*	
sscB	spiC	CY43_07140	CY43_07090	*Salmonella pathogenicity island 2 protein; member of a type III secretion system involved in the survival and replication of Salmonella in a host cell; chaperone for SseF; required for the efficient replication of Salmonella in host macrophages; Derived by automated computational analysis using gene prediction method: Protein Homology*	*SPI-2 type III secretion system protein SpiC; Involved in macrophage infection; inhibits phagosome-lysosome fusion and cellular trafficking; Derived by automated computational analysis using gene prediction method: Protein Homology*	0.703
sscB	spiA	CY43_07140	CY43_07095	*Salmonella pathogenicity island 2 protein; member of a type III secretion system involved in the survival and replication of Salmonella in a host cell; chaperone for SseF; required for the efficient replication of Salmonella in host macrophages; Derived by automated computational analysis using gene prediction method: Protein Homology*	*SPI-2 type III secretion system protein SpiA; Derived by automated computational analysis using gene prediction method: Protein Homology*	0.899
sscB	DD95_20700	CY43_07140	CY43_07230	*Salmonella pathogenicity island 2 protein; member of a type III secretion system involved in the survival and replication of Salmonella in a host cell; chaperone for SseF; required for the efficient replication of Salmonella in host macrophages; Derived by automated computational analysis using gene prediction method: Protein Homology*	*Salmonella pathogenicity island 2 protein; member of a type III secretion system involved in the survival and replication of Salmonella in a host cell; Derived by automated computational analysis using gene prediction method: Protein Homology*	0.883
sscA	ssrB	CY43_07120	CY43_07080	*CesD/SycD/LcrH family type III secretion system chaperone; May be involved in the translocation of effector proteins into the host cell; Derived by automated computational analysis using gene prediction method: Protein Homology*	*Type III secretion system regulator; Is phosphorylated by SsrA; is involved in the expression of the virulence genes of Salmonella pathogenicity island-2; Derived by automated computational analysis using gene prediction method: Protein Homology*	0.804
sscA	ssrA	CY43_07120	CY43_07085	*CesD/SycD/LcrH family type III secretion system chaperone; May be involved in the translocation of effector proteins into the host cell; Derived by automated computational*	*Histidine kinase; Phosphorylates the response regulator SsrB; is involved in the expression of the virulence genes of Salmonella pathogenicity island-2; Derived by automated computational*	0.924

				analysis using gene prediction method: Protein Homology	*analysis using gene prediction method: Protein Homology*	
sscA	sseF	CY43_07120	CY43_07145	*CesD/SycD/LcrH family type III secretion system chaperone; May be involved in the translocation of effector proteins into the host cell; Derived by automated computational analysis using gene prediction method: Protein Homology*	*Pathogenicity island 2 effector protein SseF; With SseG is involved in the aggregation of the host endosomes; Derived by automated computational analysis using gene prediction method: Protein Homology*	0.855
sscA	sseE	CY43_07120	CY43_07135	*CesD/SycD/LcrH family type III secretion system chaperone; May be involved in the translocation of effector proteins into the host cell; Derived by automated computational analysis using gene prediction method: Protein Homology*	*Pathogenicity island 2 effector protein SseE; Derived by automated computational analysis using gene prediction method: Protein Homology*	0.951
sscA	sseD	CY43_07120	CY43_07130	*CesD/SycD/LcrH family type III secretion system chaperone; May be involved in the translocation of effector proteins into the host cell; Derived by automated computational analysis using gene prediction method: Protein Homology*	*SPI-2 type III secretion system translocon protein SseD; May be involved in the translocation of effector proteins into the host cell; Derived by automated computational analysis using gene prediction method: Protein Homology*	0.960
sscA	sseB	CY43_07120	CY43_07115	*CesD/SycD/LcrH family type III secretion system chaperone; May be involved in the translocation of effector proteins into the host cell; Derived by automated computational analysis using gene prediction method: Protein Homology*	*SPI-2 type III secretion system translocon protein SseB; Necessary for the correct localization of SseC and SseD on the bacterial cell surface; Derived by automated computational analysis using gene prediction method: Protein Homology*	0.976
sscA	sscB	CY43_07120	CY43_07140	*CesD/SycD/LcrH family type III secretion system chaperone; May be involved in the translocation of effector proteins into the host cell; Derived by automated computational analysis using gene prediction method: Protein Homology*	*Salmonella pathogenicity island 2 protein; member of a type III secretion system involved in the survival and replication of Salmonella in a host cell; chaperone for SseF; required for the efficient replication of Salmonella in host macrophages; Derived by automated computational analysis using gene prediction method: Protein Homology*	0.988
sscA	ssaV	CY43_07120	CY43_07195	*CesD/SycD/LcrH family type III secretion system chaperone; May be involved in the translocation of effector proteins into the host cell; Derived by automated computational*	*SPI-2 type III secretion system apparatus protein SsaV; With SsaC forms part of a protein export system across the inner and outer cell membranes; part of the Salmonella*	0.788

				analysis using gene prediction method: Protein Homology	*pathogenicity island 2; part of the type III secretion system; Derived by automated computational analysis using gene prediction method: Protein Homology*	
sscA	ssaU	CY43_07120	CY43_07235	*CesD/SycD/LcrH family type III secretion system chaperone; May be involved in the translocation of effector proteins into the host cell; Derived by automated computational analysis using gene prediction method: Protein Homology*	*Member of a type III secretion system which is part of a pathogenicity island in Salmonella, Yersinia and pathogenic Escherichia coli; Derived by automated computational analysis using gene prediction method: Protein Homology*	0.921
sscA	ssaS	CY43_07120	CY43_07225	*CesD/SycD/LcrH family type III secretion system chaperone; May be involved in the translocation of effector proteins into the host cell; Derived by automated computational analysis using gene prediction method: Protein Homology*	*SPI-2 type III secretion system apparatus protein SsaS; Derived by automated computational analysis using gene prediction method: Protein Homology*	0.929
sscA	ssaR	CY43_07120	CY43_07220	*CesD/SycD/LcrH family type III secretion system chaperone; May be involved in the translocation of effector proteins into the host cell; Derived by automated computational analysis using gene prediction method: Protein Homology*	*SPI-2 type III secretion system export apparatus protein SsaR; Part of a set of proteins involved in the infection of eukaryotic cells; in plant pathogens involved in the hypersensitivity response; Derived by automated computational analysis using gene prediction method: Protein Homology*	0.888
sscA	ssaQ	CY43_07120	CY43_07215	*CesD/SycD/LcrH family type III secretion system chaperone; May be involved in the translocation of effector proteins into the host cell; Derived by automated computational analysis using gene prediction method: Protein Homology*	*SPI-2 type III secretion system apparatus protein SsaQ; Derived by automated computational analysis using gene prediction method: Protein Homology*	0.965
sscA	ssaP	CY43_07120	CY43_07210	*CesD/SycD/LcrH family type III secretion system chaperone; May be involved in the translocation of effector proteins into the host cell; Derived by automated computational analysis using gene prediction method: Protein Homology*	*Salmonella pathogenicity island 2 protein; member of a type III secretion system involved in the survival and replication of Salmonella in a host cell; Derived by automated computational analysis using gene prediction method: Protein Homology*	0.821
sscA	ssaO	CY43_07120	CY43_07205	*CesD/SycD/LcrH family type III secretion system chaperone; May be involved in the translocation of effector proteins into the host*	*Salmonella pathogenicity island 2 protein; member of a type III secretion system involved in the survival and replication of Salmonella in*	0.821

				cell; Derived by automated computational analysis using gene prediction method: Protein Homology	*a host cell; Derived by automated computational analysis using gene prediction method: Protein Homology*	
sscA	ssaN	CY43_07120	CY43_07200	*CesD/SycD/LcrH family type III secretion system chaperone; May be involved in the translocation of effector proteins into the host cell; Derived by automated computational analysis using gene prediction method: Protein Homology*	*EscN/YscN/HrcN family type III secretion system ATPase; Derived by automated computational analysis using gene prediction method: Protein Homology*	0.926
sscA	ssaI	CY43_07120	CY43_07165	*CesD/SycD/LcrH family type III secretion system chaperone; May be involved in the translocation of effector proteins into the host cell; Derived by automated computational analysis using gene prediction method: Protein Homology*	*Salmonella pathogenicity island 2 protein; member of a type III secretion system involved in the survival and replication of Salmonella in a host cell; Derived by automated computational analysis using gene prediction method: Protein Homology*	0.728
sscA	ssaG	CY43_07120	CY43_07155	*CesD/SycD/LcrH family type III secretion system chaperone; May be involved in the translocation of effector proteins into the host cell; Derived by automated computational analysis using gene prediction method: Protein Homology*	*EscF/YscF/HrpA family type III secretion system needle major subunit; Salmonella pathogenicity island 2 protein; member of a type III secretion system involved in the survival and replication of Salmonella in a host cell; Derived by automated computational analysis using gene prediction method: Protein Homology*	0.862
sscA	ssaE	CY43_07120	CY43_07105	*CesD/SycD/LcrH family type III secretion system chaperone; May be involved in the translocation of effector proteins into the host cell; Derived by automated computational analysis using gene prediction method: Protein Homology*	*Salmonella pathogenicity island 2 protein; member of a type III secretion system involved in the survival and replication of Salmonella in a host cell; involved in the secretion of SseB; Derived by automated computational analysis using gene prediction method: Protein Homology*	0.851
sscA	ssaD	CY43_07120	CY43_07100	*CesD/SycD/LcrH family type III secretion system chaperone; May be involved in the translocation of effector proteins into the host cell; Derived by automated computational analysis using gene prediction method: Protein Homology*	*Salmonella pathogenicity island 2 protein; member of a type III secretion system involved in the survival and replication of Salmonella in a host cell; Derived by automated computational analysis using gene prediction method: Protein Homology*	0.975
sscA	spiC	CY43_07120	CY43_07090	*CesD/SycD/LcrH family type III secretion system chaperone; May be involved in the*	*SPI-2 type III secretion system protein SpiC; Involved in macrophage infection; inhibits*	0.876

				translocation of effector proteins into the host cell; Derived by automated computational analysis using gene prediction method: Protein Homology	*phagosome-lysosome fusion and cellular trafficking; Derived by automated computational analysis using gene prediction method: Protein Homology*	
sscA	spiA	CY43_07120	CY43_07095	*CesD/SycD/LcrH family type III secretion system chaperone; May be involved in the translocation of effector proteins into the host cell; Derived by automated computational analysis using gene prediction method: Protein Homology*	*SPI-2 type III secretion system protein SpiA; Derived by automated computational analysis using gene prediction method: Protein Homology*	0.976
sscA	DD95_20700	CY43_07120	CY43_07230	*CesD/SycD/LcrH family type III secretion system chaperone; May be involved in the translocation of effector proteins into the host cell; Derived by automated computational analysis using gene prediction method: Protein Homology*	*Salmonella pathogenicity island 2 protein; member of a type III secretion system involved in the survival and replication of Salmonella in a host cell; Derived by automated computational analysis using gene prediction method: Protein Homology*	0.906
ssaV	ssrB	CY43_07195	CY43_07080	*SPI-2 type III secretion system apparatus protein SsaV; With SsaC forms part of a protein export system across the inner and outer cell membranes; part of the Salmonella pathogenicity island 2; part of the type III secretion system; Derived by automated computational analysis using gene prediction method: Protein Homology*	*Type III secretion system regulator; Is phosphorylated by SsrA; is involved in the expression of the virulence genes of Salmonella pathogenicity island-2; Derived by automated computational analysis using gene prediction method: Protein Homology*	0.474
ssaV	ssrA	CY43_07195	CY43_07085	*SPI-2 type III secretion system apparatus protein SsaV; With SsaC forms part of a protein export system across the inner and outer cell membranes; part of the Salmonella pathogenicity island 2; part of the type III secretion system; Derived by automated computational analysis using gene prediction method: Protein Homology*	*Histidine kinase; Phosphorylates the response regulator SsrB; is involved in the expression of the virulence genes of Salmonella pathogenicity island-2; Derived by automated computational analysis using gene prediction method: Protein Homology*	0.469
ssaV	sseF	CY43_07195	CY43_07145	*SPI-2 type III secretion system apparatus protein SsaV; With SsaC forms part of a protein export system across the inner and outer cell membranes; part of the Salmonella pathogenicity island 2; part of the type III secretion system; Derived by automated*	*Pathogenicity island 2 effector protein SseF; With SseG is involved in the aggregation of the host endosomes; Derived by automated computational analysis using gene prediction method: Protein Homology*	0.608

				computational analysis using gene prediction method: Protein Homology		
ssaV	sseE	CY43_07195	CY43_07135	*SPI-2 type III secretion system apparatus protein SsaV; With SsaC forms part of a protein export system across the inner and outer cell membranes; part of the Salmonella pathogenicity island 2; part of the type III secretion system; Derived by automated computational analysis using gene prediction method: Protein Homology*	*Pathogenicity island 2 effector protein SseE; Derived by automated computational analysis using gene prediction method: Protein Homology*	0.592
ssaV	sseD	CY43_07195	CY43_07130	*SPI-2 type III secretion system apparatus protein SsaV; With SsaC forms part of a protein export system across the inner and outer cell membranes; part of the Salmonella pathogenicity island 2; part of the type III secretion system; Derived by automated computational analysis using gene prediction method: Protein Homology*	*SPI-2 type III secretion system translocon protein SseD; May be involved in the translocation of effector proteins into the host cell; Derived by automated computational analysis using gene prediction method: Protein Homology*	0.672
ssaV	sseB	CY43_07195	CY43_07115	*SPI-2 type III secretion system apparatus protein SsaV; With SsaC forms part of a protein export system across the inner and outer cell membranes; part of the Salmonella pathogenicity island 2; part of the type III secretion system; Derived by automated computational analysis using gene prediction method: Protein Homology*	*SPI-2 type III secretion system translocon protein SseB; Necessary for the correct localization of SseC and SseD on the bacterial cell surface; Derived by automated computational analysis using gene prediction method: Protein Homology*	0.601
ssaV	sscB	CY43_07195	CY43_07140	*SPI-2 type III secretion system apparatus protein SsaV; With SsaC forms part of a protein export system across the inner and outer cell membranes; part of the Salmonella pathogenicity island 2; part of the type III secretion system; Derived by automated computational analysis using gene prediction method: Protein Homology*	*Salmonella pathogenicity island 2 protein; member of a type III secretion system involved in the survival and replication of Salmonella in a host cell; chaperone for SseF; required for the efficient replication of Salmonella in host macrophages; Derived by automated computational analysis using gene prediction method: Protein Homology*	0.773
ssaV	sscA	CY43_07195	CY43_07120	*SPI-2 type III secretion system apparatus protein SsaV; With SsaC forms part of a protein export system across the inner and outer cell membranes; part of the Salmonella*	*CesD/SycD/LcrH family type III secretion system chaperone; May be involved in the translocation of effector proteins into the host cell; Derived by automated computational*	0.788

				pathogenicity island 2; part of the type III secretion system; Derived by automated computational analysis using gene prediction method: Protein Homology	*analysis using gene prediction method: Protein Homology*	
ssaV	ssaU	CY43_07195	CY43_07235	*SPI-2 type III secretion system apparatus protein SsaV; With SsaC forms part of a protein export system across the inner and outer cell membranes; part of the Salmonella pathogenicity island 2; part of the type III secretion system; Derived by automated computational analysis using gene prediction method: Protein Homology*	*Member of a type III secretion system which is part of a pathogenicity island in Salmonella, Yersinia and pathogenic Escherichia coli; Derived by automated computational analysis using gene prediction method: Protein Homology*	0.960
ssaV	ssaS	CY43_07195	CY43_07225	*SPI-2 type III secretion system apparatus protein SsaV; With SsaC forms part of a protein export system across the inner and outer cell membranes; part of the Salmonella pathogenicity island 2; part of the type III secretion system; Derived by automated computational analysis using gene prediction method: Protein Homology*	*SPI-2 type III secretion system apparatus protein SsaS; Derived by automated computational analysis using gene prediction method: Protein Homology*	0.959
ssaV	ssaR	CY43_07195	CY43_07220	*SPI-2 type III secretion system apparatus protein SsaV; With SsaC forms part of a protein export system across the inner and outer cell membranes; part of the Salmonella pathogenicity island 2; part of the type III secretion system; Derived by automated computational analysis using gene prediction method: Protein Homology*	*SPI-2 type III secretion system export apparatus protein SsaR; Part of a set of proteins involved in the infection of eukaryotic cells; in plant pathogens involved in the hypersensitivity response; Derived by automated computational analysis using gene prediction method: Protein Homology*	0.966
ssaV	ssaQ	CY43_07195	CY43_07215	*SPI-2 type III secretion system apparatus protein SsaV; With SsaC forms part of a protein export system across the inner and outer cell membranes; part of the Salmonella pathogenicity island 2; part of the type III secretion system; Derived by automated computational analysis using gene prediction method: Protein Homology*	*SPI-2 type III secretion system apparatus protein SsaQ; Derived by automated computational analysis using gene prediction method: Protein Homology*	0.928
ssaV	ssaP	CY43_07195	CY43_07210	*SPI-2 type III secretion system apparatus protein SsaV; With SsaC forms part of a protein*	*Salmonella pathogenicity island 2 protein; member of a type III secretion system involved*	0.899

				export system across the inner and outer cell membranes; part of the Salmonella pathogenicity island 2; part of the type III secretion system; Derived by automated computational analysis using gene prediction method: Protein Homology	*in the survival and replication of Salmonella in a host cell; Derived by automated computational analysis using gene prediction method: Protein Homology*	
ssaV	ssaO	CY43_07195	CY43_07205	*SPI-2 type III secretion system apparatus protein SsaV; With SsaC forms part of a protein export system across the inner and outer cell membranes; part of the Salmonella pathogenicity island 2; part of the type III secretion system; Derived by automated computational analysis using gene prediction method: Protein Homology*	*Salmonella pathogenicity island 2 protein; member of a type III secretion system involved in the survival and replication of Salmonella in a host cell; Derived by automated computational analysis using gene prediction method: Protein Homology*	0.899
ssaV	ssaN	CY43_07195	CY43_07200	*SPI-2 type III secretion system apparatus protein SsaV; With SsaC forms part of a protein export system across the inner and outer cell membranes; part of the Salmonella pathogenicity island 2; part of the type III secretion system; Derived by automated computational analysis using gene prediction method: Protein Homology*	*EscN/YscN/HrcN family type III secretion system ATPase; Derived by automated computational analysis using gene prediction method: Protein Homology*	0.980
ssaV	ssaI	CY43_07195	CY43_07165	*SPI-2 type III secretion system apparatus protein SsaV; With SsaC forms part of a protein export system across the inner and outer cell membranes; part of the Salmonella pathogenicity island 2; part of the type III secretion system; Derived by automated computational analysis using gene prediction method: Protein Homology*	*Salmonella pathogenicity island 2 protein; member of a type III secretion system involved in the survival and replication of Salmonella in a host cell; Derived by automated computational analysis using gene prediction method: Protein Homology*	0.763
ssaV	ssaG	CY43_07195	CY43_07155	*SPI-2 type III secretion system apparatus protein SsaV; With SsaC forms part of a protein export system across the inner and outer cell membranes; part of the Salmonella pathogenicity island 2; part of the type III secretion system; Derived by automated computational analysis using gene prediction method: Protein Homology*	*EscF/YscF/HrpA family type III secretion system needle major subunit; Salmonella pathogenicity island 2 protein; member of a type III secretion system involved in the survival and replication of Salmonella in a host cell; Derived by automated computational analysis using gene prediction method: Protein Homology*	0.813

ssaV	ssaE	CY43_07195	CY43_07105	*SPI-2 type III secretion system apparatus protein SsaV; With SsaC forms part of a protein export system across the inner and outer cell membranes; part of the Salmonella pathogenicity island 2; part of the type III secretion system; Derived by automated computational analysis using gene prediction method: Protein Homology*	*Salmonella pathogenicity island 2 protein; member of a type III secretion system involved in the survival and replication of Salmonella in a host cell; involved in the secretion of SseB; Derived by automated computational analysis using gene prediction method: Protein Homology*	0.623
ssaV	ssaD	CY43_07195	CY43_07100	*SPI-2 type III secretion system apparatus protein SsaV; With SsaC forms part of a protein export system across the inner and outer cell membranes; part of the Salmonella pathogenicity island 2; part of the type III secretion system; Derived by automated computational analysis using gene prediction method: Protein Homology*	*Salmonella pathogenicity island 2 protein; member of a type III secretion system involved in the survival and replication of Salmonella in a host cell; Derived by automated computational analysis using gene prediction method: Protein Homology*	0.731
ssaV	spiC	CY43_07195	CY43_07090	*SPI-2 type III secretion system apparatus protein SsaV; With SsaC forms part of a protein export system across the inner and outer cell membranes; part of the Salmonella pathogenicity island 2; part of the type III secretion system; Derived by automated computational analysis using gene prediction method: Protein Homology*	*SPI-2 type III secretion system protein SpiC; Involved in macrophage infection; inhibits phagosome-lysosome fusion and cellular trafficking; Derived by automated computational analysis using gene prediction method: Protein Homology*	0.607
ssaV	spiA	CY43_07195	CY43_07095	*SPI-2 type III secretion system apparatus protein SsaV; With SsaC forms part of a protein export system across the inner and outer cell membranes; part of the Salmonella pathogenicity island 2; part of the type III secretion system; Derived by automated computational analysis using gene prediction method: Protein Homology*	*SPI-2 type III secretion system protein SpiA; Derived by automated computational analysis using gene prediction method: Protein Homology*	0.807
ssaV	DD95_20700	CY43_07195	CY43_07230	*SPI-2 type III secretion system apparatus protein SsaV; With SsaC forms part of a protein export system across the inner and outer cell membranes; part of the Salmonella pathogenicity island 2; part of the type III secretion system; Derived by automated*	*Salmonella pathogenicity island 2 protein; member of a type III secretion system involved in the survival and replication of Salmonella in a host cell; Derived by automated computational analysis using gene prediction method: Protein Homology*	0.962

				computational analysis using gene prediction method: Protein Homology		
ssaU	ssrB	CY43_07235	CY43_07080	*Member of a type III secretion system which is part of a pathogenicity island in Salmonella, Yersinia and pathogenic Escherichia coli; Derived by automated computational analysis using gene prediction method: Protein Homology*	*Type III secretion system regulator; Is phosphorylated by SsrA; is involved in the expression of the virulence genes of Salmonella pathogenicity island-2; Derived by automated computational analysis using gene prediction method: Protein Homology*	0.519
ssaU	ssrA	CY43_07235	CY43_07085	*Member of a type III secretion system which is part of a pathogenicity island in Salmonella, Yersinia and pathogenic Escherichia coli; Derived by automated computational analysis using gene prediction method: Protein Homology*	*Histidine kinase; Phosphorylates the response regulator SsrB; is involved in the expression of the virulence genes of Salmonella pathogenicity island-2; Derived by automated computational analysis using gene prediction method: Protein Homology*	0.698
ssaU	sseF	CY43_07235	CY43_07145	*Member of a type III secretion system which is part of a pathogenicity island in Salmonella, Yersinia and pathogenic Escherichia coli; Derived by automated computational analysis using gene prediction method: Protein Homology*	*Pathogenicity island 2 effector protein SseF; With SseG is involved in the aggregation of the host endosomes; Derived by automated computational analysis using gene prediction method: Protein Homology*	0.671
ssaU	sseE	CY43_07235	CY43_07135	*Member of a type III secretion system which is part of a pathogenicity island in Salmonella, Yersinia and pathogenic Escherichia coli; Derived by automated computational analysis using gene prediction method: Protein Homology*	*Pathogenicity island 2 effector protein SseE; Derived by automated computational analysis using gene prediction method: Protein Homology*	0.682
ssaU	sseD	CY43_07235	CY43_07130	*Member of a type III secretion system which is part of a pathogenicity island in Salmonella, Yersinia and pathogenic Escherichia coli; Derived by automated computational analysis using gene prediction method: Protein Homology*	*SPI-2 type III secretion system translocon protein SseD; May be involved in the translocation of effector proteins into the host cell; Derived by automated computational analysis using gene prediction method: Protein Homology*	0.676
ssaU	sseB	CY43_07235	CY43_07115	*Member of a type III secretion system which is part of a pathogenicity island in Salmonella, Yersinia and pathogenic Escherichia coli; Derived by automated computational analysis using gene prediction method: Protein Homology*	*SPI-2 type III secretion system translocon protein SseB; Necessary for the correct localization of SseC and SseD on the bacterial cell surface; Derived by automated computational analysis using gene prediction method: Protein Homology*	0.644

ssaU	sscB	CY43_07235	CY43_07140	*Member of a type III secretion system which is part of a pathogenicity island in Salmonella, Yersinia and pathogenic Escherichia coli; Derived by automated computational analysis using gene prediction method: Protein Homology*	*Salmonella pathogenicity island 2 protein; member of a type III secretion system involved in the survival and replication of Salmonella in a host cell; chaperone for SseF; required for the efficient replication of Salmonella in host macrophages; Derived by automated computational analysis using gene prediction method: Protein Homology*	0.918
ssaU	sscA	CY43_07235	CY43_07120	*Member of a type III secretion system which is part of a pathogenicity island in Salmonella, Yersinia and pathogenic Escherichia coli; Derived by automated computational analysis using gene prediction method: Protein Homology*	*CesD/SycD/LcrH family type III secretion system chaperone; May be involved in the translocation of effector proteins into the host cell; Derived by automated computational analysis using gene prediction method: Protein Homology*	0.921
ssaU	ssaV	CY43_07235	CY43_07195	*Member of a type III secretion system which is part of a pathogenicity island in Salmonella, Yersinia and pathogenic Escherichia coli; Derived by automated computational analysis using gene prediction method: Protein Homology*	*SPI-2 type III secretion system apparatus protein SsaV; With SsaC forms part of a protein export system across the inner and outer cell membranes; part of the Salmonella pathogenicity island 2; part of the type III secretion system; Derived by automated computational analysis using gene prediction method: Protein Homology*	0.960
ssaU	ssaS	CY43_07235	CY43_07225	*Member of a type III secretion system which is part of a pathogenicity island in Salmonella, Yersinia and pathogenic Escherichia coli; Derived by automated computational analysis using gene prediction method: Protein Homology*	*SPI-2 type III secretion system apparatus protein SsaS; Derived by automated computational analysis using gene prediction method: Protein Homology*	0.997
ssaU	ssaR	CY43_07235	CY43_07220	*Member of a type III secretion system which is part of a pathogenicity island in Salmonella, Yersinia and pathogenic Escherichia coli; Derived by automated computational analysis using gene prediction method: Protein Homology*	*SPI-2 type III secretion system export apparatus protein SsaR; Part of a set of proteins involved in the infection of eukaryotic cells; in plant pathogens involved in the hypersensitivity response; Derived by automated computational analysis using gene prediction method: Protein Homology*	0.994
ssaU	ssaQ	CY43_07235	CY43_07215	*Member of a type III secretion system which is part of a pathogenicity island in Salmonella, Yersinia and pathogenic Escherichia coli;*	*SPI-2 type III secretion system apparatus protein SsaQ; Derived by automated*	0.948

				Derived by automated computational analysis using gene prediction method: Protein Homology	*computational analysis using gene prediction method: Protein Homology*	
ssaU	ssaP	CY43_07235	CY43_07210	*Member of a type III secretion system which is part of a pathogenicity island in Salmonella, Yersinia and pathogenic Escherichia coli; Derived by automated computational analysis using gene prediction method: Protein Homology*	*Salmonella pathogenicity island 2 protein; member of a type III secretion system involved in the survival and replication of Salmonella in a host cell; Derived by automated computational analysis using gene prediction method: Protein Homology*	0.918
ssaU	ssaO	CY43_07235	CY43_07205	*Member of a type III secretion system which is part of a pathogenicity island in Salmonella, Yersinia and pathogenic Escherichia coli; Derived by automated computational analysis using gene prediction method: Protein Homology*	*Salmonella pathogenicity island 2 protein; member of a type III secretion system involved in the survival and replication of Salmonella in a host cell; Derived by automated computational analysis using gene prediction method: Protein Homology*	0.964
ssaU	ssaN	CY43_07235	CY43_07200	*Member of a type III secretion system which is part of a pathogenicity island in Salmonella, Yersinia and pathogenic Escherichia coli; Derived by automated computational analysis using gene prediction method: Protein Homology*	*EscN/YscN/HrcN family type III secretion system ATPase; Derived by automated computational analysis using gene prediction method: Protein Homology*	0.989
ssaU	ssaI	CY43_07235	CY43_07165	*Member of a type III secretion system which is part of a pathogenicity island in Salmonella, Yersinia and pathogenic Escherichia coli; Derived by automated computational analysis using gene prediction method: Protein Homology*	*Salmonella pathogenicity island 2 protein; member of a type III secretion system involved in the survival and replication of Salmonella in a host cell; Derived by automated computational analysis using gene prediction method: Protein Homology*	0.702
ssaU	ssaG	CY43_07235	CY43_07155	*Member of a type III secretion system which is part of a pathogenicity island in Salmonella, Yersinia and pathogenic Escherichia coli; Derived by automated computational analysis using gene prediction method: Protein Homology*	*EscF/YscF/HrpA family type III secretion system needle major subunit; Salmonella pathogenicity island 2 protein; member of a type III secretion system involved in the survival and replication of Salmonella in a host cell; Derived by automated computational analysis using gene prediction method: Protein Homology*	0.790
ssaU	ssaE	CY43_07235	CY43_07105	*Member of a type III secretion system which is part of a pathogenicity island in Salmonella, Yersinia and pathogenic Escherichia coli;*	*Salmonella pathogenicity island 2 protein; member of a type III secretion system involved in the survival and replication of Salmonella in*	0.492

				Derived by automated computational analysis using gene prediction method: Protein Homology	*a host cell; involved in the secretion of SseB; Derived by automated computational analysis using gene prediction method: Protein Homology*	
ssaU	ssaD	CY43_07235	CY43_07100	*Member of a type III secretion system which is part of a pathogenicity island in Salmonella, Yersinia and pathogenic Escherichia coli; Derived by automated computational analysis using gene prediction method: Protein Homology*	*Salmonella pathogenicity island 2 protein; member of a type III secretion system involved in the survival and replication of Salmonella in a host cell; Derived by automated computational analysis using gene prediction method: Protein Homology*	0.902
ssaU	spiC	CY43_07235	CY43_07090	*Member of a type III secretion system which is part of a pathogenicity island in Salmonella, Yersinia and pathogenic Escherichia coli; Derived by automated computational analysis using gene prediction method: Protein Homology*	*SPI-2 type III secretion system protein SpiC; Involved in macrophage infection; inhibits phagosome-lysosome fusion and cellular trafficking; Derived by automated computational analysis using gene prediction method: Protein Homology*	0.662
ssaU	spiA	CY43_07235	CY43_07095	*Member of a type III secretion system which is part of a pathogenicity island in Salmonella, Yersinia and pathogenic Escherichia coli; Derived by automated computational analysis using gene prediction method: Protein Homology*	*SPI-2 type III secretion system protein SpiA; Derived by automated computational analysis using gene prediction method: Protein Homology*	0.815
ssaU	DD95_20700	CY43_07235	CY43_07230	*Member of a type III secretion system which is part of a pathogenicity island in Salmonella, Yersinia and pathogenic Escherichia coli; Derived by automated computational analysis using gene prediction method: Protein Homology*	*Salmonella pathogenicity island 2 protein; member of a type III secretion system involved in the survival and replication of Salmonella in a host cell; Derived by automated computational analysis using gene prediction method: Protein Homology*	0.991
ssaS	ssrB	CY43_07225	CY43_07080	*SPI-2 type III secretion system apparatus protein SsaS; Derived by automated computational analysis using gene prediction method: Protein Homology*	*Type III secretion system regulator; Is phosphorylated by SsrA; is involved in the expression of the virulence genes of Salmonella pathogenicity island-2; Derived by automated computational analysis using gene prediction method: Protein Homology*	0.493
ssaS	ssrA	CY43_07225	CY43_07085	*SPI-2 type III secretion system apparatus protein SsaS; Derived by automated computational analysis using gene prediction method: Protein Homology*	*Histidine kinase; Phosphorylates the response regulator SsrB; is involved in the expression of the virulence genes of Salmonella pathogenicity island-2; Derived by automated computational*	0.439

					analysis using gene prediction method: Protein Homology	
ssaS	sseF	CY43_07225	CY43_07145	*SPI-2 type III secretion system apparatus protein SsaS; Derived by automated computational analysis using gene prediction method: Protein Homology*	*Pathogenicity island 2 effector protein SseF; With SseG is involved in the aggregation of the host endosomes; Derived by automated computational analysis using gene prediction method: Protein Homology*	0.791
ssaS	sseE	CY43_07225	CY43_07135	*SPI-2 type III secretion system apparatus protein SsaS; Derived by automated computational analysis using gene prediction method: Protein Homology*	*Pathogenicity island 2 effector protein SseE; Derived by automated computational analysis using gene prediction method: Protein Homology*	0.729
ssaS	sseD	CY43_07225	CY43_07130	*SPI-2 type III secretion system apparatus protein SsaS; Derived by automated computational analysis using gene prediction method: Protein Homology*	*SPI-2 type III secretion system translocon protein SseD; May be involved in the translocation of effector proteins into the host cell; Derived by automated computational analysis using gene prediction method: Protein Homology*	0.868
ssaS	sseB	CY43_07225	CY43_07115	*SPI-2 type III secretion system apparatus protein SsaS; Derived by automated computational analysis using gene prediction method: Protein Homology*	*SPI-2 type III secretion system translocon protein SseB; Necessary for the correct localization of SseC and SseD on the bacterial cell surface; Derived by automated computational analysis using gene prediction method: Protein Homology*	0.651
ssaS	sscB	CY43_07225	CY43_07140	*SPI-2 type III secretion system apparatus protein SsaS; Derived by automated computational analysis using gene prediction method: Protein Homology*	*Salmonella pathogenicity island 2 protein; member of a type III secretion system involved in the survival and replication of Salmonella in a host cell; chaperone for SseF; required for the efficient replication of Salmonella in host macrophages; Derived by automated computational analysis using gene prediction method: Protein Homology*	0.839
ssaS	sscA	CY43_07225	CY43_07120	*SPI-2 type III secretion system apparatus protein SsaS; Derived by automated computational analysis using gene prediction method: Protein Homology*	*CesD/SycD/LcrH family type III secretion system chaperone; May be involved in the translocation of effector proteins into the host cell; Derived by automated computational analysis using gene prediction method: Protein Homology*	0.929

ssaS	ssaV	CY43_07225	CY43_07195	*SPI-2 type III secretion system apparatus protein SsaS; Derived by automated computational analysis using gene prediction method: Protein Homology*	*SPI-2 type III secretion system apparatus protein SsaV; With SsaC forms part of a protein export system across the inner and outer cell membranes; part of the Salmonella pathogenicity island 2; part of the type III secretion system; Derived by automated computational analysis using gene prediction method: Protein Homology*	0.959
ssaS	ssaU	CY43_07225	CY43_07235	*SPI-2 type III secretion system apparatus protein SsaS; Derived by automated computational analysis using gene prediction method: Protein Homology*	*Member of a type III secretion system which is part of a pathogenicity island in Salmonella, Yersinia and pathogenic Escherichia coli; Derived by automated computational analysis using gene prediction method: Protein Homology*	0.997
ssaS	ssaR	CY43_07225	CY43_07220	*SPI-2 type III secretion system apparatus protein SsaS; Derived by automated computational analysis using gene prediction method: Protein Homology*	*SPI-2 type III secretion system export apparatus protein SsaR; Part of a set of proteins involved in the infection of eukaryotic cells; in plant pathogens involved in the hypersensitivity response; Derived by automated computational analysis using gene prediction method: Protein Homology*	0.996
ssaS	ssaQ	CY43_07225	CY43_07215	*SPI-2 type III secretion system apparatus protein SsaS; Derived by automated computational analysis using gene prediction method: Protein Homology*	*SPI-2 type III secretion system apparatus protein SsaQ; Derived by automated computational analysis using gene prediction method: Protein Homology*	0.939
ssaS	ssaP	CY43_07225	CY43_07210	*SPI-2 type III secretion system apparatus protein SsaS; Derived by automated computational analysis using gene prediction method: Protein Homology*	*Salmonella pathogenicity island 2 protein; member of a type III secretion system involved in the survival and replication of Salmonella in a host cell; Derived by automated computational analysis using gene prediction method: Protein Homology*	0.928
ssaS	ssaO	CY43_07225	CY43_07205	*SPI-2 type III secretion system apparatus protein SsaS; Derived by automated computational analysis using gene prediction method: Protein Homology*	*Salmonella pathogenicity island 2 protein; member of a type III secretion system involved in the survival and replication of Salmonella in a host cell; Derived by automated computational analysis using gene prediction method: Protein Homology*	0.968

ssaS	ssaN	CY43_07225	CY43_07200	*SPI-2 type III secretion system apparatus protein SsaS; Derived by automated computational analysis using gene prediction method: Protein Homology*	*EscN/YscN/HrcN family type III secretion system ATPase; Derived by automated computational analysis using gene prediction method: Protein Homology*	0.986
ssaS	ssaI	CY43_07225	CY43_07165	*SPI-2 type III secretion system apparatus protein SsaS; Derived by automated computational analysis using gene prediction method: Protein Homology*	*Salmonella pathogenicity island 2 protein; member of a type III secretion system involved in the survival and replication of Salmonella in a host cell; Derived by automated computational analysis using gene prediction method: Protein Homology*	0.841
ssaS	ssaG	CY43_07225	CY43_07155	*SPI-2 type III secretion system apparatus protein SsaS; Derived by automated computational analysis using gene prediction method: Protein Homology*	*EscF/YscF/HrpA family type III secretion system needle major subunit; Salmonella pathogenicity island 2 protein; member of a type III secretion system involved in the survival and replication of Salmonella in a host cell; Derived by automated computational analysis using gene prediction method: Protein Homology*	0.735
ssaS	ssaE	CY43_07225	CY43_07105	*SPI-2 type III secretion system apparatus protein SsaS; Derived by automated computational analysis using gene prediction method: Protein Homology*	*Salmonella pathogenicity island 2 protein; member of a type III secretion system involved in the survival and replication of Salmonella in a host cell; involved in the secretion of SseB; Derived by automated computational analysis using gene prediction method: Protein Homology*	0.598
ssaS	ssaD	CY43_07225	CY43_07100	*SPI-2 type III secretion system apparatus protein SsaS; Derived by automated computational analysis using gene prediction method: Protein Homology*	*Salmonella pathogenicity island 2 protein; member of a type III secretion system involved in the survival and replication of Salmonella in a host cell; Derived by automated computational analysis using gene prediction method: Protein Homology*	0.860
ssaS	spiC	CY43_07225	CY43_07090	*SPI-2 type III secretion system apparatus protein SsaS; Derived by automated computational analysis using gene prediction method: Protein Homology*	*SPI-2 type III secretion system protein SpiC; Involved in macrophage infection; inhibits phagosome-lysosome fusion and cellular trafficking; Derived by automated computational analysis using gene prediction method: Protein Homology*	0.691

ssaS	spiA	CY43_07225	CY43_07095	*SPI-2 type III secretion system apparatus protein SsaS; Derived by automated computational analysis using gene prediction method: Protein Homology*	*SPI-2 type III secretion system protein SpiA; Derived by automated computational analysis using gene prediction method: Protein Homology*	0.807
ssaS	DD95_20700	CY43_07225	CY43_07230	*SPI-2 type III secretion system apparatus protein SsaS; Derived by automated computational analysis using gene prediction method: Protein Homology*	*Salmonella pathogenicity island 2 protein; member of a type III secretion system involved in the survival and replication of Salmonella in a host cell; Derived by automated computational analysis using gene prediction method: Protein Homology*	0.983
ssaR	ssrB	CY43_07220	CY43_07080	*SPI-2 type III secretion system export apparatus protein SsaR; Part of a set of proteins involved in the infection of eukaryotic cells; in plant pathogens involved in the hypersensitivity response; Derived by automated computational analysis using gene prediction method: Protein Homology*	*Type III secretion system regulator; Is phosphorylated by SsrA; is involved in the expression of the virulence genes of Salmonella pathogenicity island-2; Derived by automated computational analysis using gene prediction method: Protein Homology*	0.779
ssaR	ssrA	CY43_07220	CY43_07085	*SPI-2 type III secretion system export apparatus protein SsaR; Part of a set of proteins involved in the infection of eukaryotic cells; in plant pathogens involved in the hypersensitivity response; Derived by automated computational analysis using gene prediction method: Protein Homology*	*Histidine kinase; Phosphorylates the response regulator SsrB; is involved in the expression of the virulence genes of Salmonella pathogenicity island-2; Derived by automated computational analysis using gene prediction method: Protein Homology*	0.747
ssaR	sseF	CY43_07220	CY43_07145	*SPI-2 type III secretion system export apparatus protein SsaR; Part of a set of proteins involved in the infection of eukaryotic cells; in plant pathogens involved in the hypersensitivity response; Derived by automated computational analysis using gene prediction method: Protein Homology*	*Pathogenicity island 2 effector protein SseF; With SseG is involved in the aggregation of the host endosomes; Derived by automated computational analysis using gene prediction method: Protein Homology*	0.587
ssaR	sseE	CY43_07220	CY43_07135	*SPI-2 type III secretion system export apparatus protein SsaR; Part of a set of proteins involved in the infection of eukaryotic cells; in plant pathogens involved in the hypersensitivity response; Derived by automated computational analysis using gene prediction method: Protein Homology*	*Pathogenicity island 2 effector protein SseE; Derived by automated computational analysis using gene prediction method: Protein Homology*	0.838

ssaR	sseD	CY43_07220	CY43_07130	*SPI-2 type III secretion system export apparatus protein SsaR; Part of a set of proteins involved in the infection of eukaryotic cells; in plant pathogens involved in the hypersensitivity response; Derived by automated computational analysis using gene prediction method: Protein Homology*	*SPI-2 type III secretion system translocon protein SseD; May be involved in the translocation of effector proteins into the host cell; Derived by automated computational analysis using gene prediction method: Protein Homology*	0.599
ssaR	sseB	CY43_07220	CY43_07115	*SPI-2 type III secretion system export apparatus protein SsaR; Part of a set of proteins involved in the infection of eukaryotic cells; in plant pathogens involved in the hypersensitivity response; Derived by automated computational analysis using gene prediction method: Protein Homology*	*SPI-2 type III secretion system translocon protein SseB; Necessary for the correct localization of SseC and SseD on the bacterial cell surface; Derived by automated computational analysis using gene prediction method: Protein Homology*	0.633
ssaR	sscB	CY43_07220	CY43_07140	*SPI-2 type III secretion system export apparatus protein SsaR; Part of a set of proteins involved in the infection of eukaryotic cells; in plant pathogens involved in the hypersensitivity response; Derived by automated computational analysis using gene prediction method: Protein Homology*	*Salmonella pathogenicity island 2 protein; member of a type III secretion system involved in the survival and replication of Salmonella in a host cell; chaperone for SseF; required for the efficient replication of Salmonella in host macrophages; Derived by automated computational analysis using gene prediction method: Protein Homology*	0.856
ssaR	sscA	CY43_07220	CY43_07120	*SPI-2 type III secretion system export apparatus protein SsaR; Part of a set of proteins involved in the infection of eukaryotic cells; in plant pathogens involved in the hypersensitivity response; Derived by automated computational analysis using gene prediction method: Protein Homology*	*CesD/SycD/LcrH family type III secretion system chaperone; May be involved in the translocation of effector proteins into the host cell; Derived by automated computational analysis using gene prediction method: Protein Homology*	0.888
ssaR	ssaV	CY43_07220	CY43_07195	*SPI-2 type III secretion system export apparatus protein SsaR; Part of a set of proteins involved in the infection of eukaryotic cells; in plant pathogens involved in the hypersensitivity response; Derived by automated computational analysis using gene prediction method: Protein Homology*	*SPI-2 type III secretion system apparatus protein SsaV; With SsaC forms part of a protein export system across the inner and outer cell membranes; part of the Salmonella pathogenicity island 2; part of the type III secretion system; Derived by automated computational analysis using gene prediction method: Protein Homology*	0.966

ssaR	ssaU	CY43_07220	CY43_07235	*SPI-2 type III secretion system export apparatus protein SsaR; Part of a set of proteins involved in the infection of eukaryotic cells; in plant pathogens involved in the hypersensitivity response; Derived by automated computational analysis using gene prediction method: Protein Homology*	*Member of a type III secretion system which is part of a pathogenicity island in Salmonella, Yersinia and pathogenic Escherichia coli; Derived by automated computational analysis using gene prediction method: Protein Homology*	0.994
ssaR	ssaS	CY43_07220	CY43_07225	*SPI-2 type III secretion system export apparatus protein SsaR; Part of a set of proteins involved in the infection of eukaryotic cells; in plant pathogens involved in the hypersensitivity response; Derived by automated computational analysis using gene prediction method: Protein Homology*	*SPI-2 type III secretion system apparatus protein SsaS; Derived by automated computational analysis using gene prediction method: Protein Homology*	0.996
ssaR	ssaQ	CY43_07220	CY43_07215	*SPI-2 type III secretion system export apparatus protein SsaR; Part of a set of proteins involved in the infection of eukaryotic cells; in plant pathogens involved in the hypersensitivity response; Derived by automated computational analysis using gene prediction method: Protein Homology*	*SPI-2 type III secretion system apparatus protein SsaQ; Derived by automated computational analysis using gene prediction method: Protein Homology*	0.935
ssaR	ssaP	CY43_07220	CY43_07210	*SPI-2 type III secretion system export apparatus protein SsaR; Part of a set of proteins involved in the infection of eukaryotic cells; in plant pathogens involved in the hypersensitivity response; Derived by automated computational analysis using gene prediction method: Protein Homology*	*Salmonella pathogenicity island 2 protein; member of a type III secretion system involved in the survival and replication of Salmonella in a host cell; Derived by automated computational analysis using gene prediction method: Protein Homology*	0.922
ssaR	ssaO	CY43_07220	CY43_07205	*SPI-2 type III secretion system export apparatus protein SsaR; Part of a set of proteins involved in the infection of eukaryotic cells; in plant pathogens involved in the hypersensitivity response; Derived by automated computational analysis using gene prediction method: Protein Homology*	*Salmonella pathogenicity island 2 protein; member of a type III secretion system involved in the survival and replication of Salmonella in a host cell; Derived by automated computational analysis using gene prediction method: Protein Homology*	0.922
ssaR	ssaN	CY43_07220	CY43_07200	*SPI-2 type III secretion system export apparatus protein SsaR; Part of a set of proteins involved in the infection of eukaryotic cells; in plant*	*EscN/YscN/HrcN family type III secretion system ATPase; Derived by automated*	0.986

				pathogens involved in the hypersensitivity response; Derived by automated computational analysis using gene prediction method: Protein Homology	*computational analysis using gene prediction method: Protein Homology*	
ssaR	ssaI	CY43_07220	CY43_07165	*SPI-2 type III secretion system export apparatus protein SsaR; Part of a set of proteins involved in the infection of eukaryotic cells; in plant pathogens involved in the hypersensitivity response; Derived by automated computational analysis using gene prediction method: Protein Homology*	*Salmonella pathogenicity island 2 protein; member of a type III secretion system involved in the survival and replication of Salmonella in a host cell; Derived by automated computational analysis using gene prediction method: Protein Homology*	0.751
ssaR	ssaG	CY43_07220	CY43_07155	*SPI-2 type III secretion system export apparatus protein SsaR; Part of a set of proteins involved in the infection of eukaryotic cells; in plant pathogens involved in the hypersensitivity response; Derived by automated computational analysis using gene prediction method: Protein Homology*	*EscF/YscF/HrpA family type III secretion system needle major subunit; Salmonella pathogenicity island 2 protein; member of a type III secretion system involved in the survival and replication of Salmonella in a host cell; Derived by automated computational analysis using gene prediction method: Protein Homology*	0.893
ssaR	ssaE	CY43_07220	CY43_07105	*SPI-2 type III secretion system export apparatus protein SsaR; Part of a set of proteins involved in the infection of eukaryotic cells; in plant pathogens involved in the hypersensitivity response; Derived by automated computational analysis using gene prediction method: Protein Homology*	*Salmonella pathogenicity island 2 protein; member of a type III secretion system involved in the survival and replication of Salmonella in a host cell; involved in the secretion of SseB; Derived by automated computational analysis using gene prediction method: Protein Homology*	0.710
ssaR	ssaD	CY43_07220	CY43_07100	*SPI-2 type III secretion system export apparatus protein SsaR; Part of a set of proteins involved in the infection of eukaryotic cells; in plant pathogens involved in the hypersensitivity response; Derived by automated computational analysis using gene prediction method: Protein Homology*	*Salmonella pathogenicity island 2 protein; member of a type III secretion system involved in the survival and replication of Salmonella in a host cell; Derived by automated computational analysis using gene prediction method: Protein Homology*	0.888
ssaR	spiC	CY43_07220	CY43_07090	*SPI-2 type III secretion system export apparatus protein SsaR; Part of a set of proteins involved in the infection of eukaryotic cells; in plant pathogens involved in the hypersensitivity response; Derived by automated computational*	*SPI-2 type III secretion system protein SpiC; Involved in macrophage infection; inhibits phagosome-lysosome fusion and cellular trafficking; Derived by automated*	0.841

				analysis using gene prediction method: Protein Homology	*computational analysis using gene prediction method: Protein Homology*	
ssaR	spiA	CY43_07220	CY43_07095	*SPI-2 type III secretion system export apparatus protein SsaR; Part of a set of proteins involved in the infection of eukaryotic cells; in plant pathogens involved in the hypersensitivity response; Derived by automated computational analysis using gene prediction method: Protein Homology*	*SPI-2 type III secretion system protein SpiA; Derived by automated computational analysis using gene prediction method: Protein Homology*	0.839
ssaR	DD95_20700	CY43_07220	CY43_07230	*SPI-2 type III secretion system export apparatus protein SsaR; Part of a set of proteins involved in the infection of eukaryotic cells; in plant pathogens involved in the hypersensitivity response; Derived by automated computational analysis using gene prediction method: Protein Homology*	*Salmonella pathogenicity island 2 protein; member of a type III secretion system involved in the survival and replication of Salmonella in a host cell; Derived by automated computational analysis using gene prediction method: Protein Homology*	0.990
ssaQ	ssrB	CY43_07215	CY43_07080	*SPI-2 type III secretion system apparatus protein SsaQ; Derived by automated computational analysis using gene prediction method: Protein Homology*	*Type III secretion system regulator; Is phosphorylated by SsrA; is involved in the expression of the virulence genes of Salmonella pathogenicity island-2; Derived by automated computational analysis using gene prediction method: Protein Homology*	0.687
ssaQ	ssrA	CY43_07215	CY43_07085	*SPI-2 type III secretion system apparatus protein SsaQ; Derived by automated computational analysis using gene prediction method: Protein Homology*	*Histidine kinase; Phosphorylates the response regulator SsrB; is involved in the expression of the virulence genes of Salmonella pathogenicity island-2; Derived by automated computational analysis using gene prediction method: Protein Homology*	0.889
ssaQ	sseF	CY43_07215	CY43_07145	*SPI-2 type III secretion system apparatus protein SsaQ; Derived by automated computational analysis using gene prediction method: Protein Homology*	*Pathogenicity island 2 effector protein SseF; With SseG is involved in the aggregation of the host endosomes; Derived by automated computational analysis using gene prediction method: Protein Homology*	0.730
ssaQ	sseE	CY43_07215	CY43_07135	*SPI-2 type III secretion system apparatus protein SsaQ; Derived by automated computational analysis using gene prediction method: Protein Homology*	*Pathogenicity island 2 effector protein SseE; Derived by automated computational analysis using gene prediction method: Protein Homology*	0.871

ssaQ	sseD	CY43_07215	CY43_07130	*SPI-2 type III secretion system apparatus protein SsaQ; Derived by automated computational analysis using gene prediction method: Protein Homology*	*SPI-2 type III secretion system translocon protein SseD; May be involved in the translocation of effector proteins into the host cell; Derived by automated computational analysis using gene prediction method: Protein Homology*	0.738
ssaQ	sseB	CY43_07215	CY43_07115	*SPI-2 type III secretion system apparatus protein SsaQ; Derived by automated computational analysis using gene prediction method: Protein Homology*	*SPI-2 type III secretion system translocon protein SseB; Necessary for the correct localization of SseC and SseD on the bacterial cell surface; Derived by automated computational analysis using gene prediction method: Protein Homology*	0.821
ssaQ	sscB	CY43_07215	CY43_07140	*SPI-2 type III secretion system apparatus protein SsaQ; Derived by automated computational analysis using gene prediction method: Protein Homology*	*Salmonella pathogenicity island 2 protein; member of a type III secretion system involved in the survival and replication of Salmonella in a host cell; chaperone for SseF; required for the efficient replication of Salmonella in host macrophages; Derived by automated computational analysis using gene prediction method: Protein Homology*	0.960
ssaQ	sscA	CY43_07215	CY43_07120	*SPI-2 type III secretion system apparatus protein SsaQ; Derived by automated computational analysis using gene prediction method: Protein Homology*	*CesD/SycD/LcrH family type III secretion system chaperone; May be involved in the translocation of effector proteins into the host cell; Derived by automated computational analysis using gene prediction method: Protein Homology*	0.965
ssaQ	ssaV	CY43_07215	CY43_07195	*SPI-2 type III secretion system apparatus protein SsaQ; Derived by automated computational analysis using gene prediction method: Protein Homology*	*SPI-2 type III secretion system apparatus protein SsaV; With SsaC forms part of a protein export system across the inner and outer cell membranes; part of the Salmonella pathogenicity island 2; part of the type III secretion system; Derived by automated computational analysis using gene prediction method: Protein Homology*	0.928
ssaQ	ssaU	CY43_07215	CY43_07235	*SPI-2 type III secretion system apparatus protein SsaQ; Derived by automated computational analysis using gene prediction method: Protein Homology*	*Member of a type III secretion system which is part of a pathogenicity island in Salmonella, Yersinia and pathogenic Escherichia coli; Derived by automated computational analysis*	0.948

					using gene prediction method: Protein Homology	
ssaQ	ssaS	CY43_07215	CY43_07225	*SPI-2 type III secretion system apparatus protein SsaQ; Derived by automated computational analysis using gene prediction method: Protein Homology*	*SPI-2 type III secretion system apparatus protein SsaS; Derived by automated computational analysis using gene prediction method: Protein Homology*	0.939
ssaQ	ssaR	CY43_07215	CY43_07220	*SPI-2 type III secretion system apparatus protein SsaQ; Derived by automated computational analysis using gene prediction method: Protein Homology*	*SPI-2 type III secretion system export apparatus protein SsaR; Part of a set of proteins involved in the infection of eukaryotic cells; in plant pathogens involved in the hypersensitivity response; Derived by automated computational analysis using gene prediction method: Protein Homology*	0.935
ssaQ	ssaP	CY43_07215	CY43_07210	*SPI-2 type III secretion system apparatus protein SsaQ; Derived by automated computational analysis using gene prediction method: Protein Homology*	*Salmonella pathogenicity island 2 protein; member of a type III secretion system involved in the survival and replication of Salmonella in a host cell; Derived by automated computational analysis using gene prediction method: Protein Homology*	0.978
ssaQ	ssaO	CY43_07215	CY43_07205	*SPI-2 type III secretion system apparatus protein SsaQ; Derived by automated computational analysis using gene prediction method: Protein Homology*	*Salmonella pathogenicity island 2 protein; member of a type III secretion system involved in the survival and replication of Salmonella in a host cell; Derived by automated computational analysis using gene prediction method: Protein Homology*	0.977
ssaQ	ssaN	CY43_07215	CY43_07200	*SPI-2 type III secretion system apparatus protein SsaQ; Derived by automated computational analysis using gene prediction method: Protein Homology*	*EscN/YscN/HrcN family type III secretion system ATPase; Derived by automated computational analysis using gene prediction method: Protein Homology*	0.979
ssaQ	ssaI	CY43_07215	CY43_07165	*SPI-2 type III secretion system apparatus protein SsaQ; Derived by automated computational analysis using gene prediction method: Protein Homology*	*Salmonella pathogenicity island 2 protein; member of a type III secretion system involved in the survival and replication of Salmonella in a host cell; Derived by automated computational analysis using gene prediction method: Protein Homology*	0.821
ssaQ	ssaG	CY43_07215	CY43_07155	*SPI-2 type III secretion system apparatus protein SsaQ; Derived by automated*	*EscF/YscF/HrpA family type III secretion system needle major subunit; Salmonella pathogenicity island 2 protein; member of a*	0.754

				computational analysis using gene prediction method: Protein Homology	*type III secretion system involved in the survival and replication of Salmonella in a host cell; Derived by automated computational analysis using gene prediction method: Protein Homology*	
ssaQ	ssaE	CY43_07215	CY43_07105	*SPI-2 type III secretion system apparatus protein SsaQ; Derived by automated computational analysis using gene prediction method: Protein Homology*	*Salmonella pathogenicity island 2 protein; member of a type III secretion system involved in the survival and replication of Salmonella in a host cell; involved in the secretion of SseB; Derived by automated computational analysis using gene prediction method: Protein Homology*	0.767
ssaQ	ssaD	CY43_07215	CY43_07100	*SPI-2 type III secretion system apparatus protein SsaQ; Derived by automated computational analysis using gene prediction method: Protein Homology*	*Salmonella pathogenicity island 2 protein; member of a type III secretion system involved in the survival and replication of Salmonella in a host cell; Derived by automated computational analysis using gene prediction method: Protein Homology*	0.964
ssaQ	spiC	CY43_07215	CY43_07090	*SPI-2 type III secretion system apparatus protein SsaQ; Derived by automated computational analysis using gene prediction method: Protein Homology*	*SPI-2 type III secretion system protein SpiC; Involved in macrophage infection; inhibits phagosome-lysosome fusion and cellular trafficking; Derived by automated computational analysis using gene prediction method: Protein Homology*	0.651
ssaQ	spiA	CY43_07215	CY43_07095	*SPI-2 type III secretion system apparatus protein SsaQ; Derived by automated computational analysis using gene prediction method: Protein Homology*	*SPI-2 type III secretion system protein SpiA; Derived by automated computational analysis using gene prediction method: Protein Homology*	0.948
ssaQ	DD95_20700	CY43_07215	CY43_07230	*SPI-2 type III secretion system apparatus protein SsaQ; Derived by automated computational analysis using gene prediction method: Protein Homology*	*Salmonella pathogenicity island 2 protein; member of a type III secretion system involved in the survival and replication of Salmonella in a host cell; Derived by automated computational analysis using gene prediction method: Protein Homology*	0.919
ssaP	sseF	CY43_07210	CY43_07145	*Salmonella pathogenicity island 2 protein; member of a type III secretion system involved in the survival and replication of Salmonella in a host cell; Derived by automated*	*Pathogenicity island 2 effector protein SseF; With SseG is involved in the aggregation of the host endosomes; Derived by automated*	0.758

				computational analysis using gene prediction method: Protein Homology	*computational analysis using gene prediction method: Protein Homology*	
ssaP	sseE	CY43_07210	CY43_07135	*Salmonella pathogenicity island 2 protein; member of a type III secretion system involved in the survival and replication of Salmonella in a host cell; Derived by automated computational analysis using gene prediction method: Protein Homology*	*Pathogenicity island 2 effector protein SseE; Derived by automated computational analysis using gene prediction method: Protein Homology*	0.837
ssaP	sseD	CY43_07210	CY43_07130	*Salmonella pathogenicity island 2 protein; member of a type III secretion system involved in the survival and replication of Salmonella in a host cell; Derived by automated computational analysis using gene prediction method: Protein Homology*	*SPI-2 type III secretion system translocon protein SseD; May be involved in the translocation of effector proteins into the host cell; Derived by automated computational analysis using gene prediction method: Protein Homology*	0.691
ssaP	sseB	CY43_07210	CY43_07115	*Salmonella pathogenicity island 2 protein; member of a type III secretion system involved in the survival and replication of Salmonella in a host cell; Derived by automated computational analysis using gene prediction method: Protein Homology*	*SPI-2 type III secretion system translocon protein SseB; Necessary for the correct localization of SseC and SseD on the bacterial cell surface; Derived by automated computational analysis using gene prediction method: Protein Homology*	0.660
ssaP	sscB	CY43_07210	CY43_07140	*Salmonella pathogenicity island 2 protein; member of a type III secretion system involved in the survival and replication of Salmonella in a host cell; Derived by automated computational analysis using gene prediction method: Protein Homology*	*Salmonella pathogenicity island 2 protein; member of a type III secretion system involved in the survival and replication of Salmonella in a host cell; chaperone for SseF; required for the efficient replication of Salmonella in host macrophages; Derived by automated computational analysis using gene prediction method: Protein Homology*	0.863
ssaP	sscA	CY43_07210	CY43_07120	*Salmonella pathogenicity island 2 protein; member of a type III secretion system involved in the survival and replication of Salmonella in a host cell; Derived by automated computational analysis using gene prediction method: Protein Homology*	*CesD/SycD/LcrH family type III secretion system chaperone; May be involved in the translocation of effector proteins into the host cell; Derived by automated computational analysis using gene prediction method: Protein Homology*	0.821
ssaP	ssaV	CY43_07210	CY43_07195	*Salmonella pathogenicity island 2 protein; member of a type III secretion system involved in the survival and replication of Salmonella in a host cell; Derived by automated*	*SPI-2 type III secretion system apparatus protein SsaV; With SsaC forms part of a protein export system across the inner and outer cell membranes; part of the Salmonella*	0.899

				computational analysis using gene prediction method: Protein Homology	*pathogenicity island 2; part of the type III secretion system; Derived by automated computational analysis using gene prediction method: Protein Homology*	
ssaP	ssaU	CY43_07210	CY43_07235	*Salmonella pathogenicity island 2 protein; member of a type III secretion system involved in the survival and replication of Salmonella in a host cell; Derived by automated computational analysis using gene prediction method: Protein Homology*	*Member of a type III secretion system which is part of a pathogenicity island in Salmonella, Yersinia and pathogenic Escherichia coli; Derived by automated computational analysis using gene prediction method: Protein Homology*	0.918
ssaP	ssaS	CY43_07210	CY43_07225	*Salmonella pathogenicity island 2 protein; member of a type III secretion system involved in the survival and replication of Salmonella in a host cell; Derived by automated computational analysis using gene prediction method: Protein Homology*	*SPI-2 type III secretion system apparatus protein SsaS; Derived by automated computational analysis using gene prediction method: Protein Homology*	0.928
ssaP	ssaR	CY43_07210	CY43_07220	*Salmonella pathogenicity island 2 protein; member of a type III secretion system involved in the survival and replication of Salmonella in a host cell; Derived by automated computational analysis using gene prediction method: Protein Homology*	*SPI-2 type III secretion system export apparatus protein SsaR; Part of a set of proteins involved in the infection of eukaryotic cells; in plant pathogens involved in the hypersensitivity response; Derived by automated computational analysis using gene prediction method: Protein Homology*	0.922
ssaP	ssaQ	CY43_07210	CY43_07215	*Salmonella pathogenicity island 2 protein; member of a type III secretion system involved in the survival and replication of Salmonella in a host cell; Derived by automated computational analysis using gene prediction method: Protein Homology*	*SPI-2 type III secretion system apparatus protein SsaQ; Derived by automated computational analysis using gene prediction method: Protein Homology*	0.978
ssaP	ssaO	CY43_07210	CY43_07205	*Salmonella pathogenicity island 2 protein; member of a type III secretion system involved in the survival and replication of Salmonella in a host cell; Derived by automated computational analysis using gene prediction method: Protein Homology*	*Salmonella pathogenicity island 2 protein; member of a type III secretion system involved in the survival and replication of Salmonella in a host cell; Derived by automated computational analysis using gene prediction method: Protein Homology*	0.983
ssaP	ssaN	CY43_07210	CY43_07200	*Salmonella pathogenicity island 2 protein; member of a type III secretion system involved in the survival and replication of Salmonella in*	*EscN/YscN/HrcN family type III secretion system ATPase; Derived by automated*	0.969

				a host cell; Derived by automated computational analysis using gene prediction method: Protein Homology	*computational analysis using gene prediction method: Protein Homology*	
ssaP	ssaI	CY43_07210	CY43_07165	*Salmonella pathogenicity island 2 protein; member of a type III secretion system involved in the survival and replication of Salmonella in a host cell; Derived by automated computational analysis using gene prediction method: Protein Homology*	*Salmonella pathogenicity island 2 protein; member of a type III secretion system involved in the survival and replication of Salmonella in a host cell; Derived by automated computational analysis using gene prediction method: Protein Homology*	0.757
ssaP	ssaG	CY43_07210	CY43_07155	*Salmonella pathogenicity island 2 protein; member of a type III secretion system involved in the survival and replication of Salmonella in a host cell; Derived by automated computational analysis using gene prediction method: Protein Homology*	*EscF/YscF/HrpA family type III secretion system needle major subunit; Salmonella pathogenicity island 2 protein; member of a type III secretion system involved in the survival and replication of Salmonella in a host cell; Derived by automated computational analysis using gene prediction method: Protein Homology*	0.747
ssaP	ssaE	CY43_07210	CY43_07105	*Salmonella pathogenicity island 2 protein; member of a type III secretion system involved in the survival and replication of Salmonella in a host cell; Derived by automated computational analysis using gene prediction method: Protein Homology*	*Salmonella pathogenicity island 2 protein; member of a type III secretion system involved in the survival and replication of Salmonella in a host cell; involved in the secretion of SseB; Derived by automated computational analysis using gene prediction method: Protein Homology*	0.478
ssaP	ssaD	CY43_07210	CY43_07100	*Salmonella pathogenicity island 2 protein; member of a type III secretion system involved in the survival and replication of Salmonella in a host cell; Derived by automated computational analysis using gene prediction method: Protein Homology*	*Salmonella pathogenicity island 2 protein; member of a type III secretion system involved in the survival and replication of Salmonella in a host cell; Derived by automated computational analysis using gene prediction method: Protein Homology*	0.840
ssaP	spiC	CY43_07210	CY43_07090	*Salmonella pathogenicity island 2 protein; member of a type III secretion system involved in the survival and replication of Salmonella in a host cell; Derived by automated computational analysis using gene prediction method: Protein Homology*	*SPI-2 type III secretion system protein SpiC; Involved in macrophage infection; inhibits phagosome-lysosome fusion and cellular trafficking; Derived by automated computational analysis using gene prediction method: Protein Homology*	0.683
ssaP	spiA	CY43_07210	CY43_07095	*Salmonella pathogenicity island 2 protein; member of a type III secretion system involved*	*SPI-2 type III secretion system protein SpiA; Derived by automated computational analysis*	0.626

				in the survival and replication of Salmonella in a host cell; Derived by automated computational analysis using gene prediction method: Protein Homology	*using gene prediction method: Protein Homology*	
ssaP	DD95_20700	CY43_07210	CY43_07230	*Salmonella pathogenicity island 2 protein; member of a type III secretion system involved in the survival and replication of Salmonella in a host cell; Derived by automated computational analysis using gene prediction method: Protein Homology*	*Salmonella pathogenicity island 2 protein; member of a type III secretion system involved in the survival and replication of Salmonella in a host cell; Derived by automated computational analysis using gene prediction method: Protein Homology*	0.762
ssaO	sseF	CY43_07205	CY43_07145	*Salmonella pathogenicity island 2 protein; member of a type III secretion system involved in the survival and replication of Salmonella in a host cell; Derived by automated computational analysis using gene prediction method: Protein Homology*	*Pathogenicity island 2 effector protein SseF; With SseG is involved in the aggregation of the host endosomes; Derived by automated computational analysis using gene prediction method: Protein Homology*	0.726
ssaO	sseE	CY43_07205	CY43_07135	*Salmonella pathogenicity island 2 protein; member of a type III secretion system involved in the survival and replication of Salmonella in a host cell; Derived by automated computational analysis using gene prediction method: Protein Homology*	*Pathogenicity island 2 effector protein SseE; Derived by automated computational analysis using gene prediction method: Protein Homology*	0.804
ssaO	sseD	CY43_07205	CY43_07130	*Salmonella pathogenicity island 2 protein; member of a type III secretion system involved in the survival and replication of Salmonella in a host cell; Derived by automated computational analysis using gene prediction method: Protein Homology*	*SPI-2 type III secretion system translocon protein SseD; May be involved in the translocation of effector proteins into the host cell; Derived by automated computational analysis using gene prediction method: Protein Homology*	0.738
ssaO	sseB	CY43_07205	CY43_07115	*Salmonella pathogenicity island 2 protein; member of a type III secretion system involved in the survival and replication of Salmonella in a host cell; Derived by automated computational analysis using gene prediction method: Protein Homology*	*SPI-2 type III secretion system translocon protein SseB; Necessary for the correct localization of SseC and SseD on the bacterial cell surface; Derived by automated computational analysis using gene prediction method: Protein Homology*	0.586
ssaO	sscB	CY43_07205	CY43_07140	*Salmonella pathogenicity island 2 protein; member of a type III secretion system involved in the survival and replication of Salmonella in*	*Salmonella pathogenicity island 2 protein; member of a type III secretion system involved in the survival and replication of Salmonella in*	0.844

				a host cell; Derived by automated computational analysis using gene prediction method: Protein Homology	*a host cell; chaperone for SseF; required for the efficient replication of Salmonella in host macrophages; Derived by automated computational analysis using gene prediction method: Protein Homology*	
ssaO	sscA	CY43_07205	CY43_07120	*Salmonella pathogenicity island 2 protein; member of a type III secretion system involved in the survival and replication of Salmonella in a host cell; Derived by automated computational analysis using gene prediction method: Protein Homology*	*CesD/SycD/LcrH family type III secretion system chaperone; May be involved in the translocation of effector proteins into the host cell; Derived by automated computational analysis using gene prediction method: Protein Homology*	0.821
ssaO	ssaV	CY43_07205	CY43_07195	*Salmonella pathogenicity island 2 protein; member of a type III secretion system involved in the survival and replication of Salmonella in a host cell; Derived by automated computational analysis using gene prediction method: Protein Homology*	*SPI-2 type III secretion system apparatus protein SsaV; With SsaC forms part of a protein export system across the inner and outer cell membranes; part of the Salmonella pathogenicity island 2; part of the type III secretion system; Derived by automated computational analysis using gene prediction method: Protein Homology*	0.899
ssaO	ssaU	CY43_07205	CY43_07235	*Salmonella pathogenicity island 2 protein; member of a type III secretion system involved in the survival and replication of Salmonella in a host cell; Derived by automated computational analysis using gene prediction method: Protein Homology*	*Member of a type III secretion system which is part of a pathogenicity island in Salmonella, Yersinia and pathogenic Escherichia coli; Derived by automated computational analysis using gene prediction method: Protein Homology*	0.964
ssaO	ssaS	CY43_07205	CY43_07225	*Salmonella pathogenicity island 2 protein; member of a type III secretion system involved in the survival and replication of Salmonella in a host cell; Derived by automated computational analysis using gene prediction method: Protein Homology*	*SPI-2 type III secretion system apparatus protein SsaS; Derived by automated computational analysis using gene prediction method: Protein Homology*	0.968
ssaO	ssaR	CY43_07205	CY43_07220	*Salmonella pathogenicity island 2 protein; member of a type III secretion system involved in the survival and replication of Salmonella in a host cell; Derived by automated computational analysis using gene prediction method: Protein Homology*	*SPI-2 type III secretion system export apparatus protein SsaR; Part of a set of proteins involved in the infection of eukaryotic cells; in plant pathogens involved in the hypersensitivity response; Derived by automated computational analysis using gene prediction method: Protein Homology*	0.922

ssaO	ssaQ	CY43_07205	CY43_07215	*Salmonella pathogenicity island 2 protein; member of a type III secretion system involved in the survival and replication of Salmonella in a host cell; Derived by automated computational analysis using gene prediction method: Protein Homology*	*SPI-2 type III secretion system apparatus protein SsaQ; Derived by automated computational analysis using gene prediction method: Protein Homology*	0.977
ssaO	ssaP	CY43_07205	CY43_07210	*Salmonella pathogenicity island 2 protein; member of a type III secretion system involved in the survival and replication of Salmonella in a host cell; Derived by automated computational analysis using gene prediction method: Protein Homology*	*Salmonella pathogenicity island 2 protein; member of a type III secretion system involved in the survival and replication of Salmonella in a host cell; Derived by automated computational analysis using gene prediction method: Protein Homology*	0.983
ssaO	ssaN	CY43_07205	CY43_07200	*Salmonella pathogenicity island 2 protein; member of a type III secretion system involved in the survival and replication of Salmonella in a host cell; Derived by automated computational analysis using gene prediction method: Protein Homology*	*EscN/YscN/HrcN family type III secretion system ATPase; Derived by automated computational analysis using gene prediction method: Protein Homology*	0.969
ssaO	ssaI	CY43_07205	CY43_07165	*Salmonella pathogenicity island 2 protein; member of a type III secretion system involved in the survival and replication of Salmonella in a host cell; Derived by automated computational analysis using gene prediction method: Protein Homology*	*Salmonella pathogenicity island 2 protein; member of a type III secretion system involved in the survival and replication of Salmonella in a host cell; Derived by automated computational analysis using gene prediction method: Protein Homology*	0.759
ssaO	ssaG	CY43_07205	CY43_07155	*Salmonella pathogenicity island 2 protein; member of a type III secretion system involved in the survival and replication of Salmonella in a host cell; Derived by automated computational analysis using gene prediction method: Protein Homology*	*EscF/YscF/HrpA family type III secretion system needle major subunit; Salmonella pathogenicity island 2 protein; member of a type III secretion system involved in the survival and replication of Salmonella in a host cell; Derived by automated computational analysis using gene prediction method: Protein Homology*	0.750
ssaO	ssaE	CY43_07205	CY43_07105	*Salmonella pathogenicity island 2 protein; member of a type III secretion system involved in the survival and replication of Salmonella in a host cell; Derived by automated computational analysis using gene prediction method: Protein Homology*	*Salmonella pathogenicity island 2 protein; member of a type III secretion system involved in the survival and replication of Salmonella in a host cell; involved in the secretion of SseB; Derived by automated computational analysis*	0.494

					using gene prediction method: Protein Homology	
ssaO	ssaD	CY43_07205	CY43_07100	*Salmonella pathogenicity island 2 protein; member of a type III secretion system involved in the survival and replication of Salmonella in a host cell; Derived by automated computational analysis using gene prediction method: Protein Homology*	*Salmonella pathogenicity island 2 protein; member of a type III secretion system involved in the survival and replication of Salmonella in a host cell; Derived by automated computational analysis using gene prediction method: Protein Homology*	0.839
ssaO	spiC	CY43_07205	CY43_07090	*Salmonella pathogenicity island 2 protein; member of a type III secretion system involved in the survival and replication of Salmonella in a host cell; Derived by automated computational analysis using gene prediction method: Protein Homology*	*SPI-2 type III secretion system protein SpiC; Involved in macrophage infection; inhibits phagosome-lysosome fusion and cellular trafficking; Derived by automated computational analysis using gene prediction method: Protein Homology*	0.642
ssaO	spiA	CY43_07205	CY43_07095	*Salmonella pathogenicity island 2 protein; member of a type III secretion system involved in the survival and replication of Salmonella in a host cell; Derived by automated computational analysis using gene prediction method: Protein Homology*	*SPI-2 type III secretion system protein SpiA; Derived by automated computational analysis using gene prediction method: Protein Homology*	0.486
ssaO	DD95_20700	CY43_07205	CY43_07230	*Salmonella pathogenicity island 2 protein; member of a type III secretion system involved in the survival and replication of Salmonella in a host cell; Derived by automated computational analysis using gene prediction method: Protein Homology*	*Salmonella pathogenicity island 2 protein; member of a type III secretion system involved in the survival and replication of Salmonella in a host cell; Derived by automated computational analysis using gene prediction method: Protein Homology*	0.763
ssaN	ssrB	CY43_07200	CY43_07080	*EscN/YscN/HrcN family type III secretion system ATPase; Derived by automated computational analysis using gene prediction method: Protein Homology*	*Type III secretion system regulator; Is phosphorylated by SsrA; is involved in the expression of the virulence genes of Salmonella pathogenicity island-2; Derived by automated computational analysis using gene prediction method: Protein Homology*	0.558
ssaN	ssrA	CY43_07200	CY43_07085	*EscN/YscN/HrcN family type III secretion system ATPase; Derived by automated computational analysis using gene prediction method: Protein Homology*	*Histidine kinase; Phosphorylates the response regulator SsrB; is involved in the expression of the virulence genes of Salmonella pathogenicity island-2; Derived by automated computational analysis using gene prediction method: Protein Homology*	0.699

ssaN	sseF	CY43_07200	CY43_07145	*EscN/YscN/HrcN family type III secretion system ATPase; Derived by automated computational analysis using gene prediction method: Protein Homology*	*Pathogenicity island 2 effector protein SseF; With SseG is involved in the aggregation of the host endosomes; Derived by automated computational analysis using gene prediction method: Protein Homology*	0.689
ssaN	sseE	CY43_07200	CY43_07135	*EscN/YscN/HrcN family type III secretion system ATPase; Derived by automated computational analysis using gene prediction method: Protein Homology*	*Pathogenicity island 2 effector protein SseE; Derived by automated computational analysis using gene prediction method: Protein Homology*	0.669
ssaN	sseD	CY43_07200	CY43_07130	*EscN/YscN/HrcN family type III secretion system ATPase; Derived by automated computational analysis using gene prediction method: Protein Homology*	*SPI-2 type III secretion system translocon protein SseD; May be involved in the translocation of effector proteins into the host cell; Derived by automated computational analysis using gene prediction method: Protein Homology*	0.726
ssaN	sseB	CY43_07200	CY43_07115	*EscN/YscN/HrcN family type III secretion system ATPase; Derived by automated computational analysis using gene prediction method: Protein Homology*	*SPI-2 type III secretion system translocon protein SseB; Necessary for the correct localization of SseC and SseD on the bacterial cell surface; Derived by automated computational analysis using gene prediction method: Protein Homology*	0.880
ssaN	sscB	CY43_07200	CY43_07140	*EscN/YscN/HrcN family type III secretion system ATPase; Derived by automated computational analysis using gene prediction method: Protein Homology*	*Salmonella pathogenicity island 2 protein; member of a type III secretion system involved in the survival and replication of Salmonella in a host cell; chaperone for SseF; required for the efficient replication of Salmonella in host macrophages; Derived by automated computational analysis using gene prediction method: Protein Homology*	0.884
ssaN	sscA	CY43_07200	CY43_07120	*EscN/YscN/HrcN family type III secretion system ATPase; Derived by automated computational analysis using gene prediction method: Protein Homology*	*CesD/SycD/LcrH family type III secretion system chaperone; May be involved in the translocation of effector proteins into the host cell; Derived by automated computational analysis using gene prediction method: Protein Homology*	0.926
ssaN	ssaV	CY43_07200	CY43_07195	*EscN/YscN/HrcN family type III secretion system ATPase; Derived by automated*	*SPI-2 type III secretion system apparatus protein SsaV; With SsaC forms part of a protein export system across the inner and outer cell*	0.980

				computational analysis using gene prediction method: Protein Homology	*membranes; part of the Salmonella pathogenicity island 2; part of the type III secretion system; Derived by automated computational analysis using gene prediction method: Protein Homology*	
ssaN	ssaU	CY43_07200	CY43_07235	*EscN/YscN/HrcN family type III secretion system ATPase; Derived by automated computational analysis using gene prediction method: Protein Homology*	*Member of a type III secretion system which is part of a pathogenicity island in Salmonella, Yersinia and pathogenic Escherichia coli; Derived by automated computational analysis using gene prediction method: Protein Homology*	0.989
ssaN	ssaS	CY43_07200	CY43_07225	*EscN/YscN/HrcN family type III secretion system ATPase; Derived by automated computational analysis using gene prediction method: Protein Homology*	*SPI-2 type III secretion system apparatus protein SsaS; Derived by automated computational analysis using gene prediction method: Protein Homology*	0.986
ssaN	ssaR	CY43_07200	CY43_07220	*EscN/YscN/HrcN family type III secretion system ATPase; Derived by automated computational analysis using gene prediction method: Protein Homology*	*SPI-2 type III secretion system export apparatus protein SsaR; Part of a set of proteins involved in the infection of eukaryotic cells; in plant pathogens involved in the hypersensitivity response; Derived by automated computational analysis using gene prediction method: Protein Homology*	0.986
ssaN	ssaQ	CY43_07200	CY43_07215	*EscN/YscN/HrcN family type III secretion system ATPase; Derived by automated computational analysis using gene prediction method: Protein Homology*	*SPI-2 type III secretion system apparatus protein SsaQ; Derived by automated computational analysis using gene prediction method: Protein Homology*	0.979
ssaN	ssaP	CY43_07200	CY43_07210	*EscN/YscN/HrcN family type III secretion system ATPase; Derived by automated computational analysis using gene prediction method: Protein Homology*	*Salmonella pathogenicity island 2 protein; member of a type III secretion system involved in the survival and replication of Salmonella in a host cell; Derived by automated computational analysis using gene prediction method: Protein Homology*	0.969
ssaN	ssaO	CY43_07200	CY43_07205	*EscN/YscN/HrcN family type III secretion system ATPase; Derived by automated computational analysis using gene prediction method: Protein Homology*	*Salmonella pathogenicity island 2 protein; member of a type III secretion system involved in the survival and replication of Salmonella in a host cell; Derived by automated computational analysis using gene prediction method: Protein Homology*	0.969

ssaN	ssaI	CY43_07200	CY43_07165	*EscN/YscN/HrcN family type III secretion system ATPase; Derived by automated computational analysis using gene prediction method: Protein Homology*	*Salmonella pathogenicity island 2 protein; member of a type III secretion system involved in the survival and replication of Salmonella in a host cell; Derived by automated computational analysis using gene prediction method: Protein Homology*	0.817
ssaN	ssaG	CY43_07200	CY43_07155	*EscN/YscN/HrcN family type III secretion system ATPase; Derived by automated computational analysis using gene prediction method: Protein Homology*	*EscF/YscF/HrpA family type III secretion system needle major subunit; Salmonella pathogenicity island 2 protein; member of a type III secretion system involved in the survival and replication of Salmonella in a host cell; Derived by automated computational analysis using gene prediction method: Protein Homology*	0.764
ssaN	ssaE	CY43_07200	CY43_07105	*EscN/YscN/HrcN family type III secretion system ATPase; Derived by automated computational analysis using gene prediction method: Protein Homology*	*Salmonella pathogenicity island 2 protein; member of a type III secretion system involved in the survival and replication of Salmonella in a host cell; involved in the secretion of SseB; Derived by automated computational analysis using gene prediction method: Protein Homology*	0.687
ssaN	ssaD	CY43_07200	CY43_07100	*EscN/YscN/HrcN family type III secretion system ATPase; Derived by automated computational analysis using gene prediction method: Protein Homology*	*Salmonella pathogenicity island 2 protein; member of a type III secretion system involved in the survival and replication of Salmonella in a host cell; Derived by automated computational analysis using gene prediction method: Protein Homology*	0.830
ssaN	spiC	CY43_07200	CY43_07090	*EscN/YscN/HrcN family type III secretion system ATPase; Derived by automated computational analysis using gene prediction method: Protein Homology*	*SPI-2 type III secretion system protein SpiC; Involved in macrophage infection; inhibits phagosome-lysosome fusion and cellular trafficking; Derived by automated computational analysis using gene prediction method: Protein Homology*	0.607
ssaN	spiA	CY43_07200	CY43_07095	*EscN/YscN/HrcN family type III secretion system ATPase; Derived by automated computational analysis using gene prediction method: Protein Homology*	*SPI-2 type III secretion system protein SpiA; Derived by automated computational analysis using gene prediction method: Protein Homology*	0.789

ssaN	DD95_20700	CY43_07200	CY43_07230	*EscN/YscN/HrcN family type III secretion system ATPase; Derived by automated computational analysis using gene prediction method: Protein Homology*	*Salmonella pathogenicity island 2 protein; member of a type III secretion system involved in the survival and replication of Salmonella in a host cell; Derived by automated computational analysis using gene prediction method: Protein Homology*	0.945
ssaI	ssrA	CY43_07165	CY43_07085	*Salmonella pathogenicity island 2 protein; member of a type III secretion system involved in the survival and replication of Salmonella in a host cell; Derived by automated computational analysis using gene prediction method: Protein Homology*	*Histidine kinase; Phosphorylates the response regulator SsrB; is involved in the expression of the virulence genes of Salmonella pathogenicity island-2; Derived by automated computational analysis using gene prediction method: Protein Homology*	0.556
ssaI	sseF	CY43_07165	CY43_07145	*Salmonella pathogenicity island 2 protein; member of a type III secretion system involved in the survival and replication of Salmonella in a host cell; Derived by automated computational analysis using gene prediction method: Protein Homology*	*Pathogenicity island 2 effector protein SseF; With SseG is involved in the aggregation of the host endosomes; Derived by automated computational analysis using gene prediction method: Protein Homology*	0.894
ssaI	sseE	CY43_07165	CY43_07135	*Salmonella pathogenicity island 2 protein; member of a type III secretion system involved in the survival and replication of Salmonella in a host cell; Derived by automated computational analysis using gene prediction method: Protein Homology*	*Pathogenicity island 2 effector protein SseE; Derived by automated computational analysis using gene prediction method: Protein Homology*	0.716
ssaI	sseD	CY43_07165	CY43_07130	*Salmonella pathogenicity island 2 protein; member of a type III secretion system involved in the survival and replication of Salmonella in a host cell; Derived by automated computational analysis using gene prediction method: Protein Homology*	*SPI-2 type III secretion system translocon protein SseD; May be involved in the translocation of effector proteins into the host cell; Derived by automated computational analysis using gene prediction method: Protein Homology*	0.878
ssaI	sseB	CY43_07165	CY43_07115	*Salmonella pathogenicity island 2 protein; member of a type III secretion system involved in the survival and replication of Salmonella in a host cell; Derived by automated computational analysis using gene prediction method: Protein Homology*	*SPI-2 type III secretion system translocon protein SseB; Necessary for the correct localization of SseC and SseD on the bacterial cell surface; Derived by automated computational analysis using gene prediction method: Protein Homology*	0.764
ssaI	sscB	CY43_07165	CY43_07140	*Salmonella pathogenicity island 2 protein; member of a type III secretion system involved*	*Salmonella pathogenicity island 2 protein; member of a type III secretion system involved*	0.720

				in the survival and replication of Salmonella in a host cell; Derived by automated computational analysis using gene prediction method: Protein Homology	*in the survival and replication of Salmonella in a host cell; chaperone for SseF; required for the efficient replication of Salmonella in host macrophages; Derived by automated computational analysis using gene prediction method: Protein Homology*	
ssaI	sscA	CY43_07165	CY43_07120	*Salmonella pathogenicity island 2 protein; member of a type III secretion system involved in the survival and replication of Salmonella in a host cell; Derived by automated computational analysis using gene prediction method: Protein Homology*	*CesD/SycD/LcrH family type III secretion system chaperone; May be involved in the translocation of effector proteins into the host cell; Derived by automated computational analysis using gene prediction method: Protein Homology*	0.728
ssaI	ssaV	CY43_07165	CY43_07195	*Salmonella pathogenicity island 2 protein; member of a type III secretion system involved in the survival and replication of Salmonella in a host cell; Derived by automated computational analysis using gene prediction method: Protein Homology*	*SPI-2 type III secretion system apparatus protein SsaV; With SsaC forms part of a protein export system across the inner and outer cell membranes; part of the Salmonella pathogenicity island 2; part of the type III secretion system; Derived by automated computational analysis using gene prediction method: Protein Homology*	0.763
ssaI	ssaU	CY43_07165	CY43_07235	*Salmonella pathogenicity island 2 protein; member of a type III secretion system involved in the survival and replication of Salmonella in a host cell; Derived by automated computational analysis using gene prediction method: Protein Homology*	*Member of a type III secretion system which is part of a pathogenicity island in Salmonella, Yersinia and pathogenic Escherichia coli; Derived by automated computational analysis using gene prediction method: Protein Homology*	0.702
ssaI	ssaS	CY43_07165	CY43_07225	*Salmonella pathogenicity island 2 protein; member of a type III secretion system involved in the survival and replication of Salmonella in a host cell; Derived by automated computational analysis using gene prediction method: Protein Homology*	*SPI-2 type III secretion system apparatus protein SsaS; Derived by automated computational analysis using gene prediction method: Protein Homology*	0.841
ssaI	ssaR	CY43_07165	CY43_07220	*Salmonella pathogenicity island 2 protein; member of a type III secretion system involved in the survival and replication of Salmonella in a host cell; Derived by automated computational analysis using gene prediction method: Protein Homology*	*SPI-2 type III secretion system export apparatus protein SsaR; Part of a set of proteins involved in the infection of eukaryotic cells; in plant pathogens involved in the hypersensitivity response; Derived by automated computational*	0.751

					analysis using gene prediction method: Protein Homology	
ssaI	ssaQ	CY43_07165	CY43_07215	*Salmonella pathogenicity island 2 protein; member of a type III secretion system involved in the survival and replication of Salmonella in a host cell; Derived by automated computational analysis using gene prediction method: Protein Homology*	*SPI-2 type III secretion system apparatus protein SsaQ; Derived by automated computational analysis using gene prediction method: Protein Homology*	0.821
ssaI	ssaP	CY43_07165	CY43_07210	*Salmonella pathogenicity island 2 protein; member of a type III secretion system involved in the survival and replication of Salmonella in a host cell; Derived by automated computational analysis using gene prediction method: Protein Homology*	*Salmonella pathogenicity island 2 protein; member of a type III secretion system involved in the survival and replication of Salmonella in a host cell; Derived by automated computational analysis using gene prediction method: Protein Homology*	0.757
ssaI	ssaO	CY43_07165	CY43_07205	*Salmonella pathogenicity island 2 protein; member of a type III secretion system involved in the survival and replication of Salmonella in a host cell; Derived by automated computational analysis using gene prediction method: Protein Homology*	*Salmonella pathogenicity island 2 protein; member of a type III secretion system involved in the survival and replication of Salmonella in a host cell; Derived by automated computational analysis using gene prediction method: Protein Homology*	0.759
ssaI	ssaN	CY43_07165	CY43_07200	*Salmonella pathogenicity island 2 protein; member of a type III secretion system involved in the survival and replication of Salmonella in a host cell; Derived by automated computational analysis using gene prediction method: Protein Homology*	*EscN/YscN/HrcN family type III secretion system ATPase; Derived by automated computational analysis using gene prediction method: Protein Homology*	0.817
ssaI	ssaG	CY43_07165	CY43_07155	*Salmonella pathogenicity island 2 protein; member of a type III secretion system involved in the survival and replication of Salmonella in a host cell; Derived by automated computational analysis using gene prediction method: Protein Homology*	*EscF/YscF/HrpA family type III secretion system needle major subunit; Salmonella pathogenicity island 2 protein; member of a type III secretion system involved in the survival and replication of Salmonella in a host cell; Derived by automated computational analysis using gene prediction method: Protein Homology*	0.939
ssaI	ssaE	CY43_07165	CY43_07105	*Salmonella pathogenicity island 2 protein; member of a type III secretion system involved in the survival and replication of Salmonella in a host cell; Derived by automated*	*Salmonella pathogenicity island 2 protein; member of a type III secretion system involved in the survival and replication of Salmonella in a host cell; involved in the secretion of SseB;*	0.537

				computational analysis using gene prediction method: Protein Homology	*Derived by automated computational analysis using gene prediction method: Protein Homology*	
ssaI	ssaD	CY43_07165	CY43_07100	*Salmonella pathogenicity island 2 protein; member of a type III secretion system involved in the survival and replication of Salmonella in a host cell; Derived by automated computational analysis using gene prediction method: Protein Homology*	*Salmonella pathogenicity island 2 protein; member of a type III secretion system involved in the survival and replication of Salmonella in a host cell; Derived by automated computational analysis using gene prediction method: Protein Homology*	0.535
ssaI	spiC	CY43_07165	CY43_07090	*Salmonella pathogenicity island 2 protein; member of a type III secretion system involved in the survival and replication of Salmonella in a host cell; Derived by automated computational analysis using gene prediction method: Protein Homology*	*SPI-2 type III secretion system protein SpiC; Involved in macrophage infection; inhibits phagosome-lysosome fusion and cellular trafficking; Derived by automated computational analysis using gene prediction method: Protein Homology*	0.538
ssaI	spiA	CY43_07165	CY43_07095	*Salmonella pathogenicity island 2 protein; member of a type III secretion system involved in the survival and replication of Salmonella in a host cell; Derived by automated computational analysis using gene prediction method: Protein Homology*	*SPI-2 type III secretion system protein SpiA; Derived by automated computational analysis using gene prediction method: Protein Homology*	0.537
ssaI	DD95_20700	CY43_07165	CY43_07230	*Salmonella pathogenicity island 2 protein; member of a type III secretion system involved in the survival and replication of Salmonella in a host cell; Derived by automated computational analysis using gene prediction method: Protein Homology*	*Salmonella pathogenicity island 2 protein; member of a type III secretion system involved in the survival and replication of Salmonella in a host cell; Derived by automated computational analysis using gene prediction method: Protein Homology*	0.746
ssaG	ssrB	CY43_07155	CY43_07080	*EscF/YscF/HrpA family type III secretion system needle major subunit; Salmonella pathogenicity island 2 protein; member of a type III secretion system involved in the survival and replication of Salmonella in a host cell; Derived by automated computational analysis using gene prediction method: Protein Homology*	*Type III secretion system regulator; Is phosphorylated by SsrA; is involved in the expression of the virulence genes of Salmonella pathogenicity island-2; Derived by automated computational analysis using gene prediction method: Protein Homology*	0.919
ssaG	ssrA	CY43_07155	CY43_07085	*EscF/YscF/HrpA family type III secretion system needle major subunit; Salmonella pathogenicity island 2 protein; member of a*	*Histidine kinase; Phosphorylates the response regulator SsrB; is involved in the expression of the virulence genes of Salmonella pathogenicity*	0.903

				type III secretion system involved in the survival and replication of Salmonella in a host cell; Derived by automated computational analysis using gene prediction method: Protein Homology	*island-2; Derived by automated computational analysis using gene prediction method: Protein Homology*	
ssaG	sseF	CY43_07155	CY43_07145	*EscF/YscF/HrpA family type III secretion system needle major subunit; Salmonella pathogenicity island 2 protein; member of a type III secretion system involved in the survival and replication of Salmonella in a host cell; Derived by automated computational analysis using gene prediction method: Protein Homology*	*Pathogenicity island 2 effector protein SseF; With SseG is involved in the aggregation of the host endosomes; Derived by automated computational analysis using gene prediction method: Protein Homology*	0.715
ssaG	sseE	CY43_07155	CY43_07135	*EscF/YscF/HrpA family type III secretion system needle major subunit; Salmonella pathogenicity island 2 protein; member of a type III secretion system involved in the survival and replication of Salmonella in a host cell; Derived by automated computational analysis using gene prediction method: Protein Homology*	*Pathogenicity island 2 effector protein SseE; Derived by automated computational analysis using gene prediction method: Protein Homology*	0.656
ssaG	sseD	CY43_07155	CY43_07130	*EscF/YscF/HrpA family type III secretion system needle major subunit; Salmonella pathogenicity island 2 protein; member of a type III secretion system involved in the survival and replication of Salmonella in a host cell; Derived by automated computational analysis using gene prediction method: Protein Homology*	*SPI-2 type III secretion system translocon protein SseD; May be involved in the translocation of effector proteins into the host cell; Derived by automated computational analysis using gene prediction method: Protein Homology*	0.888
ssaG	sseB	CY43_07155	CY43_07115	*EscF/YscF/HrpA family type III secretion system needle major subunit; Salmonella pathogenicity island 2 protein; member of a type III secretion system involved in the survival and replication of Salmonella in a host cell; Derived by automated computational analysis using gene prediction method: Protein Homology*	*SPI-2 type III secretion system translocon protein SseB; Necessary for the correct localization of SseC and SseD on the bacterial cell surface; Derived by automated computational analysis using gene prediction method: Protein Homology*	0.878

ssaG	sscB	CY43_07155	CY43_07140	*EscF/YscF/HrpA family type III secretion system needle major subunit; Salmonella pathogenicity island 2 protein; member of a type III secretion system involved in the survival and replication of Salmonella in a host cell; Derived by automated computational analysis using gene prediction method: Protein Homology*	*Salmonella pathogenicity island 2 protein; member of a type III secretion system involved in the survival and replication of Salmonella in a host cell; chaperone for SseF; required for the efficient replication of Salmonella in host macrophages; Derived by automated computational analysis using gene prediction method: Protein Homology*	0.717
ssaG	sscA	CY43_07155	CY43_07120	*EscF/YscF/HrpA family type III secretion system needle major subunit; Salmonella pathogenicity island 2 protein; member of a type III secretion system involved in the survival and replication of Salmonella in a host cell; Derived by automated computational analysis using gene prediction method: Protein Homology*	*CesD/SycD/LcrH family type III secretion system chaperone; May be involved in the translocation of effector proteins into the host cell; Derived by automated computational analysis using gene prediction method: Protein Homology*	0.862
ssaG	ssaV	CY43_07155	CY43_07195	*EscF/YscF/HrpA family type III secretion system needle major subunit; Salmonella pathogenicity island 2 protein; member of a type III secretion system involved in the survival and replication of Salmonella in a host cell; Derived by automated computational analysis using gene prediction method: Protein Homology*	*SPI-2 type III secretion system apparatus protein SsaV; With SsaC forms part of a protein export system across the inner and outer cell membranes; part of the Salmonella pathogenicity island 2; part of the type III secretion system; Derived by automated computational analysis using gene prediction method: Protein Homology*	0.813
ssaG	ssaU	CY43_07155	CY43_07235	*EscF/YscF/HrpA family type III secretion system needle major subunit; Salmonella pathogenicity island 2 protein; member of a type III secretion system involved in the survival and replication of Salmonella in a host cell; Derived by automated computational analysis using gene prediction method: Protein Homology*	*Member of a type III secretion system which is part of a pathogenicity island in Salmonella, Yersinia and pathogenic Escherichia coli; Derived by automated computational analysis using gene prediction method: Protein Homology*	0.790
ssaG	ssaS	CY43_07155	CY43_07225	*EscF/YscF/HrpA family type III secretion system needle major subunit; Salmonella pathogenicity island 2 protein; member of a type III secretion system involved in the survival and replication of Salmonella in a host cell; Derived by automated computational analysis*	*SPI-2 type III secretion system apparatus protein SsaS; Derived by automated computational analysis using gene prediction method: Protein Homology*	0.735

				using gene prediction method: Protein Homology		
ssaG	ssaR	CY43_07155	CY43_07220	*EscF/YscF/HrpA family type III secretion system needle major subunit; Salmonella pathogenicity island 2 protein; member of a type III secretion system involved in the survival and replication of Salmonella in a host cell; Derived by automated computational analysis using gene prediction method: Protein Homology*	*SPI-2 type III secretion system export apparatus protein SsaR; Part of a set of proteins involved in the infection of eukaryotic cells; in plant pathogens involved in the hypersensitivity response; Derived by automated computational analysis using gene prediction method: Protein Homology*	0.893
ssaG	ssaQ	CY43_07155	CY43_07215	*EscF/YscF/HrpA family type III secretion system needle major subunit; Salmonella pathogenicity island 2 protein; member of a type III secretion system involved in the survival and replication of Salmonella in a host cell; Derived by automated computational analysis using gene prediction method: Protein Homology*	*SPI-2 type III secretion system apparatus protein SsaQ; Derived by automated computational analysis using gene prediction method: Protein Homology*	0.754
ssaG	ssaP	CY43_07155	CY43_07210	*EscF/YscF/HrpA family type III secretion system needle major subunit; Salmonella pathogenicity island 2 protein; member of a type III secretion system involved in the survival and replication of Salmonella in a host cell; Derived by automated computational analysis using gene prediction method: Protein Homology*	*Salmonella pathogenicity island 2 protein; member of a type III secretion system involved in the survival and replication of Salmonella in a host cell; Derived by automated computational analysis using gene prediction method: Protein Homology*	0.747
ssaG	ssaO	CY43_07155	CY43_07205	*EscF/YscF/HrpA family type III secretion system needle major subunit; Salmonella pathogenicity island 2 protein; member of a type III secretion system involved in the survival and replication of Salmonella in a host cell; Derived by automated computational analysis using gene prediction method: Protein Homology*	*Salmonella pathogenicity island 2 protein; member of a type III secretion system involved in the survival and replication of Salmonella in a host cell; Derived by automated computational analysis using gene prediction method: Protein Homology*	0.750
ssaG	ssaN	CY43_07155	CY43_07200	*EscF/YscF/HrpA family type III secretion system needle major subunit; Salmonella pathogenicity island 2 protein; member of a type III secretion system involved in the survival*	*EscN/YscN/HrcN family type III secretion system ATPase; Derived by automated computational analysis using gene prediction method: Protein Homology*	0.764

				and replication of Salmonella in a host cell; Derived by automated computational analysis using gene prediction method: Protein Homology		
ssaG	ssaI	CY43_07155	CY43_07165	*EscF/YscF/HrpA family type III secretion system needle major subunit; Salmonella pathogenicity island 2 protein; member of a type III secretion system involved in the survival and replication of Salmonella in a host cell; Derived by automated computational analysis using gene prediction method: Protein Homology*	*Salmonella pathogenicity island 2 protein; member of a type III secretion system involved in the survival and replication of Salmonella in a host cell; Derived by automated computational analysis using gene prediction method: Protein Homology*	0.939
ssaG	ssaE	CY43_07155	CY43_07105	*EscF/YscF/HrpA family type III secretion system needle major subunit; Salmonella pathogenicity island 2 protein; member of a type III secretion system involved in the survival and replication of Salmonella in a host cell; Derived by automated computational analysis using gene prediction method: Protein Homology*	*Salmonella pathogenicity island 2 protein; member of a type III secretion system involved in the survival and replication of Salmonella in a host cell; involved in the secretion of SseB; Derived by automated computational analysis using gene prediction method: Protein Homology*	0.716
ssaG	ssaD	CY43_07155	CY43_07100	*EscF/YscF/HrpA family type III secretion system needle major subunit; Salmonella pathogenicity island 2 protein; member of a type III secretion system involved in the survival and replication of Salmonella in a host cell; Derived by automated computational analysis using gene prediction method: Protein Homology*	*Salmonella pathogenicity island 2 protein; member of a type III secretion system involved in the survival and replication of Salmonella in a host cell; Derived by automated computational analysis using gene prediction method: Protein Homology*	0.642
ssaG	spiC	CY43_07155	CY43_07090	*EscF/YscF/HrpA family type III secretion system needle major subunit; Salmonella pathogenicity island 2 protein; member of a type III secretion system involved in the survival and replication of Salmonella in a host cell; Derived by automated computational analysis using gene prediction method: Protein Homology*	*SPI-2 type III secretion system protein SpiC; Involved in macrophage infection; inhibits phagosome-lysosome fusion and cellular trafficking; Derived by automated computational analysis using gene prediction method: Protein Homology*	0.937
ssaG	spiA	CY43_07155	CY43_07095	*EscF/YscF/HrpA family type III secretion system needle major subunit; Salmonella*	*SPI-2 type III secretion system protein SpiA; Derived by automated computational analysis*	0.775

				pathogenicity island 2 protein; member of a type III secretion system involved in the survival and replication of Salmonella in a host cell; Derived by automated computational analysis using gene prediction method: Protein Homology	*using gene prediction method: Protein Homology*	
ssaG	DD95_20700	CY43_07155	CY43_07230	*EscF/YscF/HrpA family type III secretion system needle major subunit; Salmonella pathogenicity island 2 protein; member of a type III secretion system involved in the survival and replication of Salmonella in a host cell; Derived by automated computational analysis using gene prediction method: Protein Homology*	*Salmonella pathogenicity island 2 protein; member of a type III secretion system involved in the survival and replication of Salmonella in a host cell; Derived by automated computational analysis using gene prediction method: Protein Homology*	0.746
ssaE	ssrB	CY43_07105	CY43_07080	*Salmonella pathogenicity island 2 protein; member of a type III secretion system involved in the survival and replication of Salmonella in a host cell; involved in the secretion of SseB; Derived by automated computational analysis using gene prediction method: Protein Homology*	*Type III secretion system regulator; Is phosphorylated by SsrA; is involved in the expression of the virulence genes of Salmonella pathogenicity island-2; Derived by automated computational analysis using gene prediction method: Protein Homology*	0.674
ssaE	ssrA	CY43_07105	CY43_07085	*Salmonella pathogenicity island 2 protein; member of a type III secretion system involved in the survival and replication of Salmonella in a host cell; involved in the secretion of SseB; Derived by automated computational analysis using gene prediction method: Protein Homology*	*Histidine kinase; Phosphorylates the response regulator SsrB; is involved in the expression of the virulence genes of Salmonella pathogenicity island-2; Derived by automated computational analysis using gene prediction method: Protein Homology*	0.690
ssaE	sseF	CY43_07105	CY43_07145	*Salmonella pathogenicity island 2 protein; member of a type III secretion system involved in the survival and replication of Salmonella in a host cell; involved in the secretion of SseB; Derived by automated computational analysis using gene prediction method: Protein Homology*	*Pathogenicity island 2 effector protein SseF; With SseG is involved in the aggregation of the host endosomes; Derived by automated computational analysis using gene prediction method: Protein Homology*	0.576
ssaE	sseE	CY43_07105	CY43_07135	*Salmonella pathogenicity island 2 protein; member of a type III secretion system involved in the survival and replication of Salmonella in*	*Pathogenicity island 2 effector protein SseE; Derived by automated computational analysis*	0.843

				a host cell; involved in the secretion of SseB; Derived by automated computational analysis using gene prediction method: Protein Homology	*using gene prediction method: Protein Homology*	
ssaE	sseD	CY43_07105	CY43_07130	*Salmonella pathogenicity island 2 protein; member of a type III secretion system involved in the survival and replication of Salmonella in a host cell; involved in the secretion of SseB; Derived by automated computational analysis using gene prediction method: Protein Homology*	*SPI-2 type III secretion system translocon protein SseD; May be involved in the translocation of effector proteins into the host cell; Derived by automated computational analysis using gene prediction method: Protein Homology*	0.722
ssaE	sseB	CY43_07105	CY43_07115	*Salmonella pathogenicity island 2 protein; member of a type III secretion system involved in the survival and replication of Salmonella in a host cell; involved in the secretion of SseB; Derived by automated computational analysis using gene prediction method: Protein Homology*	*SPI-2 type III secretion system translocon protein SseB; Necessary for the correct localization of SseC and SseD on the bacterial cell surface; Derived by automated computational analysis using gene prediction method: Protein Homology*	0.923
ssaE	sscB	CY43_07105	CY43_07140	*Salmonella pathogenicity island 2 protein; member of a type III secretion system involved in the survival and replication of Salmonella in a host cell; involved in the secretion of SseB; Derived by automated computational analysis using gene prediction method: Protein Homology*	*Salmonella pathogenicity island 2 protein; member of a type III secretion system involved in the survival and replication of Salmonella in a host cell; chaperone for SseF; required for the efficient replication of Salmonella in host macrophages; Derived by automated computational analysis using gene prediction method: Protein Homology*	0.663
ssaE	sscA	CY43_07105	CY43_07120	*Salmonella pathogenicity island 2 protein; member of a type III secretion system involved in the survival and replication of Salmonella in a host cell; involved in the secretion of SseB; Derived by automated computational analysis using gene prediction method: Protein Homology*	*CesD/SycD/LcrH family type III secretion system chaperone; May be involved in the translocation of effector proteins into the host cell; Derived by automated computational analysis using gene prediction method: Protein Homology*	0.851
ssaE	ssaV	CY43_07105	CY43_07195	*Salmonella pathogenicity island 2 protein; member of a type III secretion system involved in the survival and replication of Salmonella in a host cell; involved in the secretion of SseB; Derived by automated computational analysis*	*SPI-2 type III secretion system apparatus protein SsaV; With SsaC forms part of a protein export system across the inner and outer cell membranes; part of the Salmonella pathogenicity island 2; part of the type III*	0.623

				using gene prediction method: Protein Homology	*secretion system; Derived by automated computational analysis using gene prediction method: Protein Homology*	
ssaE	ssaU	CY43_07105	CY43_07235	*Salmonella pathogenicity island 2 protein; member of a type III secretion system involved in the survival and replication of Salmonella in a host cell; involved in the secretion of SseB; Derived by automated computational analysis using gene prediction method: Protein Homology*	*Member of a type III secretion system which is part of a pathogenicity island in Salmonella, Yersinia and pathogenic Escherichia coli; Derived by automated computational analysis using gene prediction method: Protein Homology*	0.492
ssaE	ssaS	CY43_07105	CY43_07225	*Salmonella pathogenicity island 2 protein; member of a type III secretion system involved in the survival and replication of Salmonella in a host cell; involved in the secretion of SseB; Derived by automated computational analysis using gene prediction method: Protein Homology*	*SPI-2 type III secretion system apparatus protein SsaS; Derived by automated computational analysis using gene prediction method: Protein Homology*	0.598
ssaE	ssaR	CY43_07105	CY43_07220	*Salmonella pathogenicity island 2 protein; member of a type III secretion system involved in the survival and replication of Salmonella in a host cell; involved in the secretion of SseB; Derived by automated computational analysis using gene prediction method: Protein Homology*	*SPI-2 type III secretion system export apparatus protein SsaR; Part of a set of proteins involved in the infection of eukaryotic cells; in plant pathogens involved in the hypersensitivity response; Derived by automated computational analysis using gene prediction method: Protein Homology*	0.710
ssaE	ssaQ	CY43_07105	CY43_07215	*Salmonella pathogenicity island 2 protein; member of a type III secretion system involved in the survival and replication of Salmonella in a host cell; involved in the secretion of SseB; Derived by automated computational analysis using gene prediction method: Protein Homology*	*SPI-2 type III secretion system apparatus protein SsaQ; Derived by automated computational analysis using gene prediction method: Protein Homology*	0.767
ssaE	ssaP	CY43_07105	CY43_07210	*Salmonella pathogenicity island 2 protein; member of a type III secretion system involved in the survival and replication of Salmonella in a host cell; involved in the secretion of SseB; Derived by automated computational analysis using gene prediction method: Protein Homology*	*Salmonella pathogenicity island 2 protein; member of a type III secretion system involved in the survival and replication of Salmonella in a host cell; Derived by automated computational analysis using gene prediction method: Protein Homology*	0.478

ssaE	ssaO	CY43_07105	CY43_07205	*Salmonella pathogenicity island 2 protein; member of a type III secretion system involved in the survival and replication of Salmonella in a host cell; involved in the secretion of SseB; Derived by automated computational analysis using gene prediction method: Protein Homology*	*Salmonella pathogenicity island 2 protein; member of a type III secretion system involved in the survival and replication of Salmonella in a host cell; Derived by automated computational analysis using gene prediction method: Protein Homology*	0.494
ssaE	ssaN	CY43_07105	CY43_07200	*Salmonella pathogenicity island 2 protein; member of a type III secretion system involved in the survival and replication of Salmonella in a host cell; involved in the secretion of SseB; Derived by automated computational analysis using gene prediction method: Protein Homology*	*EscN/YscN/HrcN family type III secretion system ATPase; Derived by automated computational analysis using gene prediction method: Protein Homology*	0.687
ssaE	ssaI	CY43_07105	CY43_07165	*Salmonella pathogenicity island 2 protein; member of a type III secretion system involved in the survival and replication of Salmonella in a host cell; involved in the secretion of SseB; Derived by automated computational analysis using gene prediction method: Protein Homology*	*Salmonella pathogenicity island 2 protein; member of a type III secretion system involved in the survival and replication of Salmonella in a host cell; Derived by automated computational analysis using gene prediction method: Protein Homology*	0.537
ssaE	ssaG	CY43_07105	CY43_07155	*Salmonella pathogenicity island 2 protein; member of a type III secretion system involved in the survival and replication of Salmonella in a host cell; involved in the secretion of SseB; Derived by automated computational analysis using gene prediction method: Protein Homology*	*EscF/YscF/HrpA family type III secretion system needle major subunit; Salmonella pathogenicity island 2 protein; member of a type III secretion system involved in the survival and replication of Salmonella in a host cell; Derived by automated computational analysis using gene prediction method: Protein Homology*	0.716
ssaE	ssaD	CY43_07105	CY43_07100	*Salmonella pathogenicity island 2 protein; member of a type III secretion system involved in the survival and replication of Salmonella in a host cell; involved in the secretion of SseB; Derived by automated computational analysis using gene prediction method: Protein Homology*	*Salmonella pathogenicity island 2 protein; member of a type III secretion system involved in the survival and replication of Salmonella in a host cell; Derived by automated computational analysis using gene prediction method: Protein Homology*	0.926
ssaE	spiC	CY43_07105	CY43_07090	*Salmonella pathogenicity island 2 protein; member of a type III secretion system involved*	*SPI-2 type III secretion system protein SpiC; Involved in macrophage infection; inhibits*	0.930

				in the survival and replication of Salmonella in a host cell; involved in the secretion of SseB; Derived by automated computational analysis using gene prediction method: Protein Homology	*phagosome-lysosome fusion and cellular trafficking; Derived by automated computational analysis using gene prediction method: Protein Homology*	
ssaE	spiA	CY43_07105	CY43_07095	*Salmonella pathogenicity island 2 protein; member of a type III secretion system involved in the survival and replication of Salmonella in a host cell; involved in the secretion of SseB; Derived by automated computational analysis using gene prediction method: Protein Homology*	*SPI-2 type III secretion system protein SpiA; Derived by automated computational analysis using gene prediction method: Protein Homology*	0.927
ssaE	DD95_20700	CY43_07105	CY43_07230	*Salmonella pathogenicity island 2 protein; member of a type III secretion system involved in the survival and replication of Salmonella in a host cell; involved in the secretion of SseB; Derived by automated computational analysis using gene prediction method: Protein Homology*	*Salmonella pathogenicity island 2 protein; member of a type III secretion system involved in the survival and replication of Salmonella in a host cell; Derived by automated computational analysis using gene prediction method: Protein Homology*	0.610
ssaD	ssrB	CY43_07100	CY43_07080	*Salmonella pathogenicity island 2 protein; member of a type III secretion system involved in the survival and replication of Salmonella in a host cell; Derived by automated computational analysis using gene prediction method: Protein Homology*	*Type III secretion system regulator; Is phosphorylated by SsrA; is involved in the expression of the virulence genes of Salmonella pathogenicity island-2; Derived by automated computational analysis using gene prediction method: Protein Homology*	0.717
ssaD	ssrA	CY43_07100	CY43_07085	*Salmonella pathogenicity island 2 protein; member of a type III secretion system involved in the survival and replication of Salmonella in a host cell; Derived by automated computational analysis using gene prediction method: Protein Homology*	*Histidine kinase; Phosphorylates the response regulator SsrB; is involved in the expression of the virulence genes of Salmonella pathogenicity island-2; Derived by automated computational analysis using gene prediction method: Protein Homology*	0.923
ssaD	sseF	CY43_07100	CY43_07145	*Salmonella pathogenicity island 2 protein; member of a type III secretion system involved in the survival and replication of Salmonella in a host cell; Derived by automated computational analysis using gene prediction method: Protein Homology*	*Pathogenicity island 2 effector protein SseF; With SseG is involved in the aggregation of the host endosomes; Derived by automated computational analysis using gene prediction method: Protein Homology*	0.628

ssaD	sseE	CY43_07100	CY43_07135	*Salmonella pathogenicity island 2 protein; member of a type III secretion system involved in the survival and replication of Salmonella in a host cell; Derived by automated computational analysis using gene prediction method: Protein Homology*	*Pathogenicity island 2 effector protein SseE; Derived by automated computational analysis using gene prediction method: Protein Homology*	0.884
ssaD	sseD	CY43_07100	CY43_07130	*Salmonella pathogenicity island 2 protein; member of a type III secretion system involved in the survival and replication of Salmonella in a host cell; Derived by automated computational analysis using gene prediction method: Protein Homology*	*SPI-2 type III secretion system translocon protein SseD; May be involved in the translocation of effector proteins into the host cell; Derived by automated computational analysis using gene prediction method: Protein Homology*	0.792
ssaD	sseB	CY43_07100	CY43_07115	*Salmonella pathogenicity island 2 protein; member of a type III secretion system involved in the survival and replication of Salmonella in a host cell; Derived by automated computational analysis using gene prediction method: Protein Homology*	*SPI-2 type III secretion system translocon protein SseB; Necessary for the correct localization of SseC and SseD on the bacterial cell surface; Derived by automated computational analysis using gene prediction method: Protein Homology*	0.851
ssaD	sscB	CY43_07100	CY43_07140	*Salmonella pathogenicity island 2 protein; member of a type III secretion system involved in the survival and replication of Salmonella in a host cell; Derived by automated computational analysis using gene prediction method: Protein Homology*	*Salmonella pathogenicity island 2 protein; member of a type III secretion system involved in the survival and replication of Salmonella in a host cell; chaperone for SseF; required for the efficient replication of Salmonella in host macrophages; Derived by automated computational analysis using gene prediction method: Protein Homology*	0.955
ssaD	sscA	CY43_07100	CY43_07120	*Salmonella pathogenicity island 2 protein; member of a type III secretion system involved in the survival and replication of Salmonella in a host cell; Derived by automated computational analysis using gene prediction method: Protein Homology*	*CesD/SycD/LcrH family type III secretion system chaperone; May be involved in the translocation of effector proteins into the host cell; Derived by automated computational analysis using gene prediction method: Protein Homology*	0.975
ssaD	ssaV	CY43_07100	CY43_07195	*Salmonella pathogenicity island 2 protein; member of a type III secretion system involved in the survival and replication of Salmonella in a host cell; Derived by automated computational analysis using gene prediction method: Protein Homology*	*SPI-2 type III secretion system apparatus protein SsaV; With SsaC forms part of a protein export system across the inner and outer cell membranes; part of the Salmonella pathogenicity island 2; part of the type III secretion system; Derived by automated*	0.731

					computational analysis using gene prediction method: Protein Homology	
ssaD	ssaU	CY43_07100	CY43_07235	*Salmonella pathogenicity island 2 protein; member of a type III secretion system involved in the survival and replication of Salmonella in a host cell; Derived by automated computational analysis using gene prediction method: Protein Homology*	*Member of a type III secretion system which is part of a pathogenicity island in Salmonella, Yersinia and pathogenic Escherichia coli; Derived by automated computational analysis using gene prediction method: Protein Homology*	0.902
ssaD	ssaS	CY43_07100	CY43_07225	*Salmonella pathogenicity island 2 protein; member of a type III secretion system involved in the survival and replication of Salmonella in a host cell; Derived by automated computational analysis using gene prediction method: Protein Homology*	*SPI-2 type III secretion system apparatus protein SsaS; Derived by automated computational analysis using gene prediction method: Protein Homology*	0.860
ssaD	ssaR	CY43_07100	CY43_07220	*Salmonella pathogenicity island 2 protein; member of a type III secretion system involved in the survival and replication of Salmonella in a host cell; Derived by automated computational analysis using gene prediction method: Protein Homology*	*SPI-2 type III secretion system export apparatus protein SsaR; Part of a set of proteins involved in the infection of eukaryotic cells; in plant pathogens involved in the hypersensitivity response; Derived by automated computational analysis using gene prediction method: Protein Homology*	0.888
ssaD	ssaQ	CY43_07100	CY43_07215	*Salmonella pathogenicity island 2 protein; member of a type III secretion system involved in the survival and replication of Salmonella in a host cell; Derived by automated computational analysis using gene prediction method: Protein Homology*	*SPI-2 type III secretion system apparatus protein SsaQ; Derived by automated computational analysis using gene prediction method: Protein Homology*	0.964
ssaD	ssaP	CY43_07100	CY43_07210	*Salmonella pathogenicity island 2 protein; member of a type III secretion system involved in the survival and replication of Salmonella in a host cell; Derived by automated computational analysis using gene prediction method: Protein Homology*	*Salmonella pathogenicity island 2 protein; member of a type III secretion system involved in the survival and replication of Salmonella in a host cell; Derived by automated computational analysis using gene prediction method: Protein Homology*	0.840
ssaD	ssaO	CY43_07100	CY43_07205	*Salmonella pathogenicity island 2 protein; member of a type III secretion system involved in the survival and replication of Salmonella in a host cell; Derived by automated*	*Salmonella pathogenicity island 2 protein; member of a type III secretion system involved in the survival and replication of Salmonella in a host cell; Derived by automated*	0.839

				computational analysis using gene prediction method: Protein Homology	*computational analysis using gene prediction method: Protein Homology*	
ssaD	ssaN	CY43_07100	CY43_07200	*Salmonella pathogenicity island 2 protein; member of a type III secretion system involved in the survival and replication of Salmonella in a host cell; Derived by automated computational analysis using gene prediction method: Protein Homology*	*EscN/YscN/HrcN family type III secretion system ATPase; Derived by automated computational analysis using gene prediction method: Protein Homology*	0.830
ssaD	ssaI	CY43_07100	CY43_07165	*Salmonella pathogenicity island 2 protein; member of a type III secretion system involved in the survival and replication of Salmonella in a host cell; Derived by automated computational analysis using gene prediction method: Protein Homology*	*Salmonella pathogenicity island 2 protein; member of a type III secretion system involved in the survival and replication of Salmonella in a host cell; Derived by automated computational analysis using gene prediction method: Protein Homology*	0.535
ssaD	ssaG	CY43_07100	CY43_07155	*Salmonella pathogenicity island 2 protein; member of a type III secretion system involved in the survival and replication of Salmonella in a host cell; Derived by automated computational analysis using gene prediction method: Protein Homology*	*EscF/YscF/HrpA family type III secretion system needle major subunit; Salmonella pathogenicity island 2 protein; member of a type III secretion system involved in the survival and replication of Salmonella in a host cell; Derived by automated computational analysis using gene prediction method: Protein Homology*	0.642
ssaD	ssaE	CY43_07100	CY43_07105	*Salmonella pathogenicity island 2 protein; member of a type III secretion system involved in the survival and replication of Salmonella in a host cell; Derived by automated computational analysis using gene prediction method: Protein Homology*	*Salmonella pathogenicity island 2 protein; member of a type III secretion system involved in the survival and replication of Salmonella in a host cell; involved in the secretion of SseB; Derived by automated computational analysis using gene prediction method: Protein Homology*	0.926
ssaD	spiC	CY43_07100	CY43_07090	*Salmonella pathogenicity island 2 protein; member of a type III secretion system involved in the survival and replication of Salmonella in a host cell; Derived by automated computational analysis using gene prediction method: Protein Homology*	*SPI-2 type III secretion system protein SpiC; Involved in macrophage infection; inhibits phagosome-lysosome fusion and cellular trafficking; Derived by automated computational analysis using gene prediction method: Protein Homology*	0.955
ssaD	spiA	CY43_07100	CY43_07095	*Salmonella pathogenicity island 2 protein; member of a type III secretion system involved in the survival and replication of Salmonella in*	*SPI-2 type III secretion system protein SpiA; Derived by automated computational analysis*	0.988

				a host cell; Derived by automated computational analysis using gene prediction method: Protein Homology	*using gene prediction method: Protein Homology*	
ssaD	DD95_20700	CY43_07100	CY43_07230	*Salmonella pathogenicity island 2 protein; member of a type III secretion system involved in the survival and replication of Salmonella in a host cell; Derived by automated computational analysis using gene prediction method: Protein Homology*	*Salmonella pathogenicity island 2 protein; member of a type III secretion system involved in the survival and replication of Salmonella in a host cell; Derived by automated computational analysis using gene prediction method: Protein Homology*	0.892
spiC	ssrB	CY43_07090	CY43_07080	*SPI-2 type III secretion system protein SpiC; Involved in macrophage infection; inhibits phagosome-lysosome fusion and cellular trafficking; Derived by automated computational analysis using gene prediction method: Protein Homology*	*Type III secretion system regulator; Is phosphorylated by SsrA; is involved in the expression of the virulence genes of Salmonella pathogenicity island-2; Derived by automated computational analysis using gene prediction method: Protein Homology*	0.856
spiC	ssrA	CY43_07090	CY43_07085	*SPI-2 type III secretion system protein SpiC; Involved in macrophage infection; inhibits phagosome-lysosome fusion and cellular trafficking; Derived by automated computational analysis using gene prediction method: Protein Homology*	*Histidine kinase; Phosphorylates the response regulator SsrB; is involved in the expression of the virulence genes of Salmonella pathogenicity island-2; Derived by automated computational analysis using gene prediction method: Protein Homology*	0.902
spiC	sseF	CY43_07090	CY43_07145	*SPI-2 type III secretion system protein SpiC; Involved in macrophage infection; inhibits phagosome-lysosome fusion and cellular trafficking; Derived by automated computational analysis using gene prediction method: Protein Homology*	*Pathogenicity island 2 effector protein SseF; With SseG is involved in the aggregation of the host endosomes; Derived by automated computational analysis using gene prediction method: Protein Homology*	0.803
spiC	sseE	CY43_07090	CY43_07135	*SPI-2 type III secretion system protein SpiC; Involved in macrophage infection; inhibits phagosome-lysosome fusion and cellular trafficking; Derived by automated computational analysis using gene prediction method: Protein Homology*	*Pathogenicity island 2 effector protein SseE; Derived by automated computational analysis using gene prediction method: Protein Homology*	0.844
spiC	sseD	CY43_07090	CY43_07130	*SPI-2 type III secretion system protein SpiC; Involved in macrophage infection; inhibits phagosome-lysosome fusion and cellular trafficking; Derived by automated*	*SPI-2 type III secretion system translocon protein SseD; May be involved in the translocation of effector proteins into the host cell; Derived by automated computational*	0.863

				computational analysis using gene prediction method: Protein Homology	*analysis using gene prediction method: Protein Homology*	
spiC	sseB	CY43_07090	CY43_07115	*SPI-2 type III secretion system protein SpiC; Involved in macrophage infection; inhibits phagosome-lysosome fusion and cellular trafficking; Derived by automated computational analysis using gene prediction method: Protein Homology*	*SPI-2 type III secretion system translocon protein SseB; Necessary for the correct localization of SseC and SseD on the bacterial cell surface; Derived by automated computational analysis using gene prediction method: Protein Homology*	0.877
spiC	sscB	CY43_07090	CY43_07140	*SPI-2 type III secretion system protein SpiC; Involved in macrophage infection; inhibits phagosome-lysosome fusion and cellular trafficking; Derived by automated computational analysis using gene prediction method: Protein Homology*	*Salmonella pathogenicity island 2 protein; member of a type III secretion system involved in the survival and replication of Salmonella in a host cell; chaperone for SseF; required for the efficient replication of Salmonella in host macrophages; Derived by automated computational analysis using gene prediction method: Protein Homology*	0.703
spiC	sscA	CY43_07090	CY43_07120	*SPI-2 type III secretion system protein SpiC; Involved in macrophage infection; inhibits phagosome-lysosome fusion and cellular trafficking; Derived by automated computational analysis using gene prediction method: Protein Homology*	*CesD/SycD/LcrH family type III secretion system chaperone; May be involved in the translocation of effector proteins into the host cell; Derived by automated computational analysis using gene prediction method: Protein Homology*	0.876
spiC	ssaV	CY43_07090	CY43_07195	*SPI-2 type III secretion system protein SpiC; Involved in macrophage infection; inhibits phagosome-lysosome fusion and cellular trafficking; Derived by automated computational analysis using gene prediction method: Protein Homology*	*SPI-2 type III secretion system apparatus protein SsaV; With SsaC forms part of a protein export system across the inner and outer cell membranes; part of the Salmonella pathogenicity island 2; part of the type III secretion system; Derived by automated computational analysis using gene prediction method: Protein Homology*	0.607
spiC	ssaU	CY43_07090	CY43_07235	*SPI-2 type III secretion system protein SpiC; Involved in macrophage infection; inhibits phagosome-lysosome fusion and cellular trafficking; Derived by automated computational analysis using gene prediction method: Protein Homology*	*Member of a type III secretion system which is part of a pathogenicity island in Salmonella, Yersinia and pathogenic Escherichia coli; Derived by automated computational analysis using gene prediction method: Protein Homology*	0.662
spiC	ssaS	CY43_07090	CY43_07225	*SPI-2 type III secretion system protein SpiC; Involved in macrophage infection; inhibits*	*SPI-2 type III secretion system apparatus protein SsaS; Derived by automated*	0.691

				phagosome-lysosome fusion and cellular trafficking; Derived by automated computational analysis using gene prediction method: Protein Homology	*computational analysis using gene prediction method: Protein Homology*	
spiC	ssaR	CY43_07090	CY43_07220	*SPI-2 type III secretion system protein SpiC; Involved in macrophage infection; inhibits phagosome-lysosome fusion and cellular trafficking; Derived by automated computational analysis using gene prediction method: Protein Homology*	*SPI-2 type III secretion system export apparatus protein SsaR; Part of a set of proteins involved in the infection of eukaryotic cells; in plant pathogens involved in the hypersensitivity response; Derived by automated computational analysis using gene prediction method: Protein Homology*	0.841
spiC	ssaQ	CY43_07090	CY43_07215	*SPI-2 type III secretion system protein SpiC; Involved in macrophage infection; inhibits phagosome-lysosome fusion and cellular trafficking; Derived by automated computational analysis using gene prediction method: Protein Homology*	*SPI-2 type III secretion system apparatus protein SsaQ; Derived by automated computational analysis using gene prediction method: Protein Homology*	0.651
spiC	ssaP	CY43_07090	CY43_07210	*SPI-2 type III secretion system protein SpiC; Involved in macrophage infection; inhibits phagosome-lysosome fusion and cellular trafficking; Derived by automated computational analysis using gene prediction method: Protein Homology*	*Salmonella pathogenicity island 2 protein; member of a type III secretion system involved in the survival and replication of Salmonella in a host cell; Derived by automated computational analysis using gene prediction method: Protein Homology*	0.683
spiC	ssaO	CY43_07090	CY43_07205	*SPI-2 type III secretion system protein SpiC; Involved in macrophage infection; inhibits phagosome-lysosome fusion and cellular trafficking; Derived by automated computational analysis using gene prediction method: Protein Homology*	*Salmonella pathogenicity island 2 protein; member of a type III secretion system involved in the survival and replication of Salmonella in a host cell; Derived by automated computational analysis using gene prediction method: Protein Homology*	0.642
spiC	ssaN	CY43_07090	CY43_07200	*SPI-2 type III secretion system protein SpiC; Involved in macrophage infection; inhibits phagosome-lysosome fusion and cellular trafficking; Derived by automated computational analysis using gene prediction method: Protein Homology*	*EscN/YscN/HrcN family type III secretion system ATPase; Derived by automated computational analysis using gene prediction method: Protein Homology*	0.607
spiC	ssaI	CY43_07090	CY43_07165	*SPI-2 type III secretion system protein SpiC; Involved in macrophage infection; inhibits*	*Salmonella pathogenicity island 2 protein; member of a type III secretion system involved*	0.538

				phagosome-lysosome fusion and cellular trafficking; Derived by automated computational analysis using gene prediction method: Protein Homology	*in the survival and replication of Salmonella in a host cell; Derived by automated computational analysis using gene prediction method: Protein Homology*	
spiC	ssaG	CY43_07090	CY43_07155	*SPI-2 type III secretion system protein SpiC; Involved in macrophage infection; inhibits phagosome-lysosome fusion and cellular trafficking; Derived by automated computational analysis using gene prediction method: Protein Homology*	*EscF/YscF/HrpA family type III secretion system needle major subunit; Salmonella pathogenicity island 2 protein; member of a type III secretion system involved in the survival and replication of Salmonella in a host cell; Derived by automated computational analysis using gene prediction method: Protein Homology*	0.937
spiC	ssaE	CY43_07090	CY43_07105	*SPI-2 type III secretion system protein SpiC; Involved in macrophage infection; inhibits phagosome-lysosome fusion and cellular trafficking; Derived by automated computational analysis using gene prediction method: Protein Homology*	*Salmonella pathogenicity island 2 protein; member of a type III secretion system involved in the survival and replication of Salmonella in a host cell; involved in the secretion of SseB; Derived by automated computational analysis using gene prediction method: Protein Homology*	0.930
spiC	ssaD	CY43_07090	CY43_07100	*SPI-2 type III secretion system protein SpiC; Involved in macrophage infection; inhibits phagosome-lysosome fusion and cellular trafficking; Derived by automated computational analysis using gene prediction method: Protein Homology*	*Salmonella pathogenicity island 2 protein; member of a type III secretion system involved in the survival and replication of Salmonella in a host cell; Derived by automated computational analysis using gene prediction method: Protein Homology*	0.955
spiC	spiA	CY43_07090	CY43_07095	*SPI-2 type III secretion system protein SpiC; Involved in macrophage infection; inhibits phagosome-lysosome fusion and cellular trafficking; Derived by automated computational analysis using gene prediction method: Protein Homology*	*SPI-2 type III secretion system protein SpiA; Derived by automated computational analysis using gene prediction method: Protein Homology*	0.959
spiC	DD95_20700	CY43_07090	CY43_07230	*SPI-2 type III secretion system protein SpiC; Involved in macrophage infection; inhibits phagosome-lysosome fusion and cellular trafficking; Derived by automated computational analysis using gene prediction method: Protein Homology*	*Salmonella pathogenicity island 2 protein; member of a type III secretion system involved in the survival and replication of Salmonella in a host cell; Derived by automated computational analysis using gene prediction method: Protein Homology*	0.569

spiA	ssrB	CY43_07095	CY43_07080	*SPI-2 type III secretion system protein SpiA; Derived by automated computational analysis using gene prediction method: Protein Homology*	*Type III secretion system regulator; Is phosphorylated by SsrA; is involved in the expression of the virulence genes of Salmonella pathogenicity island-2; Derived by automated computational analysis using gene prediction method: Protein Homology*	0.806
spiA	ssrA	CY43_07095	CY43_07085	*SPI-2 type III secretion system protein SpiA; Derived by automated computational analysis using gene prediction method: Protein Homology*	*Histidine kinase; Phosphorylates the response regulator SsrB; is involved in the expression of the virulence genes of Salmonella pathogenicity island-2; Derived by automated computational analysis using gene prediction method: Protein Homology*	0.914
spiA	sseF	CY43_07095	CY43_07145	*SPI-2 type III secretion system protein SpiA; Derived by automated computational analysis using gene prediction method: Protein Homology*	*Pathogenicity island 2 effector protein SseF; With SseG is involved in the aggregation of the host endosomes; Derived by automated computational analysis using gene prediction method: Protein Homology*	0.628
spiA	sseE	CY43_07095	CY43_07135	*SPI-2 type III secretion system protein SpiA; Derived by automated computational analysis using gene prediction method: Protein Homology*	*Pathogenicity island 2 effector protein SseE; Derived by automated computational analysis using gene prediction method: Protein Homology*	0.738
spiA	sseD	CY43_07095	CY43_07130	*SPI-2 type III secretion system protein SpiA; Derived by automated computational analysis using gene prediction method: Protein Homology*	*SPI-2 type III secretion system translocon protein SseD; May be involved in the translocation of effector proteins into the host cell; Derived by automated computational analysis using gene prediction method: Protein Homology*	0.849
spiA	sseB	CY43_07095	CY43_07115	*SPI-2 type III secretion system protein SpiA; Derived by automated computational analysis using gene prediction method: Protein Homology*	*SPI-2 type III secretion system translocon protein SseB; Necessary for the correct localization of SseC and SseD on the bacterial cell surface; Derived by automated computational analysis using gene prediction method: Protein Homology*	0.859
spiA	sscB	CY43_07095	CY43_07140	*SPI-2 type III secretion system protein SpiA; Derived by automated computational analysis using gene prediction method: Protein Homology*	*Salmonella pathogenicity island 2 protein; member of a type III secretion system involved in the survival and replication of Salmonella in a host cell; chaperone for SseF; required for the efficient replication of Salmonella in host*	0.899

					macrophages; Derived by automated computational analysis using gene prediction method: Protein Homology	
spiA	sscA	CY43_07095	CY43_07120	*SPI-2 type III secretion system protein SpiA; Derived by automated computational analysis using gene prediction method: Protein Homology*	*CesD/SycD/LcrH family type III secretion system chaperone; May be involved in the translocation of effector proteins into the host cell; Derived by automated computational analysis using gene prediction method: Protein Homology*	0.976
spiA	ssaV	CY43_07095	CY43_07195	*SPI-2 type III secretion system protein SpiA; Derived by automated computational analysis using gene prediction method: Protein Homology*	*SPI-2 type III secretion system apparatus protein SsaV; With SsaC forms part of a protein export system across the inner and outer cell membranes; part of the Salmonella pathogenicity island 2; part of the type III secretion system; Derived by automated computational analysis using gene prediction method: Protein Homology*	0.807
spiA	ssaU	CY43_07095	CY43_07235	*SPI-2 type III secretion system protein SpiA; Derived by automated computational analysis using gene prediction method: Protein Homology*	*Member of a type III secretion system which is part of a pathogenicity island in Salmonella, Yersinia and pathogenic Escherichia coli; Derived by automated computational analysis using gene prediction method: Protein Homology*	0.815
spiA	ssaS	CY43_07095	CY43_07225	*SPI-2 type III secretion system protein SpiA; Derived by automated computational analysis using gene prediction method: Protein Homology*	*SPI-2 type III secretion system apparatus protein SsaS; Derived by automated computational analysis using gene prediction method: Protein Homology*	0.807
spiA	ssaR	CY43_07095	CY43_07220	*SPI-2 type III secretion system protein SpiA; Derived by automated computational analysis using gene prediction method: Protein Homology*	*SPI-2 type III secretion system export apparatus protein SsaR; Part of a set of proteins involved in the infection of eukaryotic cells; in plant pathogens involved in the hypersensitivity response; Derived by automated computational analysis using gene prediction method: Protein Homology*	0.839
spiA	ssaQ	CY43_07095	CY43_07215	*SPI-2 type III secretion system protein SpiA; Derived by automated computational analysis using gene prediction method: Protein Homology*	*SPI-2 type III secretion system apparatus protein SsaQ; Derived by automated computational analysis using gene prediction method: Protein Homology*	0.948

spiA	ssaP	CY43_07095	CY43_07210	*SPI-2 type III secretion system protein SpiA; Derived by automated computational analysis using gene prediction method: Protein Homology*	*Salmonella pathogenicity island 2 protein; member of a type III secretion system involved in the survival and replication of Salmonella in a host cell; Derived by automated computational analysis using gene prediction method: Protein Homology*	0.626
spiA	ssaO	CY43_07095	CY43_07205	*SPI-2 type III secretion system protein SpiA; Derived by automated computational analysis using gene prediction method: Protein Homology*	*Salmonella pathogenicity island 2 protein; member of a type III secretion system involved in the survival and replication of Salmonella in a host cell; Derived by automated computational analysis using gene prediction method: Protein Homology*	0.486
spiA	ssaN	CY43_07095	CY43_07200	*SPI-2 type III secretion system protein SpiA; Derived by automated computational analysis using gene prediction method: Protein Homology*	*EscN/YscN/HrcN family type III secretion system ATPase; Derived by automated computational analysis using gene prediction method: Protein Homology*	0.789
spiA	ssaI	CY43_07095	CY43_07165	*SPI-2 type III secretion system protein SpiA; Derived by automated computational analysis using gene prediction method: Protein Homology*	*Salmonella pathogenicity island 2 protein; member of a type III secretion system involved in the survival and replication of Salmonella in a host cell; Derived by automated computational analysis using gene prediction method: Protein Homology*	0.537
spiA	ssaG	CY43_07095	CY43_07155	*SPI-2 type III secretion system protein SpiA; Derived by automated computational analysis using gene prediction method: Protein Homology*	*EscF/YscF/HrpA family type III secretion system needle major subunit; Salmonella pathogenicity island 2 protein; member of a type III secretion system involved in the survival and replication of Salmonella in a host cell; Derived by automated computational analysis using gene prediction method: Protein Homology*	0.775
spiA	ssaE	CY43_07095	CY43_07105	*SPI-2 type III secretion system protein SpiA; Derived by automated computational analysis using gene prediction method: Protein Homology*	*Salmonella pathogenicity island 2 protein; member of a type III secretion system involved in the survival and replication of Salmonella in a host cell; involved in the secretion of SseB; Derived by automated computational analysis using gene prediction method: Protein Homology*	0.927

spiA	ssaD	CY43_07095	CY43_07100	*SPI-2 type III secretion system protein SpiA; Derived by automated computational analysis using gene prediction method: Protein Homology*	*Salmonella pathogenicity island 2 protein; member of a type III secretion system involved in the survival and replication of Salmonella in a host cell; Derived by automated computational analysis using gene prediction method: Protein Homology*	0.988
spiA	spiC	CY43_07095	CY43_07090	*SPI-2 type III secretion system protein SpiA; Derived by automated computational analysis using gene prediction method: Protein Homology*	*SPI-2 type III secretion system protein SpiC; Involved in macrophage infection; inhibits phagosome-lysosome fusion and cellular trafficking; Derived by automated computational analysis using gene prediction method: Protein Homology*	0.959
spiA	DD95_20700	CY43_07095	CY43_07230	*SPI-2 type III secretion system protein SpiA; Derived by automated computational analysis using gene prediction method: Protein Homology*	*Salmonella pathogenicity island 2 protein; member of a type III secretion system involved in the survival and replication of Salmonella in a host cell; Derived by automated computational analysis using gene prediction method: Protein Homology*	0.905
DD95_20875	ssrA	CY43_07050	CY43_07085	*Histidine kinase; Derived by automated computational analysis using gene prediction method: Protein Homology*	*Histidine kinase; Phosphorylates the response regulator SsrB; is involved in the expression of the virulence genes of Salmonella pathogenicity island-2; Derived by automated computational analysis using gene prediction method: Protein Homology*	0.798
DD95_20875	DD95_20870	CY43_07050	CY43_07055	*Histidine kinase; Derived by automated computational analysis using gene prediction method: Protein Homology*	*Tetrathionate response regulatory protein TtrR; Derived by automated computational analysis using gene prediction method: Protein Homology*	0.997
DD95_20870	DD95_20875	CY43_07055	CY43_07050	*Tetrathionate response regulatory protein TtrR; Derived by automated computational analysis using gene prediction method: Protein Homology*	*Histidine kinase; Derived by automated computational analysis using gene prediction method: Protein Homology*	0.997
DD95_20700	ssrB	CY43_07230	CY43_07080	*Salmonella pathogenicity island 2 protein; member of a type III secretion system involved in the survival and replication of Salmonella in a host cell; Derived by automated computational analysis using gene prediction method: Protein Homology*	*Type III secretion system regulator; Is phosphorylated by SsrA; is involved in the expression of the virulence genes of Salmonella pathogenicity island-2; Derived by automated computational analysis using gene prediction method: Protein Homology*	0.588

DD95_20700	ssrA	CY43_07230	CY43_07085	*Salmonella pathogenicity island 2 protein; member of a type III secretion system involved in the survival and replication of Salmonella in a host cell; Derived by automated computational analysis using gene prediction method: Protein Homology*	*Histidine kinase; Phosphorylates the response regulator SsrB; is involved in the expression of the virulence genes of Salmonella pathogenicity island-2; Derived by automated computational analysis using gene prediction method: Protein Homology*	0.703
DD95_20700	sseF	CY43_07230	CY43_07145	*Salmonella pathogenicity island 2 protein; member of a type III secretion system involved in the survival and replication of Salmonella in a host cell; Derived by automated computational analysis using gene prediction method: Protein Homology*	*Pathogenicity island 2 effector protein SseF; With SseG is involved in the aggregation of the host endosomes; Derived by automated computational analysis using gene prediction method: Protein Homology*	0.544
DD95_20700	sseE	CY43_07230	CY43_07135	*Salmonella pathogenicity island 2 protein; member of a type III secretion system involved in the survival and replication of Salmonella in a host cell; Derived by automated computational analysis using gene prediction method: Protein Homology*	*Pathogenicity island 2 effector protein SseE; Derived by automated computational analysis using gene prediction method: Protein Homology*	0.556
DD95_20700	sseD	CY43_07230	CY43_07130	*Salmonella pathogenicity island 2 protein; member of a type III secretion system involved in the survival and replication of Salmonella in a host cell; Derived by automated computational analysis using gene prediction method: Protein Homology*	*SPI-2 type III secretion system translocon protein SseD; May be involved in the translocation of effector proteins into the host cell; Derived by automated computational analysis using gene prediction method: Protein Homology*	0.656
DD95_20700	sseB	CY43_07230	CY43_07115	*Salmonella pathogenicity island 2 protein; member of a type III secretion system involved in the survival and replication of Salmonella in a host cell; Derived by automated computational analysis using gene prediction method: Protein Homology*	*SPI-2 type III secretion system translocon protein SseB; Necessary for the correct localization of SseC and SseD on the bacterial cell surface; Derived by automated computational analysis using gene prediction method: Protein Homology*	0.651
DD95_20700	sscB	CY43_07230	CY43_07140	*Salmonella pathogenicity island 2 protein; member of a type III secretion system involved in the survival and replication of Salmonella in a host cell; Derived by automated computational analysis using gene prediction method: Protein Homology*	*Salmonella pathogenicity island 2 protein; member of a type III secretion system involved in the survival and replication of Salmonella in a host cell; chaperone for SseF; required for the efficient replication of Salmonella in host macrophages; Derived by automated computational analysis using gene prediction method: Protein Homology*	0.883

DD95_20700	sscA	CY43_07230	CY43_07120	*Salmonella pathogenicity island 2 protein; member of a type III secretion system involved in the survival and replication of Salmonella in a host cell; Derived by automated computational analysis using gene prediction method: Protein Homology*	*CesD/SycD/LcrH family type III secretion system chaperone; May be involved in the translocation of effector proteins into the host cell; Derived by automated computational analysis using gene prediction method: Protein Homology*	0.906
DD95_20700	ssaV	CY43_07230	CY43_07195	*Salmonella pathogenicity island 2 protein; member of a type III secretion system involved in the survival and replication of Salmonella in a host cell; Derived by automated computational analysis using gene prediction method: Protein Homology*	*SPI-2 type III secretion system apparatus protein SsaV; With SsaC forms part of a protein export system across the inner and outer cell membranes; part of the Salmonella pathogenicity island 2; part of the type III secretion system; Derived by automated computational analysis using gene prediction method: Protein Homology*	0.962
DD95_20700	ssaU	CY43_07230	CY43_07235	*Salmonella pathogenicity island 2 protein; member of a type III secretion system involved in the survival and replication of Salmonella in a host cell; Derived by automated computational analysis using gene prediction method: Protein Homology*	*Member of a type III secretion system which is part of a pathogenicity island in Salmonella, Yersinia and pathogenic Escherichia coli; Derived by automated computational analysis using gene prediction method: Protein Homology*	0.991
DD95_20700	ssaS	CY43_07230	CY43_07225	*Salmonella pathogenicity island 2 protein; member of a type III secretion system involved in the survival and replication of Salmonella in a host cell; Derived by automated computational analysis using gene prediction method: Protein Homology*	*SPI-2 type III secretion system apparatus protein SsaS; Derived by automated computational analysis using gene prediction method: Protein Homology*	0.983
DD95_20700	ssaR	CY43_07230	CY43_07220	*Salmonella pathogenicity island 2 protein; member of a type III secretion system involved in the survival and replication of Salmonella in a host cell; Derived by automated computational analysis using gene prediction method: Protein Homology*	*SPI-2 type III secretion system export apparatus protein SsaR; Part of a set of proteins involved in the infection of eukaryotic cells; in plant pathogens involved in the hypersensitivity response; Derived by automated computational analysis using gene prediction method: Protein Homology*	0.990
DD95_20700	ssaQ	CY43_07230	CY43_07215	*Salmonella pathogenicity island 2 protein; member of a type III secretion system involved in the survival and replication of Salmonella in a host cell; Derived by automated*	*SPI-2 type III secretion system apparatus protein SsaQ; Derived by automated computational analysis using gene prediction method: Protein Homology*	0.919

				computational analysis using gene prediction method: Protein Homology		
DD95_20700	ssaP	CY43_07230	CY43_07210	*Salmonella pathogenicity island 2 protein; member of a type III secretion system involved in the survival and replication of Salmonella in a host cell; Derived by automated computational analysis using gene prediction method: Protein Homology*	*Salmonella pathogenicity island 2 protein; member of a type III secretion system involved in the survival and replication of Salmonella in a host cell; Derived by automated computational analysis using gene prediction method: Protein Homology*	0.762
DD95_20700	ssaO	CY43_07230	CY43_07205	*Salmonella pathogenicity island 2 protein; member of a type III secretion system involved in the survival and replication of Salmonella in a host cell; Derived by automated computational analysis using gene prediction method: Protein Homology*	*Salmonella pathogenicity island 2 protein; member of a type III secretion system involved in the survival and replication of Salmonella in a host cell; Derived by automated computational analysis using gene prediction method: Protein Homology*	0.763
DD95_20700	ssaN	CY43_07230	CY43_07200	*Salmonella pathogenicity island 2 protein; member of a type III secretion system involved in the survival and replication of Salmonella in a host cell; Derived by automated computational analysis using gene prediction method: Protein Homology*	*EscN/YscN/HrcN family type III secretion system ATPase; Derived by automated computational analysis using gene prediction method: Protein Homology*	0.945
DD95_20700	ssaI	CY43_07230	CY43_07165	*Salmonella pathogenicity island 2 protein; member of a type III secretion system involved in the survival and replication of Salmonella in a host cell; Derived by automated computational analysis using gene prediction method: Protein Homology*	*Salmonella pathogenicity island 2 protein; member of a type III secretion system involved in the survival and replication of Salmonella in a host cell; Derived by automated computational analysis using gene prediction method: Protein Homology*	0.746
DD95_20700	ssaG	CY43_07230	CY43_07155	*Salmonella pathogenicity island 2 protein; member of a type III secretion system involved in the survival and replication of Salmonella in a host cell; Derived by automated computational analysis using gene prediction method: Protein Homology*	*EscF/YscF/HrpA family type III secretion system needle major subunit; Salmonella pathogenicity island 2 protein; member of a type III secretion system involved in the survival and replication of Salmonella in a host cell; Derived by automated computational analysis using gene prediction method: Protein Homology*	0.746
DD95_20700	ssaE	CY43_07230	CY43_07105	*Salmonella pathogenicity island 2 protein; member of a type III secretion system involved in the survival and replication of Salmonella in a host cell; Derived by automated*	*Salmonella pathogenicity island 2 protein; member of a type III secretion system involved in the survival and replication of Salmonella in a host cell; involved in the secretion of SseB;*	0.610

				computational analysis using gene prediction method: Protein Homology	*Derived by automated computational analysis using gene prediction method: Protein Homology*	
DD95_20700	ssaD	CY43_07230	CY43_07100	*Salmonella pathogenicity island 2 protein; member of a type III secretion system involved in the survival and replication of Salmonella in a host cell; Derived by automated computational analysis using gene prediction method: Protein Homology*	*Salmonella pathogenicity island 2 protein; member of a type III secretion system involved in the survival and replication of Salmonella in a host cell; Derived by automated computational analysis using gene prediction method: Protein Homology*	0.892
DD95_20700	spiC	CY43_07230	CY43_07090	*Salmonella pathogenicity island 2 protein; member of a type III secretion system involved in the survival and replication of Salmonella in a host cell; Derived by automated computational analysis using gene prediction method: Protein Homology*	*SPI-2 type III secretion system protein SpiC; Involved in macrophage infection; inhibits phagosome-lysosome fusion and cellular trafficking; Derived by automated computational analysis using gene prediction method: Protein Homology*	0.569
DD95_20700	spiA	CY43_07230	CY43_07095	*Salmonella pathogenicity island 2 protein; member of a type III secretion system involved in the survival and replication of Salmonella in a host cell; Derived by automated computational analysis using gene prediction method: Protein Homology*	*SPI-2 type III secretion system protein SpiA; Derived by automated computational analysis using gene prediction method: Protein Homology*	0.905
