# Supplementary material for: Genome analysis and virulence gene expression profile of a multi drug resistant Salmonella enterica serovar Typhimurium ms202
Source: Gut Pathog. 2022 Jun 28;14:28. doi: 10.1186/s13099-022-00498-w (PMC9237969; doi:10.1186/s13099-022-00498-w)
Supplement: Supplementary file 6 — Additional file 6: Table S6. The interaction networks and the co-expression details of the SPI-1 cluster proteins in S. enterica Typhimurium ms202 [file 13099_2022_498_MOESM6_ESM.pdf]

Table S6: The interaction networks and the co-expression details of the SPI1 cluster proteins in *S. enterica* Typhimurium ms202.

node1	node2	node1 accession	node2 accession	node1 annotation	node2 annotation	score
sptP	sprB	CY43_15030	CY43_14970	*SPI-1 type III secretion system effector GTPase-activating protein SptP; GTP-activating protein/tyrosine phosphatase; facilitates bacterial survival in host cells; Derived by automated computational analysis using gene prediction method: Protein Homology*	*Transcriptional regulator SprB; Activates Salmonella pathogenicity island 4 genes and weakly represses Salmonella pathogenicity island 1 genes; Derived by automated computational analysis using gene prediction method: Protein Homology*	0.440
sptP	spaO	CY43_15030	CY43_15095	*SPI-1 type III secretion system effector GTPase-activating protein SptP; GTP-activating protein/tyrosine phosphatase; facilitates bacterial survival in host cells; Derived by automated computational analysis using gene prediction method: Protein Homology*	*Involved in a secretory pathway responsible for the surface presentation of determinants needed for the entry of Salmonella species into mammalian cells; Derived by automated computational analysis using gene prediction method: Protein Homology*	0.912
sptP	sipD	CY43_15030	CY43_15055	*SPI-1 type III secretion system effector GTPase-activating protein SptP; GTP-activating protein/tyrosine phosphatase; facilitates bacterial survival in host cells; Derived by automated computational analysis using gene prediction method: Protein Homology*	*Involved in the transport of effector protein by the type III secretion system of Salmonella pathogenicity island 1; Derived by automated computational analysis using gene prediction method: Protein Homology*	0.956
sptP	sipC	CY43_15030	CY43_15060	*SPI-1 type III secretion system effector GTPase-activating protein SptP; GTP-activating protein/tyrosine phosphatase; facilitates bacterial survival in host cells; Derived by automated computational analysis using gene prediction method: Protein Homology*	*SPI-1 type III secretion system needle tip complex protein SipC; Cell invasion protein C; required for entry of the bacteria into the host cell; binds to actin; Derived by automated computational analysis using gene prediction method: Protein Homology*	0.980
sptP	sipB	CY43_15030	CY43_15065	*SPI-1 type III secretion system effector GTPase-activating protein SptP; GTP-activating protein/tyrosine phosphatase; facilitates bacterial survival in host cells; Derived by automated computational analysis using gene prediction method: Protein Homology*	*SPI-1 type III secretion system needle tip complex protein SipB; Cell invasion protein B; required for entry of the bacteria into the host cell; induces apoptosis in macrophages; Derived by automated computational analysis using gene prediction method: Protein Homology*	0.987
sptP	sipA	CY43_15030	CY43_15050	*SPI-1 type III secretion system effector GTPase-activating protein SptP; GTP-activating protein/tyrosine phosphatase; facilitates bacterial survival in host cells; Derived by*	*SPI-1 type III secretion system effector SipA; Cell invasion protein A; actin-binding; required for entry of the Salmonella into the host cell; Derived by*	0.988

				automated computational analysis using gene prediction method: Protein Homology	*automated computational analysis using gene prediction method: Protein Homology*	
sptP	sicP	CY43_15030	CY43_15035	*SPI-1 type III secretion system effector GTPase-activating protein SptP; GTP-activating protein/tyrosine phosphatase; facilitates bacterial survival in host cells; Derived by automated computational analysis using gene prediction method: Protein Homology*	*Chaperone protein SicP; Type III secretion system chaperone; Derived by automated computational analysis using gene prediction method: Protein Homology*	0.998
sptP	prgK	CY43_15030	CY43_14995	*SPI-1 type III secretion system effector GTPase-activating protein SptP; GTP-activating protein/tyrosine phosphatase; facilitates bacterial survival in host cells; Derived by automated computational analysis using gene prediction method: Protein Homology*	*Lipoprotein; Derived by automated computational analysis using gene prediction method: Protein Homology*	0.750
sptP	prgJ	CY43_15030	CY43_15000	*SPI-1 type III secretion system effector GTPase-activating protein SptP; GTP-activating protein/tyrosine phosphatase; facilitates bacterial survival in host cells; Derived by automated computational analysis using gene prediction method: Protein Homology*	*Type III secretion system needle complex protein PrgJ; May be involved in capping the needle substructure; Derived by automated computational analysis using gene prediction method: Protein Homology*	0.460
sptP	prgI	CY43_15030	CY43_15005	*SPI-1 type III secretion system effector GTPase-activating protein SptP; GTP-activating protein/tyrosine phosphatase; facilitates bacterial survival in host cells; Derived by automated computational analysis using gene prediction method: Protein Homology*	*Type III secretion system needle complex protein PrgI; With InvG, PrgH, and Prg K makes up the membrane spanning needle complex; Derived by automated computational analysis using gene prediction method: Protein Homology*	0.757
sptP	prgH	CY43_15030	CY43_15010	*SPI-1 type III secretion system effector GTPase-activating protein SptP; GTP-activating protein/tyrosine phosphatase; facilitates bacterial survival in host cells; Derived by automated computational analysis using gene prediction method: Protein Homology*	*Type III secretion system inner membrane ring protein PrgH; With InvG, PrgI, and Prg K makes up the membrane spanning needle complex; Derived by automated computational analysis using gene prediction method: Protein Homology*	0.938
sptP	invG	CY43_15030	CY43_15130	*SPI-1 type III secretion system effector GTPase-activating protein SptP; GTP-activating protein/tyrosine phosphatase; facilitates bacterial survival in host cells; Derived by automated computational analysis using gene prediction method: Protein Homology*	*Type III secretion system outer membrane ring protein InvG; May be involved in the export or assemby of proteins involved in the entry of Salmonella into host cells; Derived by automated computational analysis using gene prediction method: Protein Homology*	0.840

sptP	iagB	CY43_15030	CY43_15025	*SPI-1 type III secretion system effector GTPase-activating protein SptP; GTP-activating protein/tyrosine phosphatase; facilitates bacterial survival in host cells; Derived by automated computational analysis using gene prediction method: Protein Homology*	*SPI-1 type III secretion system invasion protein IagB; Derived by automated computational analysis using gene prediction method: Protein Homology*	0.495
sptP	hilA	CY43_15030	CY43_15020	*SPI-1 type III secretion system effector GTPase-activating protein SptP; GTP-activating protein/tyrosine phosphatase; facilitates bacterial survival in host cells; Derived by automated computational analysis using gene prediction method: Protein Homology*	*Invasion protein regulator; IagA; HilA; activates the expression of invasion genes and activates the expression of prgHIJK which is part of the pathogenicity island 1 type III secretion system; Derived by automated computational analysis using gene prediction method: Protein Homology*	0.556
sptP	DD95_24045	CY43_15030	CY43_14490	*SPI-1 type III secretion system effector GTPase-activating protein SptP; GTP-activating protein/tyrosine phosphatase; facilitates bacterial survival in host cells; Derived by automated computational analysis using gene prediction method: Protein Homology*	*Flagellin FliC; Structural flagella protein; individual Salmonella serotypes usually alternate between the production of 2 antigenic forms of flagella, termed phase 1 and phase 2, each specified by separate structural genes; Derived by automated computational analysis using gene prediction method: Protein Homology*	0.557
sptP	DD95_19955	CY43_15030	CY43_10320	*SPI-1 type III secretion system effector GTPase-activating protein SptP; GTP-activating protein/tyrosine phosphatase; facilitates bacterial survival in host cells; Derived by automated computational analysis using gene prediction method: Protein Homology*	*Flagellin; Structural flagella protein; individual Salmonella serotypes usually alternate between the production of 2 antigenic forms of flagella, termed phase 1 and phase 2, each specified by separate structural genes; Derived by automated computational analysis using gene prediction method: Protein Homology*	0.558
sprB	sptP	CY43_14970	CY43_15030	*Transcriptional regulator SprB; Activates Salmonella pathogenicity island 4 genes and weakly represses Salmonella pathogenicity island 1 genes; Derived by automated computational analysis using gene prediction method: Protein Homology*	*SPI-1 type III secretion system effector GTPase-activating protein SptP; GTP-activating protein/tyrosine phosphatase; facilitates bacterial survival in host cells; Derived by automated computational analysis using gene prediction method: Protein Homology*	0.440
sprB	spaO	CY43_14970	CY43_15095	*Transcriptional regulator SprB; Activates Salmonella pathogenicity island 4 genes and weakly represses Salmonella pathogenicity island 1 genes; Derived by automated computational analysis using gene prediction method: Protein Homology*	*Involved in a secretory pathway responsible for the surface presentation of determinants needed for the entry of Salmonella species into mammalian cells; Derived by automated computational analysis using gene prediction method: Protein Homology*	0.403

sprB	sitD	CY43_14970	CY43_14960	*Transcriptional regulator SprB; Activates Salmonella pathogenicity island 4 genes and weakly represses Salmonella pathogenicity island 1 genes; Derived by automated computational analysis using gene prediction method: Protein Homology*	*Metal ABC transporter permease; Derived by automated computational analysis using gene prediction method: Protein Homology*	0.599
sprB	sitC	CY43_14970	CY43_14955	*Transcriptional regulator SprB; Activates Salmonella pathogenicity island 4 genes and weakly represses Salmonella pathogenicity island 1 genes; Derived by automated computational analysis using gene prediction method: Protein Homology*	*Metal ABC transporter permease; Derived by automated computational analysis using gene prediction method: Protein Homology*	0.559
sprB	prgK	CY43_14970	CY43_14995	*Transcriptional regulator SprB; Activates Salmonella pathogenicity island 4 genes and weakly represses Salmonella pathogenicity island 1 genes; Derived by automated computational analysis using gene prediction method: Protein Homology*	*Lipoprotein; Derived by automated computational analysis using gene prediction method: Protein Homology*	0.434
sprB	prgI	CY43_14970	CY43_15005	*Transcriptional regulator SprB; Activates Salmonella pathogenicity island 4 genes and weakly represses Salmonella pathogenicity island 1 genes; Derived by automated computational analysis using gene prediction method: Protein Homology*	*Type III secretion system needle complex protein PrgI; With InvG, PrgH, and Prg K makes up the membrane spanning needle complex; Derived by automated computational analysis using gene prediction method: Protein Homology*	0.494
sprB	prgH	CY43_14970	CY43_15010	*Transcriptional regulator SprB; Activates Salmonella pathogenicity island 4 genes and weakly represses Salmonella pathogenicity island 1 genes; Derived by automated computational analysis using gene prediction method: Protein Homology*	*Type III secretion system inner membrane ring protein PrgH; With InvG, PrgI, and Prg K makes up the membrane spanning needle complex; Derived by automated computational analysis using gene prediction method: Protein Homology*	0.483
sprB	iagB	CY43_14970	CY43_15025	*Transcriptional regulator SprB; Activates Salmonella pathogenicity island 4 genes and weakly represses Salmonella pathogenicity island 1 genes; Derived by automated computational analysis using gene prediction method: Protein Homology*	*SPI-1 type III secretion system invasion protein IagB; Derived by automated computational analysis using gene prediction method: Protein Homology*	0.639
spaQ	spaO	CY43_15085	CY43_15095	*SPI-1 type III secretion system export apparatus protein SpaQ; Involved in the surface*	*Involved in a secretory pathway responsible for the surface presentation of determinants needed for the*	0.987

				presentation of antigens needed for the invasion of Salmonella into host cells; Derived by automated computational analysis using gene prediction method: Protein Homology	*entry of Salmonella species into mammalian cells; Derived by automated computational analysis using gene prediction method: Protein Homology*	
spaQ	sipD	CY43_15085	CY43_15055	*SPI-1 type III secretion system export apparatus protein SpaQ; Involved in the surface presentation of antigens needed for the invasion of Salmonella into host cells; Derived by automated computational analysis using gene prediction method: Protein Homology*	*Involved in the transport of effector protein by the type III secretion system of Salmonella pathogenicity island 1; Derived by automated computational analysis using gene prediction method: Protein Homology*	0.554
spaQ	sipC	CY43_15085	CY43_15060	*SPI-1 type III secretion system export apparatus protein SpaQ; Involved in the surface presentation of antigens needed for the invasion of Salmonella into host cells; Derived by automated computational analysis using gene prediction method: Protein Homology*	*SPI-1 type III secretion system needle tip complex protein SipC; Cell invasion protein C; required for entry of the bacteria into the host cell; binds to actin; Derived by automated computational analysis using gene prediction method: Protein Homology*	0.574
spaQ	sipB	CY43_15085	CY43_15065	*SPI-1 type III secretion system export apparatus protein SpaQ; Involved in the surface presentation of antigens needed for the invasion of Salmonella into host cells; Derived by automated computational analysis using gene prediction method: Protein Homology*	*SPI-1 type III secretion system needle tip complex protein SipB; Cell invasion protein B; required for entry of the bacteria into the host cell; induces apoptosis in macrophages; Derived by automated computational analysis using gene prediction method: Protein Homology*	0.891
spaQ	sipA	CY43_15085	CY43_15050	*SPI-1 type III secretion system export apparatus protein SpaQ; Involved in the surface presentation of antigens needed for the invasion of Salmonella into host cells; Derived by automated computational analysis using gene prediction method: Protein Homology*	*SPI-1 type III secretion system effector SipA; Cell invasion protein A; actin-binding; required for entry of the Salmonella into the host cell; Derived by automated computational analysis using gene prediction method: Protein Homology*	0.433
spaQ	sicP	CY43_15085	CY43_15035	*SPI-1 type III secretion system export apparatus protein SpaQ; Involved in the surface presentation of antigens needed for the invasion of Salmonella into host cells; Derived by automated computational analysis using gene prediction method: Protein Homology*	*Chaperone protein SicP; Type III secretion system chaperone; Derived by automated computational analysis using gene prediction method: Protein Homology*	0.499
spaQ	prgK	CY43_15085	CY43_14995	*SPI-1 type III secretion system export apparatus protein SpaQ; Involved in the surface presentation of antigens needed for the invasion of Salmonella into host cells; Derived by*	*Lipoprotein; Derived by automated computational analysis using gene prediction method: Protein Homology*	0.957

				automated computational analysis using gene prediction method: Protein Homology		
spaQ	prgJ	CY43_15085	CY43_15000	*SPI-1 type III secretion system export apparatus protein SpaQ; Involved in the surface presentation of antigens needed for the invasion of Salmonella into host cells; Derived by automated computational analysis using gene prediction method: Protein Homology*	*Type III secretion system needle complex protein PrgJ; May be involved in capping the needle substructure; Derived by automated computational analysis using gene prediction method: Protein Homology*	0.775
spaQ	prgI	CY43_15085	CY43_15005	*SPI-1 type III secretion system export apparatus protein SpaQ; Involved in the surface presentation of antigens needed for the invasion of Salmonella into host cells; Derived by automated computational analysis using gene prediction method: Protein Homology*	*Type III secretion system needle complex protein PrgI; With InvG, PrgH, and Prg K makes up the membrane spanning needle complex; Derived by automated computational analysis using gene prediction method: Protein Homology*	0.556
spaQ	prgH	CY43_15085	CY43_15010	*SPI-1 type III secretion system export apparatus protein SpaQ; Involved in the surface presentation of antigens needed for the invasion of Salmonella into host cells; Derived by automated computational analysis using gene prediction method: Protein Homology*	*Type III secretion system inner membrane ring protein PrgH; With InvG, PrgI, and Prg K makes up the membrane spanning needle complex; Derived by automated computational analysis using gene prediction method: Protein Homology*	0.863
spaQ	invG	CY43_15085	CY43_15130	*SPI-1 type III secretion system export apparatus protein SpaQ; Involved in the surface presentation of antigens needed for the invasion of Salmonella into host cells; Derived by automated computational analysis using gene prediction method: Protein Homology*	*Type III secretion system outer membrane ring protein InvG; May be involved in the export or assemby of proteins involved in the entry of Salmonella into host cells; Derived by automated computational analysis using gene prediction method: Protein Homology*	0.985
spaQ	iagB	CY43_15085	CY43_15025	*SPI-1 type III secretion system export apparatus protein SpaQ; Involved in the surface presentation of antigens needed for the invasion of Salmonella into host cells; Derived by automated computational analysis using gene prediction method: Protein Homology*	*SPI-1 type III secretion system invasion protein IagB; Derived by automated computational analysis using gene prediction method: Protein Homology*	0.650
spaO	sptP	CY43_15095	CY43_15030	*Involved in a secretory pathway responsible for the surface presentation of determinants needed for the entry of Salmonella species into mammalian cells; Derived by automated computational analysis using gene prediction method: Protein Homology*	*SPI-1 type III secretion system effector GTPase-activating protein SptP; GTP-activating protein/tyrosine phosphatase; facilitates bacterial survival in host cells; Derived by automated computational analysis using gene prediction method: Protein Homology*	0.912

spaO	sprB	CY43_15095	CY43_14970	*Involved in a secretory pathway responsible for the surface presentation of determinants needed for the entry of Salmonella species into mammalian cells; Derived by automated computational analysis using gene prediction method: Protein Homology*	*Transcriptional regulator SprB; Activates Salmonella pathogenicity island 4 genes and weakly represses Salmonella pathogenicity island 1 genes; Derived by automated computational analysis using gene prediction method: Protein Homology*	0.403
spaO	spaQ	CY43_15095	CY43_15085	*Involved in a secretory pathway responsible for the surface presentation of determinants needed for the entry of Salmonella species into mammalian cells; Derived by automated computational analysis using gene prediction method: Protein Homology*	*SPI-1 type III secretion system export apparatus protein SpaQ; Involved in the surface presentation of antigens needed for the invasion of Salmonella into host cells; Derived by automated computational analysis using gene prediction method: Protein Homology*	0.987
spaO	sipD	CY43_15095	CY43_15055	*Involved in a secretory pathway responsible for the surface presentation of determinants needed for the entry of Salmonella species into mammalian cells; Derived by automated computational analysis using gene prediction method: Protein Homology*	*Involved in the transport of effector protein by the type III secretion system of Salmonella pathogenicity island 1; Derived by automated computational analysis using gene prediction method: Protein Homology*	0.844
spaO	sipC	CY43_15095	CY43_15060	*Involved in a secretory pathway responsible for the surface presentation of determinants needed for the entry of Salmonella species into mammalian cells; Derived by automated computational analysis using gene prediction method: Protein Homology*	*SPI-1 type III secretion system needle tip complex protein SipC; Cell invasion protein C; required for entry of the bacteria into the host cell; binds to actin; Derived by automated computational analysis using gene prediction method: Protein Homology*	0.860
spaO	sipB	CY43_15095	CY43_15065	*Involved in a secretory pathway responsible for the surface presentation of determinants needed for the entry of Salmonella species into mammalian cells; Derived by automated computational analysis using gene prediction method: Protein Homology*	*SPI-1 type III secretion system needle tip complex protein SipB; Cell invasion protein B; required for entry of the bacteria into the host cell; induces apoptosis in macrophages; Derived by automated computational analysis using gene prediction method: Protein Homology*	0.982
spaO	sipA	CY43_15095	CY43_15050	*Involved in a secretory pathway responsible for the surface presentation of determinants needed for the entry of Salmonella species into mammalian cells; Derived by automated computational analysis using gene prediction method: Protein Homology*	*SPI-1 type III secretion system effector SipA; Cell invasion protein A; actin-binding; required for entry of the Salmonella into the host cell; Derived by automated computational analysis using gene prediction method: Protein Homology*	0.803
spaO	sicP	CY43_15095	CY43_15035	*Involved in a secretory pathway responsible for the surface presentation of determinants needed*	*Chaperone protein SicP; Type III secretion system chaperone; Derived by automated computational*	0.752

				for the entry of Salmonella species into mammalian cells; Derived by automated computational analysis using gene prediction method: Protein Homology	*analysis using gene prediction method: Protein Homology*	
spaO	prgK	CY43_15095	CY43_14995	*Involved in a secretory pathway responsible for the surface presentation of determinants needed for the entry of Salmonella species into mammalian cells; Derived by automated computational analysis using gene prediction method: Protein Homology*	*Lipoprotein; Derived by automated computational analysis using gene prediction method: Protein Homology*	0.960
spaO	prgJ	CY43_15095	CY43_15000	*Involved in a secretory pathway responsible for the surface presentation of determinants needed for the entry of Salmonella species into mammalian cells; Derived by automated computational analysis using gene prediction method: Protein Homology*	*Type III secretion system needle complex protein PrgJ; May be involved in capping the needle substructure; Derived by automated computational analysis using gene prediction method: Protein Homology*	0.668
spaO	prgI	CY43_15095	CY43_15005	*Involved in a secretory pathway responsible for the surface presentation of determinants needed for the entry of Salmonella species into mammalian cells; Derived by automated computational analysis using gene prediction method: Protein Homology*	*Type III secretion system needle complex protein PrgI; With InvG, PrgH, and Prg K makes up the membrane spanning needle complex; Derived by automated computational analysis using gene prediction method: Protein Homology*	0.810
spaO	prgH	CY43_15095	CY43_15010	*Involved in a secretory pathway responsible for the surface presentation of determinants needed for the entry of Salmonella species into mammalian cells; Derived by automated computational analysis using gene prediction method: Protein Homology*	*Type III secretion system inner membrane ring protein PrgH; With InvG, PrgI, and Prg K makes up the membrane spanning needle complex; Derived by automated computational analysis using gene prediction method: Protein Homology*	0.938
spaO	invG	CY43_15095	CY43_15130	*Involved in a secretory pathway responsible for the surface presentation of determinants needed for the entry of Salmonella species into mammalian cells; Derived by automated computational analysis using gene prediction method: Protein Homology*	*Type III secretion system outer membrane ring protein InvG; May be involved in the export or assemby of proteins involved in the entry of Salmonella into host cells; Derived by automated computational analysis using gene prediction method: Protein Homology*	0.974
paO	iagB	CY43_15095	CY43_15025	*Involved in a secretory pathway responsible for the surface presentation of determinants needed for the entry of Salmonella species into mammalian cells; Derived by automated*	*SPI-1 type III secretion system invasion protein IagB; Derived by automated computational analysis using gene prediction method: Protein Homology*	0.747

				computational analysis using gene prediction method: Protein Homology		
spaO	hilA	CY43_15095	CY43_15020	*Involved in a secretory pathway responsible for the surface presentation of determinants needed for the entry of Salmonella species into mammalian cells; Derived by automated computational analysis using gene prediction method: Protein Homology*	*Invasion protein regulator; IagA; HilA; activates the expression of invasion genes and activates the expression of prgHIJK which is part of the pathogenicity island 1 type III secretion system; Derived by automated computational analysis using gene prediction method: Protein Homology*	0.790
sitD	sprB	CY43_14960	CY43_14970	*Metal ABC transporter permease; Derived by automated computational analysis using gene prediction method: Protein Homology*	*Transcriptional regulator SprB; Activates Salmonella pathogenicity island 4 genes and weakly represses Salmonella pathogenicity island 1 genes; Derived by automated computational analysis using gene prediction method: Protein Homology*	0.599
sitD	sitC	CY43_14960	CY43_14955	*Metal ABC transporter permease; Derived by automated computational analysis using gene prediction method: Protein Homology*	*Metal ABC transporter permease; Derived by automated computational analysis using gene prediction method: Protein Homology*	0.987
sitD	sitB	CY43_14960	CY43_14950	*Metal ABC transporter permease; Derived by automated computational analysis using gene prediction method: Protein Homology*	*Manganese/iron transporter ATP-binding protein; With SitACD is involved in the transport of manganese and iron; Derived by automated computational analysis using gene prediction method: Protein Homology*	0.999
sitD	sitA	CY43_14960	CY43_14945	*Metal ABC transporter permease; Derived by automated computational analysis using gene prediction method: Protein Homology*	*Metal ABC transporter substrate-binding protein; Derived by automated computational analysis using gene prediction method: Protein Homology; Belongs to the bacterial solute-binding protein 9 family*	0.999
sitC	sprB	CY43_14955	CY43_14970	*Metal ABC transporter permease; Derived by automated computational analysis using gene prediction method: Protein Homology*	*Transcriptional regulator SprB; Activates Salmonella pathogenicity island 4 genes and weakly represses Salmonella pathogenicity island 1 genes; Derived by automated computational analysis using gene prediction method: Protein Homology*	0.559
sitC	sitD	CY43_14955	CY43_14960	*Metal ABC transporter permease; Derived by automated computational analysis using gene prediction method: Protein Homology*	*Metal ABC transporter permease; Derived by automated computational analysis using gene prediction method: Protein Homology*	0.987
sitC	sitB	CY43_14955	CY43_14950	*Metal ABC transporter permease; Derived by automated computational analysis using gene prediction method: Protein Homology*	*Manganese/iron transporter ATP-binding protein; With SitACD is involved in the transport of manganese and iron; Derived by automated computational analysis using gene prediction method: Protein Homology*	0.999

sitC	sitA	CY43_14955	CY43_14945	*Metal ABC transporter permease; Derived by automated computational analysis using gene prediction method: Protein Homology*	*Metal ABC transporter substrate-binding protein; Derived by automated computational analysis using gene prediction method: Protein Homology; Belongs to the bacterial solute-binding protein 9 family*	0.999
sitC	sipB	CY43_14955	CY43_15065	*Metal ABC transporter permease; Derived by automated computational analysis using gene prediction method: Protein Homology*	*SPI-1 type III secretion system needle tip complex protein SipB; Cell invasion protein B; required for entry of the bacteria into the host cell; induces apoptosis in macrophages; Derived by automated computational analysis using gene prediction method: Protein Homology*	0.596
sitC	prgH	CY43_14955	CY43_15010	*Metal ABC transporter permease; Derived by automated computational analysis using gene prediction method: Protein Homology*	*Type III secretion system inner membrane ring protein PrgH; With InvG, PrgI, and Prg K makes up the membrane spanning needle complex; Derived by automated computational analysis using gene prediction method: Protein Homology*	0.658
sitB	sitD	CY43_14950	CY43_14960	*Manganese/iron transporter ATP-binding protein; With SitACD is involved in the transport of manganese and iron; Derived by automated computational analysis using gene prediction method: Protein Homology*	*Metal ABC transporter permease; Derived by automated computational analysis using gene prediction method: Protein Homology*	0.999
sitB	sitC	CY43_14950	CY43_14955	*Manganese/iron transporter ATP-binding protein; With SitACD is involved in the transport of manganese and iron; Derived by automated computational analysis using gene prediction method: Protein Homology*	*Metal ABC transporter permease; Derived by automated computational analysis using gene prediction method: Protein Homology*	0.999
sitB	sitA	CY43_14950	CY43_14945	*Manganese/iron transporter ATP-binding protein; With SitACD is involved in the transport of manganese and iron; Derived by automated computational analysis using gene prediction method: Protein Homology*	*Metal ABC transporter substrate-binding protein; Derived by automated computational analysis using gene prediction method: Protein Homology; Belongs to the bacterial solute-binding protein 9 family*	0.999
sitA	sitD	CY43_14945	CY43_14960	*Metal ABC transporter substrate-binding protein; Derived by automated computational analysis using gene prediction method: Protein Homology; Belongs to the bacterial solute-binding protein 9 family*	*Metal ABC transporter permease; Derived by automated computational analysis using gene prediction method: Protein Homology*	0.999
sitA	sitC	CY43_14945	CY43_14955	*Metal ABC transporter substrate-binding protein; Derived by automated computational analysis using gene prediction method: Protein*	*Metal ABC transporter permease; Derived by automated computational analysis using gene prediction method: Protein Homology*	0.999

				Homology; Belongs to the bacterial solute-binding protein 9 family		
sitA	sitB	CY43_14945	CY43_14950	*Metal ABC transporter substrate-binding protein; Derived by automated computational analysis using gene prediction method: Protein Homology; Belongs to the bacterial solute-binding protein 9 family*	*Manganese/iron transporter ATP-binding protein; With SitACD is involved in the transport of manganese and iron; Derived by automated computational analysis using gene prediction method: Protein Homology*	0.999
sipD	sptP	CY43_15055	CY43_15030	*Involved in the transport of effector protein by the type III secretion system of Salmonella pathogenicity island 1; Derived by automated computational analysis using gene prediction method: Protein Homology*	*SPI-1 type III secretion system effector GTPase-activating protein SptP; GTP-activating protein/tyrosine phosphatase; facilitates bacterial survival in host cells; Derived by automated computational analysis using gene prediction method: Protein Homology*	0.956
sipD	spaQ	CY43_15055	CY43_15085	*Involved in the transport of effector protein by the type III secretion system of Salmonella pathogenicity island 1; Derived by automated computational analysis using gene prediction method: Protein Homology*	*SPI-1 type III secretion system export apparatus protein SpaQ; Involved in the surface presentation of antigens needed for the invasion of Salmonella into host cells; Derived by automated computational analysis using gene prediction method: Protein Homology*	0.554
sipD	spaO	CY43_15055	CY43_15095	*Involved in the transport of effector protein by the type III secretion system of Salmonella pathogenicity island 1; Derived by automated computational analysis using gene prediction method: Protein Homology*	*Involved in a secretory pathway responsible for the surface presentation of determinants needed for the entry of Salmonella species into mammalian cells; Derived by automated computational analysis using gene prediction method: Protein Homology*	0.844
sipD	sipC	CY43_15055	CY43_15060	*Involved in the transport of effector protein by the type III secretion system of Salmonella pathogenicity island 1; Derived by automated computational analysis using gene prediction method: Protein Homology*	*SPI-1 type III secretion system needle tip complex protein SipC; Cell invasion protein C; required for entry of the bacteria into the host cell; binds to actin; Derived by automated computational analysis using gene prediction method: Protein Homology*	0.993
sipD	sipB	CY43_15055	CY43_15065	*Involved in the transport of effector protein by the type III secretion system of Salmonella pathogenicity island 1; Derived by automated computational analysis using gene prediction method: Protein Homology*	*SPI-1 type III secretion system needle tip complex protein SipB; Cell invasion protein B; required for entry of the bacteria into the host cell; induces apoptosis in macrophages; Derived by automated computational analysis using gene prediction method: Protein Homology*	0.994
sipD	sipA	CY43_15055	CY43_15050	*Involved in the transport of effector protein by the type III secretion system of Salmonella pathogenicity island 1; Derived by automated*	*SPI-1 type III secretion system effector SipA; Cell invasion protein A; actin-binding; required for entry of the Salmonella into the host cell; Derived by*	0.993

				computational analysis using gene prediction method: Protein Homology	*automated computational analysis using gene prediction method: Protein Homology*	
sipD	sicP	CY43_15055	CY43_15035	*Involved in the transport of effector protein by the type III secretion system of Salmonella pathogenicity island 1; Derived by automated computational analysis using gene prediction method: Protein Homology*	*Chaperone protein SicP; Type III secretion system chaperone; Derived by automated computational analysis using gene prediction method: Protein Homology*	0.888
sipD	prgK	CY43_15055	CY43_14995	*Involved in the transport of effector protein by the type III secretion system of Salmonella pathogenicity island 1; Derived by automated computational analysis using gene prediction method: Protein Homology*	*Lipoprotein; Derived by automated computational analysis using gene prediction method: Protein Homology*	0.758
sipD	prgJ	CY43_15055	CY43_15000	*Involved in the transport of effector protein by the type III secretion system of Salmonella pathogenicity island 1; Derived by automated computational analysis using gene prediction method: Protein Homology*	*Type III secretion system needle complex protein PrgJ; May be involved in capping the needle substructure; Derived by automated computational analysis using gene prediction method: Protein Homology*	0.586
sipD	prgI	CY43_15055	CY43_15005	*Involved in the transport of effector protein by the type III secretion system of Salmonella pathogenicity island 1; Derived by automated computational analysis using gene prediction method: Protein Homology*	*Type III secretion system needle complex protein PrgI; With InvG, PrgH, and Prg K makes up the membrane spanning needle complex; Derived by automated computational analysis using gene prediction method: Protein Homology*	0.953
sipD	prgH	CY43_15055	CY43_15010	*Involved in the transport of effector protein by the type III secretion system of Salmonella pathogenicity island 1; Derived by automated computational analysis using gene prediction method: Protein Homology*	*Type III secretion system inner membrane ring protein PrgH; With InvG, PrgI, and Prg K makes up the membrane spanning needle complex; Derived by automated computational analysis using gene prediction method: Protein Homology*	0.780
sipD	invG	CY43_15055	CY43_15130	*Involved in the transport of effector protein by the type III secretion system of Salmonella pathogenicity island 1; Derived by automated computational analysis using gene prediction method: Protein Homology*	*Type III secretion system outer membrane ring protein InvG; May be involved in the export or assemby of proteins involved in the entry of Salmonella into host cells; Derived by automated computational analysis using gene prediction method: Protein Homology*	0.823
sipD	iagB	CY43_15055	CY43_15025	*Involved in the transport of effector protein by the type III secretion system of Salmonella pathogenicity island 1; Derived by automated computational analysis using gene prediction method: Protein Homology*	*SPI-1 type III secretion system invasion protein IagB; Derived by automated computational analysis using gene prediction method: Protein Homology*	0.587

sipC	sptP	CY43_15060	CY43_15030	*SPI-1 type III secretion system needle tip complex protein SipC; Cell invasion protein C; required for entry of the bacteria into the host cell; binds to actin; Derived by automated computational analysis using gene prediction method: Protein Homology*	*SPI-1 type III secretion system effector GTPase-activating protein SptP; GTP-activating protein/tyrosine phosphatase; facilitates bacterial survival in host cells; Derived by automated computational analysis using gene prediction method: Protein Homology*	0.980
sipC	spaQ	CY43_15060	CY43_15085	*SPI-1 type III secretion system needle tip complex protein SipC; Cell invasion protein C; required for entry of the bacteria into the host cell; binds to actin; Derived by automated computational analysis using gene prediction method: Protein Homology*	*SPI-1 type III secretion system export apparatus protein SpaQ; Involved in the surface presentation of antigens needed for the invasion of Salmonella into host cells; Derived by automated computational analysis using gene prediction method: Protein Homology*	0.574
sipC	spaO	CY43_15060	CY43_15095	*SPI-1 type III secretion system needle tip complex protein SipC; Cell invasion protein C; required for entry of the bacteria into the host cell; binds to actin; Derived by automated computational analysis using gene prediction method: Protein Homology*	*Involved in a secretory pathway responsible for the surface presentation of determinants needed for the entry of Salmonella species into mammalian cells; Derived by automated computational analysis using gene prediction method: Protein Homology*	0.860
sipC	sipD	CY43_15060	CY43_15055	*SPI-1 type III secretion system needle tip complex protein SipC; Cell invasion protein C; required for entry of the bacteria into the host cell; binds to actin; Derived by automated computational analysis using gene prediction method: Protein Homology*	*Involved in the transport of effector protein by the type III secretion system of Salmonella pathogenicity island 1; Derived by automated computational analysis using gene prediction method: Protein Homology*	0.993
sipC	sipB	CY43_15060	CY43_15065	*SPI-1 type III secretion system needle tip complex protein SipC; Cell invasion protein C; required for entry of the bacteria into the host cell; binds to actin; Derived by automated computational analysis using gene prediction method: Protein Homology*	*SPI-1 type III secretion system needle tip complex protein SipB; Cell invasion protein B; required for entry of the bacteria into the host cell; induces apoptosis in macrophages; Derived by automated computational analysis using gene prediction method: Protein Homology*	0.997
sipC	sipA	CY43_15060	CY43_15050	*SPI-1 type III secretion system needle tip complex protein SipC; Cell invasion protein C; required for entry of the bacteria into the host cell; binds to actin; Derived by automated computational analysis using gene prediction method: Protein Homology*	*SPI-1 type III secretion system effector SipA; Cell invasion protein A; actin-binding; required for entry of the Salmonella into the host cell; Derived by automated computational analysis using gene prediction method: Protein Homology*	0.992
sipC	sicP	CY43_15060	CY43_15035	*SPI-1 type III secretion system needle tip complex protein SipC; Cell invasion protein C; required*	*Chaperone protein SicP; Type III secretion system chaperone; Derived by automated computational*	0.823

				for entry of the bacteria into the host cell; binds to actin; Derived by automated computational analysis using gene prediction method: Protein Homology	*analysis using gene prediction method: Protein Homology*	
sipC	prgK	CY43_15060	CY43_14995	*SPI-1 type III secretion system needle tip complex protein SipC; Cell invasion protein C; required for entry of the bacteria into the host cell; binds to actin; Derived by automated computational analysis using gene prediction method: Protein Homology*	*Lipoprotein; Derived by automated computational analysis using gene prediction method: Protein Homology*	0.728
sipC	prgJ	CY43_15060	CY43_15000	*SPI-1 type III secretion system needle tip complex protein SipC; Cell invasion protein C; required for entry of the bacteria into the host cell; binds to actin; Derived by automated computational analysis using gene prediction method: Protein Homology*	*Type III secretion system needle complex protein PrgJ; May be involved in capping the needle substructure; Derived by automated computational analysis using gene prediction method: Protein Homology*	0.576
sipC	prgI	CY43_15060	CY43_15005	*SPI-1 type III secretion system needle tip complex protein SipC; Cell invasion protein C; required for entry of the bacteria into the host cell; binds to actin; Derived by automated computational analysis using gene prediction method: Protein Homology*	*Type III secretion system needle complex protein PrgI; With InvG, PrgH, and Prg K makes up the membrane spanning needle complex; Derived by automated computational analysis using gene prediction method: Protein Homology*	0.849
sipC	prgH	CY43_15060	CY43_15010	*SPI-1 type III secretion system needle tip complex protein SipC; Cell invasion protein C; required for entry of the bacteria into the host cell; binds to actin; Derived by automated computational analysis using gene prediction method: Protein Homology*	*Type III secretion system inner membrane ring protein PrgH; With InvG, PrgI, and Prg K makes up the membrane spanning needle complex; Derived by automated computational analysis using gene prediction method: Protein Homology*	0.860
sipC	invG	CY43_15060	CY43_15130	*SPI-1 type III secretion system needle tip complex protein SipC; Cell invasion protein C; required for entry of the bacteria into the host cell; binds to actin; Derived by automated computational analysis using gene prediction method: Protein Homology*	*Type III secretion system outer membrane ring protein InvG; May be involved in the export or assemby of proteins involved in the entry of Salmonella into host cells; Derived by automated computational analysis using gene prediction method: Protein Homology*	0.842
sipC	DD95_ 24045	CY43_15060	CY43_14490	*SPI-1 type III secretion system needle tip complex protein SipC; Cell invasion protein C; required for entry of the bacteria into the host cell; binds to actin; Derived by automated computational*	*Flagellin FliC; Structural flagella protein; individual Salmonella serotypes usually alternate between the production of 2 antigenic forms of flagella, termed phase 1 and phase 2, each specified*	0.559

				analysis using gene prediction method: Protein Homology	*by separate structural genes; Derived by automated computational analysis using gene prediction method: Protein Homology*	
sipC	DD95_ 19955	CY43_15060	CY43_10320	*SPI-1 type III secretion system needle tip complex protein SipC; Cell invasion protein C; required for entry of the bacteria into the host cell; binds to actin; Derived by automated computational analysis using gene prediction method: Protein Homology*	*Flagellin; Structural flagella protein; individual Salmonella serotypes usually alternate between the production of 2 antigenic forms of flagella, termed phase 1 and phase 2, each specified by separate structural genes; Derived by automated computational analysis using gene prediction method: Protein Homology*	0.558
sipB	sptP	CY43_15065	CY43_15030	*SPI-1 type III secretion system needle tip complex protein SipB; Cell invasion protein B; required for entry of the bacteria into the host cell; induces apoptosis in macrophages; Derived by automated computational analysis using gene prediction method: Protein Homology*	*SPI-1 type III secretion system effector GTPase-activating protein SptP; GTP-activating protein/tyrosine phosphatase; facilitates bacterial survival in host cells; Derived by automated computational analysis using gene prediction method: Protein Homology*	0.987
sipB	spaQ	CY43_15065	CY43_15085	*SPI-1 type III secretion system needle tip complex protein SipB; Cell invasion protein B; required for entry of the bacteria into the host cell; induces apoptosis in macrophages; Derived by automated computational analysis using gene prediction method: Protein Homology*	*SPI-1 type III secretion system export apparatus protein SpaQ; Involved in the surface presentation of antigens needed for the invasion of Salmonella into host cells; Derived by automated computational analysis using gene prediction method: Protein Homology*	0.891
sipB	spaO	CY43_15065	CY43_15095	*SPI-1 type III secretion system needle tip complex protein SipB; Cell invasion protein B; required for entry of the bacteria into the host cell; induces apoptosis in macrophages; Derived by automated computational analysis using gene prediction method: Protein Homology*	*Involved in a secretory pathway responsible for the surface presentation of determinants needed for the entry of Salmonella species into mammalian cells; Derived by automated computational analysis using gene prediction method: Protein Homology*	0.982
sipB	sitC	CY43_15065	CY43_14955	*SPI-1 type III secretion system needle tip complex protein SipB; Cell invasion protein B; required for entry of the bacteria into the host cell; induces apoptosis in macrophages; Derived by automated computational analysis using gene prediction method: Protein Homology*	*Metal ABC transporter permease; Derived by automated computational analysis using gene prediction method: Protein Homology*	0.596
sipB	sipD	CY43_15065	CY43_15055	*SPI-1 type III secretion system needle tip complex protein SipB; Cell invasion protein B; required for entry of the bacteria into the host cell; induces apoptosis in macrophages; Derived by automated*	*Involved in the transport of effector protein by the type III secretion system of Salmonella pathogenicity island 1; Derived by automated computational*	0.994

				computational analysis using gene prediction method: Protein Homology	*analysis using gene prediction method: Protein Homology*	
sipB	sipC	CY43_15065	CY43_15060	*SPI-1 type III secretion system needle tip complex protein SipB; Cell invasion protein B; required for entry of the bacteria into the host cell; induces apoptosis in macrophages; Derived by automated computational analysis using gene prediction method: Protein Homology*	*SPI-1 type III secretion system needle tip complex protein SipC; Cell invasion protein C; required for entry of the bacteria into the host cell; binds to actin; Derived by automated computational analysis using gene prediction method: Protein Homology*	0.997
sipB	sipA	CY43_15065	CY43_15050	*SPI-1 type III secretion system needle tip complex protein SipB; Cell invasion protein B; required for entry of the bacteria into the host cell; induces apoptosis in macrophages; Derived by automated computational analysis using gene prediction method: Protein Homology*	*SPI-1 type III secretion system effector SipA; Cell invasion protein A; actin-binding; required for entry of the Salmonella into the host cell; Derived by automated computational analysis using gene prediction method: Protein Homology*	0.991
sipB	sicP	CY43_15065	CY43_15035	*SPI-1 type III secretion system needle tip complex protein SipB; Cell invasion protein B; required for entry of the bacteria into the host cell; induces apoptosis in macrophages; Derived by automated computational analysis using gene prediction method: Protein Homology*	*Chaperone protein SicP; Type III secretion system chaperone; Derived by automated computational analysis using gene prediction method: Protein Homology*	0.828
sipB	prgK	CY43_15065	CY43_14995	*SPI-1 type III secretion system needle tip complex protein SipB; Cell invasion protein B; required for entry of the bacteria into the host cell; induces apoptosis in macrophages; Derived by automated computational analysis using gene prediction method: Protein Homology*	*Lipoprotein; Derived by automated computational analysis using gene prediction method: Protein Homology*	0.951
sipB	prgJ	CY43_15065	CY43_15000	*SPI-1 type III secretion system needle tip complex protein SipB; Cell invasion protein B; required for entry of the bacteria into the host cell; induces apoptosis in macrophages; Derived by automated computational analysis using gene prediction method: Protein Homology*	*Type III secretion system needle complex protein PrgJ; May be involved in capping the needle substructure; Derived by automated computational analysis using gene prediction method: Protein Homology*	0.847
sipB	prgI	CY43_15065	CY43_15005	*SPI-1 type III secretion system needle tip complex protein SipB; Cell invasion protein B; required for entry of the bacteria into the host cell; induces apoptosis in macrophages; Derived by automated computational analysis using gene prediction method: Protein Homology*	*Type III secretion system needle complex protein PrgI; With InvG, PrgH, and Prg K makes up the membrane spanning needle complex; Derived by automated computational analysis using gene prediction method: Protein Homology*	0.852

sipB	prgH	CY43_15065	CY43_15010	*SPI-1 type III secretion system needle tip complex protein SipB; Cell invasion protein B; required for entry of the bacteria into the host cell; induces apoptosis in macrophages; Derived by automated computational analysis using gene prediction method: Protein Homology*	*Type III secretion system inner membrane ring protein PrgH; With InvG, PrgI, and Prg K makes up the membrane spanning needle complex; Derived by automated computational analysis using gene prediction method: Protein Homology*	0.969
sipB	invG	CY43_15065	CY43_15130	*SPI-1 type III secretion system needle tip complex protein SipB; Cell invasion protein B; required for entry of the bacteria into the host cell; induces apoptosis in macrophages; Derived by automated computational analysis using gene prediction method: Protein Homology*	*Type III secretion system outer membrane ring protein InvG; May be involved in the export or assemby of proteins involved in the entry of Salmonella into host cells; Derived by automated computational analysis using gene prediction method: Protein Homology*	0.971
sipB	iagB	CY43_15065	CY43_15025	*SPI-1 type III secretion system needle tip complex protein SipB; Cell invasion protein B; required for entry of the bacteria into the host cell; induces apoptosis in macrophages; Derived by automated computational analysis using gene prediction method: Protein Homology*	*SPI-1 type III secretion system invasion protein IagB; Derived by automated computational analysis using gene prediction method: Protein Homology*	0.718
sipB	hilA	CY43_15065	CY43_15020	*SPI-1 type III secretion system needle tip complex protein SipB; Cell invasion protein B; required for entry of the bacteria into the host cell; induces apoptosis in macrophages; Derived by automated computational analysis using gene prediction method: Protein Homology*	*Invasion protein regulator; IagA; HilA; activates the expression of invasion genes and activates the expression of prgHIJK which is part of the pathogenicity island 1 type III secretion system; Derived by automated computational analysis using gene prediction method: Protein Homology*	0.876
sipB	DD95_ 24045	CY43_15065	CY43_14490	*SPI-1 type III secretion system needle tip complex protein SipB; Cell invasion protein B; required for entry of the bacteria into the host cell; induces apoptosis in macrophages; Derived by automated computational analysis using gene prediction method: Protein Homology*	*Flagellin FliC; Structural flagella protein; individual Salmonella serotypes usually alternate between the production of 2 antigenic forms of flagella, termed phase 1 and phase 2, each specified by separate structural genes; Derived by automated computational analysis using gene prediction method: Protein Homology*	0.663
sipB	DD95_ 19955	CY43_15065	CY43_10320	*SPI-1 type III secretion system needle tip complex protein SipB; Cell invasion protein B; required for entry of the bacteria into the host cell; induces apoptosis in macrophages; Derived by automated computational analysis using gene prediction method: Protein Homology*	*Flagellin; Structural flagella protein; individual Salmonella serotypes usually alternate between the production of 2 antigenic forms of flagella, termed phase 1 and phase 2, each specified by separate structural genes; Derived by automated computational analysis using gene prediction method: Protein Homology*	0.658

sipA	sptP	CY43_15050	CY43_15030	*SPI-1 type III secretion system effector SipA; Cell invasion protein A; actin-binding; required for entry of the Salmonella into the host cell; Derived by automated computational analysis using gene prediction method: Protein Homology*	*SPI-1 type III secretion system effector GTPase-activating protein SptP; GTP-activating protein/tyrosine phosphatase; facilitates bacterial survival in host cells; Derived by automated computational analysis using gene prediction method: Protein Homology*	0.988
sipA	spaQ	CY43_15050	CY43_15085	*SPI-1 type III secretion system effector SipA; Cell invasion protein A; actin-binding; required for entry of the Salmonella into the host cell; Derived by automated computational analysis using gene prediction method: Protein Homology*	*SPI-1 type III secretion system export apparatus protein SpaQ; Involved in the surface presentation of antigens needed for the invasion of Salmonella into host cells; Derived by automated computational analysis using gene prediction method: Protein Homology*	0.433
sipA	spaO	CY43_15050	CY43_15095	*SPI-1 type III secretion system effector SipA; Cell invasion protein A; actin-binding; required for entry of the Salmonella into the host cell; Derived by automated computational analysis using gene prediction method: Protein Homology*	*Involved in a secretory pathway responsible for the surface presentation of determinants needed for the entry of Salmonella species into mammalian cells; Derived by automated computational analysis using gene prediction method: Protein Homology*	0.803
sipA	sipD	CY43_15050	CY43_15055	*SPI-1 type III secretion system effector SipA; Cell invasion protein A; actin-binding; required for entry of the Salmonella into the host cell; Derived by automated computational analysis using gene prediction method: Protein Homology*	*Involved in the transport of effector protein by the type III secretion system of Salmonella pathogenicity island 1; Derived by automated computational analysis using gene prediction method: Protein Homology*	0.993
sipA	sipC	CY43_15050	CY43_15060	*SPI-1 type III secretion system effector SipA; Cell invasion protein A; actin-binding; required for entry of the Salmonella into the host cell; Derived by automated computational analysis using gene prediction method: Protein Homology*	*SPI-1 type III secretion system needle tip complex protein SipC; Cell invasion protein C; required for entry of the bacteria into the host cell; binds to actin; Derived by automated computational analysis using gene prediction method: Protein Homology*	0.992
sipA	sipB	CY43_15050	CY43_15065	*SPI-1 type III secretion system effector SipA; Cell invasion protein A; actin-binding; required for entry of the Salmonella into the host cell; Derived by automated computational analysis using gene prediction method: Protein Homology*	*SPI-1 type III secretion system needle tip complex protein SipB; Cell invasion protein B; required for entry of the bacteria into the host cell; induces apoptosis in macrophages; Derived by automated computational analysis using gene prediction method: Protein Homology*	0.991
sipA	sicP	CY43_15050	CY43_15035	*SPI-1 type III secretion system effector SipA; Cell invasion protein A; actin-binding; required for entry of the Salmonella into the host cell; Derived by automated computational analysis using gene prediction method: Protein Homology*	*Chaperone protein SicP; Type III secretion system chaperone; Derived by automated computational analysis using gene prediction method: Protein Homology*	0.812

sipA	prgK	CY43_15050	CY43_14995	*SPI-1 type III secretion system effector SipA; Cell invasion protein A; actin-binding; required for entry of the Salmonella into the host cell; Derived by automated computational analysis using gene prediction method: Protein Homology*	*Lipoprotein; Derived by automated computational analysis using gene prediction method: Protein Homology*	0.716
sipA	prgJ	CY43_15050	CY43_15000	*SPI-1 type III secretion system effector SipA; Cell invasion protein A; actin-binding; required for entry of the Salmonella into the host cell; Derived by automated computational analysis using gene prediction method: Protein Homology*	*Type III secretion system needle complex protein PrgJ; May be involved in capping the needle substructure; Derived by automated computational analysis using gene prediction method: Protein Homology*	0.717
sipA	prgI	CY43_15050	CY43_15005	*SPI-1 type III secretion system effector SipA; Cell invasion protein A; actin-binding; required for entry of the Salmonella into the host cell; Derived by automated computational analysis using gene prediction method: Protein Homology*	*Type III secretion system needle complex protein PrgI; With InvG, PrgH, and Prg K makes up the membrane spanning needle complex; Derived by automated computational analysis using gene prediction method: Protein Homology*	0.755
sipA	prgH	CY43_15050	CY43_15010	*SPI-1 type III secretion system effector SipA; Cell invasion protein A; actin-binding; required for entry of the Salmonella into the host cell; Derived by automated computational analysis using gene prediction method: Protein Homology*	*Type III secretion system inner membrane ring protein PrgH; With InvG, PrgI, and Prg K makes up the membrane spanning needle complex; Derived by automated computational analysis using gene prediction method: Protein Homology*	0.811
sipA	invG	CY43_15050	CY43_15130	*SPI-1 type III secretion system effector SipA; Cell invasion protein A; actin-binding; required for entry of the Salmonella into the host cell; Derived by automated computational analysis using gene prediction method: Protein Homology*	*Type III secretion system outer membrane ring protein InvG; May be involved in the export or assemby of proteins involved in the entry of Salmonella into host cells; Derived by automated computational analysis using gene prediction method: Protein Homology*	0.850
sipA	iagB	CY43_15050	CY43_15025	*SPI-1 type III secretion system effector SipA; Cell invasion protein A; actin-binding; required for entry of the Salmonella into the host cell; Derived by automated computational analysis using gene prediction method: Protein Homology*	*SPI-1 type III secretion system invasion protein IagB; Derived by automated computational analysis using gene prediction method: Protein Homology*	0.407
sipA	DD95_ 24045	CY43_15050	CY43_14490	*SPI-1 type III secretion system effector SipA; Cell invasion protein A; actin-binding; required for entry of the Salmonella into the host cell; Derived by automated computational analysis using gene prediction method: Protein Homology*	*Flagellin FliC; Structural flagella protein; individual Salmonella serotypes usually alternate between the production of 2 antigenic forms of flagella, termed phase 1 and phase 2, each specified by separate structural genes; Derived by automated computational analysis using gene prediction method: Protein Homology*	0.705

sipA	DD95_19955	CY43_15050	CY43_10320	*SPI-1 type III secretion system effector SipA; Cell invasion protein A; actin-binding; required for entry of the Salmonella into the host cell; Derived by automated computational analysis using gene prediction method: Protein Homology*	*Flagellin; Structural flagella protein; individual Salmonella serotypes usually alternate between the production of 2 antigenic forms of flagella, termed phase 1 and phase 2, each specified by separate structural genes; Derived by automated computational analysis using gene prediction method: Protein Homology*	0.700
sicP	sptP	CY43_15035	CY43_15030	*Chaperone protein SicP; Type III secretion system chaperone; Derived by automated computational analysis using gene prediction method: Protein Homology*	*SPI-1 type III secretion system effector GTPase-activating protein SptP; GTP-activating protein/tyrosine phosphatase; facilitates bacterial survival in host cells; Derived by automated computational analysis using gene prediction method: Protein Homology*	0.998
sicP	spaQ	CY43_15035	CY43_15085	*Chaperone protein SicP; Type III secretion system chaperone; Derived by automated computational analysis using gene prediction method: Protein Homology*	*SPI-1 type III secretion system export apparatus protein SpaQ; Involved in the surface presentation of antigens needed for the invasion of Salmonella into host cells; Derived by automated computational analysis using gene prediction method: Protein Homology*	0.499
sicP	spaO	CY43_15035	CY43_15095	*Chaperone protein SicP; Type III secretion system chaperone; Derived by automated computational analysis using gene prediction method: Protein Homology*	*Involved in a secretory pathway responsible for the surface presentation of determinants needed for the entry of Salmonella species into mammalian cells; Derived by automated computational analysis using gene prediction method: Protein Homology*	0.752
sicP	sipD	CY43_15035	CY43_15055	*Chaperone protein SicP; Type III secretion system chaperone; Derived by automated computational analysis using gene prediction method: Protein Homology*	*Involved in the transport of effector protein by the type III secretion system of Salmonella pathogenicity island 1; Derived by automated computational analysis using gene prediction method: Protein Homology*	0.888
sicP	sipC	CY43_15035	CY43_15060	*Chaperone protein SicP; Type III secretion system chaperone; Derived by automated computational analysis using gene prediction method: Protein Homology*	*SPI-1 type III secretion system needle tip complex protein SipC; Cell invasion protein C; required for entry of the bacteria into the host cell; binds to actin; Derived by automated computational analysis using gene prediction method: Protein Homology*	0.823
sicP	sipB	CY43_15035	CY43_15065	*Chaperone protein SicP; Type III secretion system chaperone; Derived by automated computational analysis using gene prediction method: Protein Homology*	*SPI-1 type III secretion system needle tip complex protein SipB; Cell invasion protein B; required for entry of the bacteria into the host cell; induces apoptosis in macrophages; Derived by automated*	0.828

					computational analysis using gene prediction method: Protein Homology	
sicP	sipA	CY43_15035	CY43_15050	*Chaperone protein SicP; Type III secretion system chaperone; Derived by automated computational analysis using gene prediction method: Protein Homology*	*SPI-1 type III secretion system effector SipA; Cell invasion protein A; actin-binding; required for entry of the Salmonella into the host cell; Derived by automated computational analysis using gene prediction method: Protein Homology*	0.812
sicP	prgJ	CY43_15035	CY43_15000	*Chaperone protein SicP; Type III secretion system chaperone; Derived by automated computational analysis using gene prediction method: Protein Homology*	*Type III secretion system needle complex protein PrgJ; May be involved in capping the needle substructure; Derived by automated computational analysis using gene prediction method: Protein Homology*	0.682
sicP	prgI	CY43_15035	CY43_15005	*Chaperone protein SicP; Type III secretion system chaperone; Derived by automated computational analysis using gene prediction method: Protein Homology*	*Type III secretion system needle complex protein PrgI; With InvG, PrgH, and Prg K makes up the membrane spanning needle complex; Derived by automated computational analysis using gene prediction method: Protein Homology*	0.521
sicP	prgH	CY43_15035	CY43_15010	*Chaperone protein SicP; Type III secretion system chaperone; Derived by automated computational analysis using gene prediction method: Protein Homology*	*Type III secretion system inner membrane ring protein PrgH; With InvG, PrgI, and Prg K makes up the membrane spanning needle complex; Derived by automated computational analysis using gene prediction method: Protein Homology*	0.739
sicP	iagB	CY43_15035	CY43_15025	*Chaperone protein SicP; Type III secretion system chaperone; Derived by automated computational analysis using gene prediction method: Protein Homology*	*SPI-1 type III secretion system invasion protein IagB; Derived by automated computational analysis using gene prediction method: Protein Homology*	0.607
prgK	sptP	CY43_14995	CY43_15030	*Lipoprotein; Derived by automated computational analysis using gene prediction method: Protein Homology*	*SPI-1 type III secretion system effector GTPase-activating protein SptP; GTP-activating protein/tyrosine phosphatase; facilitates bacterial survival in host cells; Derived by automated computational analysis using gene prediction method: Protein Homology*	0.750
prgK	sprB	CY43_14995	CY43_14970	*Lipoprotein; Derived by automated computational analysis using gene prediction method: Protein Homology*	*Transcriptional regulator SprB; Activates Salmonella pathogenicity island 4 genes and weakly represses Salmonella pathogenicity island 1 genes; Derived by automated computational analysis using gene prediction method: Protein Homology*	0.434

prgK	spaQ	CY43_14995	CY43_15085	*Lipoprotein; Derived by automated computational analysis using gene prediction method: Protein Homology*	*SPI-1 type III secretion system export apparatus protein SpaQ; Involved in the surface presentation of antigens needed for the invasion of Salmonella into host cells; Derived by automated computational analysis using gene prediction method: Protein Homology*	0.957
prgK	spaO	CY43_14995	CY43_15095	*Lipoprotein; Derived by automated computational analysis using gene prediction method: Protein Homology*	*Involved in a secretory pathway responsible for the surface presentation of determinants needed for the entry of Salmonella species into mammalian cells; Derived by automated computational analysis using gene prediction method: Protein Homology*	0.960
prgK	sipD	CY43_14995	CY43_15055	*Lipoprotein; Derived by automated computational analysis using gene prediction method: Protein Homology*	*Involved in the transport of effector protein by the type III secretion system of Salmonella pathogenicity island 1; Derived by automated computational analysis using gene prediction method: Protein Homology*	0.758
prgK	sipC	CY43_14995	CY43_15060	*Lipoprotein; Derived by automated computational analysis using gene prediction method: Protein Homology*	*SPI-1 type III secretion system needle tip complex protein SipC; Cell invasion protein C; required for entry of the bacteria into the host cell; binds to actin; Derived by automated computational analysis using gene prediction method: Protein Homology*	0.728
prgK	sipB	CY43_14995	CY43_15065	*Lipoprotein; Derived by automated computational analysis using gene prediction method: Protein Homology*	*SPI-1 type III secretion system needle tip complex protein SipB; Cell invasion protein B; required for entry of the bacteria into the host cell; induces apoptosis in macrophages; Derived by automated computational analysis using gene prediction method: Protein Homology*	0.951
prgK	sipA	CY43_14995	CY43_15050	*Lipoprotein; Derived by automated computational analysis using gene prediction method: Protein Homology*	*SPI-1 type III secretion system effector SipA; Cell invasion protein A; actin-binding; required for entry of the Salmonella into the host cell; Derived by automated computational analysis using gene prediction method: Protein Homology*	0.716
prgK	prgJ	CY43_14995	CY43_15000	*Lipoprotein; Derived by automated computational analysis using gene prediction method: Protein Homology*	*Type III secretion system needle complex protein PrgJ; May be involved in capping the needle substructure; Derived by automated computational analysis using gene prediction method: Protein Homology*	0.990

prgK	prgI	CY43_14995	CY43_15005	*Lipoprotein; Derived by automated computational analysis using gene prediction method: Protein Homology*	*Type III secretion system needle complex protein PrgI; With InvG, PrgH, and Prg K makes up the membrane spanning needle complex; Derived by automated computational analysis using gene prediction method: Protein Homology*	0.991
prgK	prgH	CY43_14995	CY43_15010	*Lipoprotein; Derived by automated computational analysis using gene prediction method: Protein Homology*	*Type III secretion system inner membrane ring protein PrgH; With InvG, PrgI, and Prg K makes up the membrane spanning needle complex; Derived by automated computational analysis using gene prediction method: Protein Homology*	0.999
prgK	invG	CY43_14995	CY43_15130	*Lipoprotein; Derived by automated computational analysis using gene prediction method: Protein Homology*	*Type III secretion system outer membrane ring protein InvG; May be involved in the export or assemby of proteins involved in the entry of Salmonella into host cells; Derived by automated computational analysis using gene prediction method: Protein Homology*	0.998
prgK	iagB	CY43_14995	CY43_15025	*Lipoprotein; Derived by automated computational analysis using gene prediction method: Protein Homology*	*SPI-1 type III secretion system invasion protein IagB; Derived by automated computational analysis using gene prediction method: Protein Homology*	0.858
prgK	hilA	CY43_14995	CY43_15020	*Lipoprotein; Derived by automated computational analysis using gene prediction method: Protein Homology*	*Invasion protein regulator; IagA; HilA; activates the expression of invasion genes and activates the expression of prgHIJK which is part of the pathogenicity island 1 type III secretion system; Derived by automated computational analysis using gene prediction method: Protein Homology*	0.680
prgJ	sptP	CY43_15000	CY43_15030	*Type III secretion system needle complex protein PrgJ; May be involved in capping the needle substructure; Derived by automated computational analysis using gene prediction method: Protein Homology*	*SPI-1 type III secretion system effector GTPase-activating protein SptP; GTP-activating protein/tyrosine phosphatase; facilitates bacterial survival in host cells; Derived by automated computational analysis using gene prediction method: Protein Homology*	0.460
prgJ	spaQ	CY43_15000	CY43_15085	*Type III secretion system needle complex protein PrgJ; May be involved in capping the needle substructure; Derived by automated computational analysis using gene prediction method: Protein Homology*	*SPI-1 type III secretion system export apparatus protein SpaQ; Involved in the surface presentation of antigens needed for the invasion of Salmonella into host cells; Derived by automated computational analysis using gene prediction method: Protein Homology*	0.775

prgJ	spaO	CY43_15000	CY43_15095	*Type III secretion system needle complex protein PrgJ; May be involved in capping the needle substructure; Derived by automated computational analysis using gene prediction method: Protein Homology*	*Involved in a secretory pathway responsible for the surface presentation of determinants needed for the entry of Salmonella species into mammalian cells; Derived by automated computational analysis using gene prediction method: Protein Homology*	0.668
prgJ	sipD	CY43_15000	CY43_15055	*Type III secretion system needle complex protein PrgJ; May be involved in capping the needle substructure; Derived by automated computational analysis using gene prediction method: Protein Homology*	*Involved in the transport of effector protein by the type III secretion system of Salmonella pathogenicity island 1; Derived by automated computational analysis using gene prediction method: Protein Homology*	0.586
prgJ	sipC	CY43_15000	CY43_15060	*Type III secretion system needle complex protein PrgJ; May be involved in capping the needle substructure; Derived by automated computational analysis using gene prediction method: Protein Homology*	*SPI-1 type III secretion system needle tip complex protein SipC; Cell invasion protein C; required for entry of the bacteria into the host cell; binds to actin; Derived by automated computational analysis using gene prediction method: Protein Homology*	0.576
prgJ	sipB	CY43_15000	CY43_15065	*Type III secretion system needle complex protein PrgJ; May be involved in capping the needle substructure; Derived by automated computational analysis using gene prediction method: Protein Homology*	*SPI-1 type III secretion system needle tip complex protein SipB; Cell invasion protein B; required for entry of the bacteria into the host cell; induces apoptosis in macrophages; Derived by automated computational analysis using gene prediction method: Protein Homology*	0.847
prgJ	sipA	CY43_15000	CY43_15050	*Type III secretion system needle complex protein PrgJ; May be involved in capping the needle substructure; Derived by automated computational analysis using gene prediction method: Protein Homology*	*SPI-1 type III secretion system effector SipA; Cell invasion protein A; actin-binding; required for entry of the Salmonella into the host cell; Derived by automated computational analysis using gene prediction method: Protein Homology*	0.717
prgJ	sicP	CY43_15000	CY43_15035	*Type III secretion system needle complex protein PrgJ; May be involved in capping the needle substructure; Derived by automated computational analysis using gene prediction method: Protein Homology*	*Chaperone protein SicP; Type III secretion system chaperone; Derived by automated computational analysis using gene prediction method: Protein Homology*	0.682
prgJ	prgK	CY43_15000	CY43_14995	*Type III secretion system needle complex protein PrgJ; May be involved in capping the needle substructure; Derived by automated computational analysis using gene prediction method: Protein Homology*	*Lipoprotein; Derived by automated computational analysis using gene prediction method: Protein Homology*	0.990
prgJ	prgI	CY43_15000	CY43_15005	*Type III secretion system needle complex protein PrgJ; May be involved in capping the needle*	*Type III secretion system needle complex protein PrgI; With InvG, PrgH, and Prg K makes up the*	0.996

				substructure; Derived by automated computational analysis using gene prediction method: Protein Homology	*membrane spanning needle complex; Derived by automated computational analysis using gene prediction method: Protein Homology*	
prgJ	prgH	CY43_15000	CY43_15010	*Type III secretion system needle complex protein PrgJ; May be involved in capping the needle substructure; Derived by automated computational analysis using gene prediction method: Protein Homology*	*Type III secretion system inner membrane ring protein PrgH; With InvG, PrgI, and Prg K makes up the membrane spanning needle complex; Derived by automated computational analysis using gene prediction method: Protein Homology*	0.977
prgJ	invG	CY43_15000	CY43_15130	*Type III secretion system needle complex protein PrgJ; May be involved in capping the needle substructure; Derived by automated computational analysis using gene prediction method: Protein Homology*	*Type III secretion system outer membrane ring protein InvG; May be involved in the export or assemby of proteins involved in the entry of Salmonella into host cells; Derived by automated computational analysis using gene prediction method: Protein Homology*	0.858
prgJ	DD95_ 24045	CY43_15000	CY43_14490	*Type III secretion system needle complex protein PrgJ; May be involved in capping the needle substructure; Derived by automated computational analysis using gene prediction method: Protein Homology*	*Flagellin FliC; Structural flagella protein; individual Salmonella serotypes usually alternate between the production of 2 antigenic forms of flagella, termed phase 1 and phase 2, each specified by separate structural genes; Derived by automated computational analysis using gene prediction method: Protein Homology*	0.963
prgJ	DD95_ 19955	CY43_15000	CY43_10320	*Type III secretion system needle complex protein PrgJ; May be involved in capping the needle substructure; Derived by automated computational analysis using gene prediction method: Protein Homology*	*Flagellin; Structural flagella protein; individual Salmonella serotypes usually alternate between the production of 2 antigenic forms of flagella, termed phase 1 and phase 2, each specified by separate structural genes; Derived by automated computational analysis using gene prediction method: Protein Homology*	0.963
prgI	sptP	CY43_15005	CY43_15030	*Type III secretion system needle complex protein PrgI; With InvG, PrgH, and Prg K makes up the membrane spanning needle complex; Derived by automated computational analysis using gene prediction method: Protein Homology*	*SPI-1 type III secretion system effector GTPase-activating protein SptP; GTP-activating protein/tyrosine phosphatase; facilitates bacterial survival in host cells; Derived by automated computational analysis using gene prediction method: Protein Homology*	0.757
prgI	sprB	CY43_15005	CY43_14970	*Type III secretion system needle complex protein PrgI; With InvG, PrgH, and Prg K makes up the membrane spanning needle complex; Derived by*	*Transcriptional regulator SprB; Activates Salmonella pathogenicity island 4 genes and weakly represses Salmonella pathogenicity island 1 genes;*	0.494

				automated computational analysis using gene prediction method: Protein Homology	*Derived by automated computational analysis using gene prediction method: Protein Homology*	
prgI	spaQ	CY43_15005	CY43_15085	*Type III secretion system needle complex protein PrgI; With InvG, PrgH, and Prg K makes up the membrane spanning needle complex; Derived by automated computational analysis using gene prediction method: Protein Homology*	*SPI-1 type III secretion system export apparatus protein SpaQ; Involved in the surface presentation of antigens needed for the invasion of Salmonella into host cells; Derived by automated computational analysis using gene prediction method: Protein Homology*	0.556
prgI	spaO	CY43_15005	CY43_15095	*Type III secretion system needle complex protein PrgI; With InvG, PrgH, and Prg K makes up the membrane spanning needle complex; Derived by automated computational analysis using gene prediction method: Protein Homology*	*Involved in a secretory pathway responsible for the surface presentation of determinants needed for the entry of Salmonella species into mammalian cells; Derived by automated computational analysis using gene prediction method: Protein Homology*	0.810
prgI	sipD	CY43_15005	CY43_15055	*Type III secretion system needle complex protein PrgI; With InvG, PrgH, and Prg K makes up the membrane spanning needle complex; Derived by automated computational analysis using gene prediction method: Protein Homology*	*Involved in the transport of effector protein by the type III secretion system of Salmonella pathogenicity island 1; Derived by automated computational analysis using gene prediction method: Protein Homology*	0.953
prgI	sipC	CY43_15005	CY43_15060	*Type III secretion system needle complex protein PrgI; With InvG, PrgH, and Prg K makes up the membrane spanning needle complex; Derived by automated computational analysis using gene prediction method: Protein Homology*	*SPI-1 type III secretion system needle tip complex protein SipC; Cell invasion protein C; required for entry of the bacteria into the host cell; binds to actin; Derived by automated computational analysis using gene prediction method: Protein Homology*	0.849
prgI	sipB	CY43_15005	CY43_15065	*Type III secretion system needle complex protein PrgI; With InvG, PrgH, and Prg K makes up the membrane spanning needle complex; Derived by automated computational analysis using gene prediction method: Protein Homology*	*SPI-1 type III secretion system needle tip complex protein SipB; Cell invasion protein B; required for entry of the bacteria into the host cell; induces apoptosis in macrophages; Derived by automated computational analysis using gene prediction method: Protein Homology*	0.852
prgI	sipA	CY43_15005	CY43_15050	*Type III secretion system needle complex protein PrgI; With InvG, PrgH, and Prg K makes up the membrane spanning needle complex; Derived by automated computational analysis using gene prediction method: Protein Homology*	*SPI-1 type III secretion system effector SipA; Cell invasion protein A; actin-binding; required for entry of the Salmonella into the host cell; Derived by automated computational analysis using gene prediction method: Protein Homology*	0.755
prgI	sicP	CY43_15005	CY43_15035	*Type III secretion system needle complex protein PrgI; With InvG, PrgH, and Prg K makes up the membrane spanning needle complex; Derived by*	*Chaperone protein SicP; Type III secretion system chaperone; Derived by automated computational analysis using gene prediction method: Protein Homology*	0.521

				automated computational analysis using gene prediction method: Protein Homology		
prgI	prgK	CY43_15005	CY43_14995	*Type III secretion system needle complex protein PrgI; With InvG, PrgH, and Prg K makes up the membrane spanning needle complex; Derived by automated computational analysis using gene prediction method: Protein Homology*	*Lipoprotein; Derived by automated computational analysis using gene prediction method: Protein Homology*	0.991
prgI	prgJ	CY43_15005	CY43_15000	*Type III secretion system needle complex protein PrgI; With InvG, PrgH, and Prg K makes up the membrane spanning needle complex; Derived by automated computational analysis using gene prediction method: Protein Homology*	*Type III secretion system needle complex protein PrgJ; May be involved in capping the needle substructure; Derived by automated computational analysis using gene prediction method: Protein Homology*	0.996
prgI	prgH	CY43_15005	CY43_15010	*Type III secretion system needle complex protein PrgI; With InvG, PrgH, and Prg K makes up the membrane spanning needle complex; Derived by automated computational analysis using gene prediction method: Protein Homology*	*Type III secretion system inner membrane ring protein PrgH; With InvG, PrgI, and Prg K makes up the membrane spanning needle complex; Derived by automated computational analysis using gene prediction method: Protein Homology*	0.984
prgI	invG	CY43_15005	CY43_15130	*Type III secretion system needle complex protein PrgI; With InvG, PrgH, and Prg K makes up the membrane spanning needle complex; Derived by automated computational analysis using gene prediction method: Protein Homology*	*Type III secretion system outer membrane ring protein InvG; May be involved in the export or assemby of proteins involved in the entry of Salmonella into host cells; Derived by automated computational analysis using gene prediction method: Protein Homology*	0.919
prgI	iagB	CY43_15005	CY43_15025	*Type III secretion system needle complex protein PrgI; With InvG, PrgH, and Prg K makes up the membrane spanning needle complex; Derived by automated computational analysis using gene prediction method: Protein Homology*	*SPI-1 type III secretion system invasion protein IagB; Derived by automated computational analysis using gene prediction method: Protein Homology*	0.662
prgI	DD95_24045	CY43_15005	CY43_14490	*Type III secretion system needle complex protein PrgI; With InvG, PrgH, and Prg K makes up the membrane spanning needle complex; Derived by automated computational analysis using gene prediction method: Protein Homology*	*Flagellin FliC; Structural flagella protein; individual Salmonella serotypes usually alternate between the production of 2 antigenic forms of flagella, termed phase 1 and phase 2, each specified by separate structural genes; Derived by automated computational analysis using gene prediction method: Protein Homology*	0.656
gI	DD95_19955	CY43_15005	CY43_10320	*Type III secretion system needle complex protein PrgI; With InvG, PrgH, and Prg K makes up the membrane spanning needle complex; Derived by*	*Flagellin; Structural flagella protein; individual Salmonella serotypes usually alternate between the production of 2 antigenic forms of flagella, termed*	0.596

				automated computational analysis using gene prediction method: Protein Homology	*phase 1 and phase 2, each specified by separate structural genes; Derived by automated computational analysis using gene prediction method: Protein Homology*	
prgH	sptP	CY43_15010	CY43_15030	*Type III secretion system inner membrane ring protein PrgH; With InvG, PrgI, and Prg K makes up the membrane spanning needle complex; Derived by automated computational analysis using gene prediction method: Protein Homology*	*SPI-1 type III secretion system effector GTPase-activating protein SptP; GTP-activating protein/tyrosine phosphatase; facilitates bacterial survival in host cells; Derived by automated computational analysis using gene prediction method: Protein Homology*	0.938
prgH	sprB	CY43_15010	CY43_14970	*Type III secretion system inner membrane ring protein PrgH; With InvG, PrgI, and Prg K makes up the membrane spanning needle complex; Derived by automated computational analysis using gene prediction method: Protein Homology*	*Transcriptional regulator SprB; Activates Salmonella pathogenicity island 4 genes and weakly represses Salmonella pathogenicity island 1 genes; Derived by automated computational analysis using gene prediction method: Protein Homology*	0.483
prgH	spaQ	CY43_15010	CY43_15085	*Type III secretion system inner membrane ring protein PrgH; With InvG, PrgI, and Prg K makes up the membrane spanning needle complex; Derived by automated computational analysis using gene prediction method: Protein Homology*	*SPI-1 type III secretion system export apparatus protein SpaQ; Involved in the surface presentation of antigens needed for the invasion of Salmonella into host cells; Derived by automated computational analysis using gene prediction method: Protein Homology*	0.863
prgH	spaO	CY43_15010	CY43_15095	*Type III secretion system inner membrane ring protein PrgH; With InvG, PrgI, and Prg K makes up the membrane spanning needle complex; Derived by automated computational analysis using gene prediction method: Protein Homology*	*Involved in a secretory pathway responsible for the surface presentation of determinants needed for the entry of Salmonella species into mammalian cells; Derived by automated computational analysis using gene prediction method: Protein Homology*	0.938
prgH	sitC	CY43_15010	CY43_14955	*Type III secretion system inner membrane ring protein PrgH; With InvG, PrgI, and Prg K makes up the membrane spanning needle complex; Derived by automated computational analysis using gene prediction method: Protein Homology*	*Metal ABC transporter permease; Derived by automated computational analysis using gene prediction method: Protein Homology*	0.658
prgH	sipD	CY43_15010	CY43_15055	*Type III secretion system inner membrane ring protein PrgH; With InvG, PrgI, and Prg K makes up the membrane spanning needle complex; Derived by automated computational analysis using gene prediction method: Protein Homology*	*Involved in the transport of effector protein by the type III secretion system of Salmonella pathogenicity island 1; Derived by automated computational analysis using gene prediction method: Protein Homology*	0.780
prgH	sipC	CY43_15010	CY43_15060	*Type III secretion system inner membrane ring protein PrgH; With InvG, PrgI, and Prg K makes*	*SPI-1 type III secretion system needle tip complex protein SipC; Cell invasion protein C; required for*	0.860

				up the membrane spanning needle complex; Derived by automated computational analysis using gene prediction method: Protein Homology	*entry of the bacteria into the host cell; binds to actin; Derived by automated computational analysis using gene prediction method: Protein Homology*	
prgH	sipB	CY43_15010	CY43_15065	*Type III secretion system inner membrane ring protein PrgH; With InvG, PrgI, and Prg K makes up the membrane spanning needle complex; Derived by automated computational analysis using gene prediction method: Protein Homology*	*SPI-1 type III secretion system needle tip complex protein SipB; Cell invasion protein B; required for entry of the bacteria into the host cell; induces apoptosis in macrophages; Derived by automated computational analysis using gene prediction method: Protein Homology*	0.969
prgH	sipA	CY43_15010	CY43_15050	*Type III secretion system inner membrane ring protein PrgH; With InvG, PrgI, and Prg K makes up the membrane spanning needle complex; Derived by automated computational analysis using gene prediction method: Protein Homology*	*SPI-1 type III secretion system effector SipA; Cell invasion protein A; actin-binding; required for entry of the Salmonella into the host cell; Derived by automated computational analysis using gene prediction method: Protein Homology*	0.811
prgH	sicP	CY43_15010	CY43_15035	*Type III secretion system inner membrane ring protein PrgH; With InvG, PrgI, and Prg K makes up the membrane spanning needle complex; Derived by automated computational analysis using gene prediction method: Protein Homology*	*Chaperone protein SicP; Type III secretion system chaperone; Derived by automated computational analysis using gene prediction method: Protein Homology*	0.739
prgH	prgK	CY43_15010	CY43_14995	*Type III secretion system inner membrane ring protein PrgH; With InvG, PrgI, and Prg K makes up the membrane spanning needle complex; Derived by automated computational analysis using gene prediction method: Protein Homology*	*Lipoprotein; Derived by automated computational analysis using gene prediction method: Protein Homology*	0.999
prgH	prgJ	CY43_15010	CY43_15000	*Type III secretion system inner membrane ring protein PrgH; With InvG, PrgI, and Prg K makes up the membrane spanning needle complex; Derived by automated computational analysis using gene prediction method: Protein Homology*	*Type III secretion system needle complex protein PrgJ; May be involved in capping the needle substructure; Derived by automated computational analysis using gene prediction method: Protein Homology*	0.977
prgH	prgI	CY43_15010	CY43_15005	*Type III secretion system inner membrane ring protein PrgH; With InvG, PrgI, and Prg K makes up the membrane spanning needle complex; Derived by automated computational analysis using gene prediction method: Protein Homology*	*Type III secretion system needle complex protein PrgI; With InvG, PrgH, and Prg K makes up the membrane spanning needle complex; Derived by automated computational analysis using gene prediction method: Protein Homology*	0.984
prgH	invG	CY43_15010	CY43_15130	*Type III secretion system inner membrane ring protein PrgH; With InvG, PrgI, and Prg K makes up the membrane spanning needle complex;*	*Type III secretion system outer membrane ring protein InvG; May be involved in the export or assemby of proteins involved in the entry of Salmonella into host cells; Derived by automated*	0.998

				Derived by automated computational analysis using gene prediction method: Protein Homology	*computational analysis using gene prediction method: Protein Homology*	
prgH	iagB	CY43_15010	CY43_15025	*Type III secretion system inner membrane ring protein PrgH; With InvG, PrgI, and Prg K makes up the membrane spanning needle complex; Derived by automated computational analysis using gene prediction method: Protein Homology*	*SPI-1 type III secretion system invasion protein IagB; Derived by automated computational analysis using gene prediction method: Protein Homology*	0.858
prgH	hilA	CY43_15010	CY43_15020	*Type III secretion system inner membrane ring protein PrgH; With InvG, PrgI, and Prg K makes up the membrane spanning needle complex; Derived by automated computational analysis using gene prediction method: Protein Homology*	*Invasion protein regulator; IagA; HilA; activates the expression of invasion genes and activates the expression of prgHIJK which is part of the pathogenicity island 1 type III secretion system; Derived by automated computational analysis using gene prediction method: Protein Homology*	0.816
invG	sptP	CY43_15130	CY43_15030	*Type III secretion system outer membrane ring protein InvG; May be involved in the export or assemby of proteins involved in the entry of Salmonella into host cells; Derived by automated computational analysis using gene prediction method: Protein Homology*	*SPI-1 type III secretion system effector GTPase-activating protein SptP; GTP-activating protein/tyrosine phosphatase; facilitates bacterial survival in host cells; Derived by automated computational analysis using gene prediction method: Protein Homology*	0.840
invG	spaQ	CY43_15130	CY43_15085	*Type III secretion system outer membrane ring protein InvG; May be involved in the export or assemby of proteins involved in the entry of Salmonella into host cells; Derived by automated computational analysis using gene prediction method: Protein Homology*	*SPI-1 type III secretion system export apparatus protein SpaQ; Involved in the surface presentation of antigens needed for the invasion of Salmonella into host cells; Derived by automated computational analysis using gene prediction method: Protein Homology*	0.985
invG	spaO	CY43_15130	CY43_15095	*Type III secretion system outer membrane ring protein InvG; May be involved in the export or assemby of proteins involved in the entry of Salmonella into host cells; Derived by automated computational analysis using gene prediction method: Protein Homology*	*Involved in a secretory pathway responsible for the surface presentation of determinants needed for the entry of Salmonella species into mammalian cells; Derived by automated computational analysis using gene prediction method: Protein Homology*	0.974
invG	sipD	CY43_15130	CY43_15055	*Type III secretion system outer membrane ring protein InvG; May be involved in the export or assemby of proteins involved in the entry of Salmonella into host cells; Derived by automated computational analysis using gene prediction method: Protein Homology*	*Involved in the transport of effector protein by the type III secretion system of Salmonella pathogenicity island 1; Derived by automated computational analysis using gene prediction method: Protein Homology*	0.823

invG	sipC	CY43_15130	CY43_15060	*Type III secretion system outer membrane ring protein InvG; May be involved in the export or assemby of proteins involved in the entry of Salmonella into host cells; Derived by automated computational analysis using gene prediction method: Protein Homology*	*SPI-1 type III secretion system needle tip complex protein SipC; Cell invasion protein C; required for entry of the bacteria into the host cell; binds to actin; Derived by automated computational analysis using gene prediction method: Protein Homology*	0.842
invG	sipB	CY43_15130	CY43_15065	*Type III secretion system outer membrane ring protein InvG; May be involved in the export or assemby of proteins involved in the entry of Salmonella into host cells; Derived by automated computational analysis using gene prediction method: Protein Homology*	*SPI-1 type III secretion system needle tip complex protein SipB; Cell invasion protein B; required for entry of the bacteria into the host cell; induces apoptosis in macrophages; Derived by automated computational analysis using gene prediction method: Protein Homology*	0.971
invG	sipA	CY43_15130	CY43_15050	*Type III secretion system outer membrane ring protein InvG; May be involved in the export or assemby of proteins involved in the entry of Salmonella into host cells; Derived by automated computational analysis using gene prediction method: Protein Homology*	*SPI-1 type III secretion system effector SipA; Cell invasion protein A; actin-binding; required for entry of the Salmonella into the host cell; Derived by automated computational analysis using gene prediction method: Protein Homology*	0.850
invG	prgK	CY43_15130	CY43_14995	*Type III secretion system outer membrane ring protein InvG; May be involved in the export or assemby of proteins involved in the entry of Salmonella into host cells; Derived by automated computational analysis using gene prediction method: Protein Homology*	*Lipoprotein; Derived by automated computational analysis using gene prediction method: Protein Homology*	0.998
invG	prgJ	CY43_15130	CY43_15000	*Type III secretion system outer membrane ring protein InvG; May be involved in the export or assemby of proteins involved in the entry of Salmonella into host cells; Derived by automated computational analysis using gene prediction method: Protein Homology*	*Type III secretion system needle complex protein PrgJ; May be involved in capping the needle substructure; Derived by automated computational analysis using gene prediction method: Protein Homology*	0.858
invG	prgI	CY43_15130	CY43_15005	*Type III secretion system outer membrane ring protein InvG; May be involved in the export or assemby of proteins involved in the entry of Salmonella into host cells; Derived by automated computational analysis using gene prediction method: Protein Homology*	*Type III secretion system needle complex protein PrgI; With InvG, PrgH, and Prg K makes up the membrane spanning needle complex; Derived by automated computational analysis using gene prediction method: Protein Homology*	0.919
invG	prgH	CY43_15130	CY43_15010	*Type III secretion system outer membrane ring protein InvG; May be involved in the export or*	*Type III secretion system inner membrane ring protein PrgH; With InvG, PrgI, and Prg K makes up*	0.998

				assemby of proteins involved in the entry of Salmonella into host cells; Derived by automated computational analysis using gene prediction method: Protein Homology	*the membrane spanning needle complex; Derived by automated computational analysis using gene prediction method: Protein Homology*	
invG	iagB	CY43_15130	CY43_15025	*Type III secretion system outer membrane ring protein InvG; May be involved in the export or assemby of proteins involved in the entry of Salmonella into host cells; Derived by automated computational analysis using gene prediction method: Protein Homology*	*SPI-1 type III secretion system invasion protein IagB; Derived by automated computational analysis using gene prediction method: Protein Homology*	0.882
invG	hilA	CY43_15130	CY43_15020	*Type III secretion system outer membrane ring protein InvG; May be involved in the export or assemby of proteins involved in the entry of Salmonella into host cells; Derived by automated computational analysis using gene prediction method: Protein Homology*	*Invasion protein regulator; IagA; HilA; activates the expression of invasion genes and activates the expression of prgHIJK which is part of the pathogenicity island 1 type III secretion system; Derived by automated computational analysis using gene prediction method: Protein Homology*	0.701
iagB	sptP	CY43_15025	CY43_15030	*SPI-1 type III secretion system invasion protein IagB; Derived by automated computational analysis using gene prediction method: Protein Homology*	*SPI-1 type III secretion system effector GTPase-activating protein SptP; GTP-activating protein/tyrosine phosphatase; facilitates bacterial survival in host cells; Derived by automated computational analysis using gene prediction method: Protein Homology*	0.495
iagB	sprB	CY43_15025	CY43_14970	*SPI-1 type III secretion system invasion protein IagB; Derived by automated computational analysis using gene prediction method: Protein Homology*	*Transcriptional regulator SprB; Activates Salmonella pathogenicity island 4 genes and weakly represses Salmonella pathogenicity island 1 genes; Derived by automated computational analysis using gene prediction method: Protein Homology*	0.639
iagB	spaQ	CY43_15025	CY43_15085	*SPI-1 type III secretion system invasion protein IagB; Derived by automated computational analysis using gene prediction method: Protein Homology*	*SPI-1 type III secretion system export apparatus protein SpaQ; Involved in the surface presentation of antigens needed for the invasion of Salmonella into host cells; Derived by automated computational analysis using gene prediction method: Protein Homology*	0.650
iagB	spaO	CY43_15025	CY43_15095	*SPI-1 type III secretion system invasion protein IagB; Derived by automated computational analysis using gene prediction method: Protein Homology*	*Involved in a secretory pathway responsible for the surface presentation of determinants needed for the entry of Salmonella species into mammalian cells; Derived by automated computational analysis using gene prediction method: Protein Homology*	0.747

iagB	sipD	CY43_15025	CY43_15055	*SPI-1 type III secretion system invasion protein IagB; Derived by automated computational analysis using gene prediction method: Protein Homology*	*Involved in the transport of effector protein by the type III secretion system of Salmonella pathogenicity island 1; Derived by automated computational analysis using gene prediction method: Protein Homology*	0.587
iagB	sipB	CY43_15025	CY43_15065	*SPI-1 type III secretion system invasion protein IagB; Derived by automated computational analysis using gene prediction method: Protein Homology*	*SPI-1 type III secretion system needle tip complex protein SipB; Cell invasion protein B; required for entry of the bacteria into the host cell; induces apoptosis in macrophages; Derived by automated computational analysis using gene prediction method: Protein Homology*	0.718
iagB	sipA	CY43_15025	CY43_15050	*SPI-1 type III secretion system invasion protein IagB; Derived by automated computational analysis using gene prediction method: Protein Homology*	*SPI-1 type III secretion system effector SipA; Cell invasion protein A; actin-binding; required for entry of the Salmonella into the host cell; Derived by automated computational analysis using gene prediction method: Protein Homology*	0.407
iagB	sicP	CY43_15025	CY43_15035	*SPI-1 type III secretion system invasion protein IagB; Derived by automated computational analysis using gene prediction method: Protein Homology*	*Chaperone protein SicP; Type III secretion system chaperone; Derived by automated computational analysis using gene prediction method: Protein Homology*	0.607
iagB	prgK	CY43_15025	CY43_14995	*SPI-1 type III secretion system invasion protein IagB; Derived by automated computational analysis using gene prediction method: Protein Homology*	*Lipoprotein; Derived by automated computational analysis using gene prediction method: Protein Homology*	0.858
iagB	prgI	CY43_15025	CY43_15005	*SPI-1 type III secretion system invasion protein IagB; Derived by automated computational analysis using gene prediction method: Protein Homology*	*Type III secretion system needle complex protein PrgI; With InvG, PrgH, and Prg K makes up the membrane spanning needle complex; Derived by automated computational analysis using gene prediction method: Protein Homology*	0.662
iagB	prgH	CY43_15025	CY43_15010	*SPI-1 type III secretion system invasion protein IagB; Derived by automated computational analysis using gene prediction method: Protein Homology*	*Type III secretion system inner membrane ring protein PrgH; With InvG, PrgI, and Prg K makes up the membrane spanning needle complex; Derived by automated computational analysis using gene prediction method: Protein Homology*	0.858
iagB	invG	CY43_15025	CY43_15130	*SPI-1 type III secretion system invasion protein IagB; Derived by automated computational analysis using gene prediction method: Protein Homology*	*Type III secretion system outer membrane ring protein InvG; May be involved in the export or assemby of proteins involved in the entry of Salmonella into host cells; Derived by automated*	0.882

					computational analysis using gene prediction method: Protein Homology	
iagB	hilA	CY43_15025	CY43_15020	*SPI-1 type III secretion system invasion protein IagB; Derived by automated computational analysis using gene prediction method: Protein Homology*	*Invasion protein regulator; IagA; HilA; activates the expression of invasion genes and activates the expression of prgHIJK which is part of the pathogenicity island 1 type III secretion system; Derived by automated computational analysis using gene prediction method: Protein Homology*	0.915
hilA	sptP	CY43_15020	CY43_15030	*Invasion protein regulator; IagA; HilA; activates the expression of invasion genes and activates the expression of prgHIJK which is part of the pathogenicity island 1 type III secretion system; Derived by automated computational analysis using gene prediction method: Protein Homology*	*SPI-1 type III secretion system effector GTPase-activating protein SptP; GTP-activating protein/tyrosine phosphatase; facilitates bacterial survival in host cells; Derived by automated computational analysis using gene prediction method: Protein Homology*	0.556
hilA	spaO	CY43_15020	CY43_15095	*Invasion protein regulator; IagA; HilA; activates the expression of invasion genes and activates the expression of prgHIJK which is part of the pathogenicity island 1 type III secretion system; Derived by automated computational analysis using gene prediction method: Protein Homology*	*Involved in a secretory pathway responsible for the surface presentation of determinants needed for the entry of Salmonella species into mammalian cells; Derived by automated computational analysis using gene prediction method: Protein Homology*	0.790
hilA	sipB	CY43_15020	CY43_15065	*Invasion protein regulator; IagA; HilA; activates the expression of invasion genes and activates the expression of prgHIJK which is part of the pathogenicity island 1 type III secretion system; Derived by automated computational analysis using gene prediction method: Protein Homology*	*SPI-1 type III secretion system needle tip complex protein SipB; Cell invasion protein B; required for entry of the bacteria into the host cell; induces apoptosis in macrophages; Derived by automated computational analysis using gene prediction method: Protein Homology*	0.876
hilA	prgK	CY43_15020	CY43_14995	*Invasion protein regulator; IagA; HilA; activates the expression of invasion genes and activates the expression of prgHIJK which is part of the pathogenicity island 1 type III secretion system; Derived by automated computational analysis using gene prediction method: Protein Homology*	*Lipoprotein; Derived by automated computational analysis using gene prediction method: Protein Homology*	0.680
hilA	prgH	CY43_15020	CY43_15010	*Invasion protein regulator; IagA; HilA; activates the expression of invasion genes and activates the expression of prgHIJK which is part of the pathogenicity island 1 type III secretion system; Derived by automated computational analysis using gene prediction method: Protein Homology*	*Type III secretion system inner membrane ring protein PrgH; With InvG, PrgI, and Prg K makes up the membrane spanning needle complex; Derived by automated computational analysis using gene prediction method: Protein Homology*	0.816

hilA	invG	CY43_15020	CY43_15130	*Invasion protein regulator; IagA; HilA; activates the expression of invasion genes and activates the expression of prgHIJK which is part of the pathogenicity island 1 type III secretion system; Derived by automated computational analysis using gene prediction method: Protein Homology*	*Type III secretion system outer membrane ring protein InvG; May be involved in the export or assemby of proteins involved in the entry of Salmonella into host cells; Derived by automated computational analysis using gene prediction method: Protein Homology*	0.701
hilA	iagB	CY43_15020	CY43_15025	*Invasion protein regulator; IagA; HilA; activates the expression of invasion genes and activates the expression of prgHIJK which is part of the pathogenicity island 1 type III secretion system; Derived by automated computational analysis using gene prediction method: Protein Homology*	*SPI-1 type III secretion system invasion protein IagB; Derived by automated computational analysis using gene prediction method: Protein Homology*	0.915
DD95_ 24045	sptP	CY43_14490	CY43_15030	*Flagellin FliC; Structural flagella protein; individual Salmonella serotypes usually alternate between the production of 2 antigenic forms of flagella, termed phase 1 and phase 2, each specified by separate structural genes; Derived by automated computational analysis using gene prediction method: Protein Homology*	*SPI-1 type III secretion system effector GTPase-activating protein SptP; GTP-activating protein/tyrosine phosphatase; facilitates bacterial survival in host cells; Derived by automated computational analysis using gene prediction method: Protein Homology*	0.557
DD95_ 24045	sipC	CY43_14490	CY43_15060	*Flagellin FliC; Structural flagella protein; individual Salmonella serotypes usually alternate between the production of 2 antigenic forms of flagella, termed phase 1 and phase 2, each specified by separate structural genes; Derived by automated computational analysis using gene prediction method: Protein Homology*	*SPI-1 type III secretion system needle tip complex protein SipC; Cell invasion protein C; required for entry of the bacteria into the host cell; binds to actin; Derived by automated computational analysis using gene prediction method: Protein Homology*	0.559
DD95_ 24045	sipB	CY43_14490	CY43_15065	*Flagellin FliC; Structural flagella protein; individual Salmonella serotypes usually alternate between the production of 2 antigenic forms of flagella, termed phase 1 and phase 2, each specified by separate structural genes; Derived by automated computational analysis using gene prediction method: Protein Homology*	*SPI-1 type III secretion system needle tip complex protein SipB; Cell invasion protein B; required for entry of the bacteria into the host cell; induces apoptosis in macrophages; Derived by automated computational analysis using gene prediction method: Protein Homology*	0.663
DD95_ 24045	sipA	CY43_14490	CY43_15050	*Flagellin FliC; Structural flagella protein; individual Salmonella serotypes usually alternate between the production of 2 antigenic forms of flagella, termed phase 1 and phase 2, each specified by separate structural genes; Derived by*	*SPI-1 type III secretion system effector SipA; Cell invasion protein A; actin-binding; required for entry of the Salmonella into the host cell; Derived by automated computational analysis using gene prediction method: Protein Homology*	0.705

				automated computational analysis using gene prediction method: Protein Homology		
DD95_24045	prgJ	CY43_14490	CY43_15000	*Flagellin FliC; Structural flagella protein; individual Salmonella serotypes usually alternate between the production of 2 antigenic forms of flagella, termed phase 1 and phase 2, each specified by separate structural genes; Derived by automated computational analysis using gene prediction method: Protein Homology*	*Type III secretion system needle complex protein PrgJ; May be involved in capping the needle substructure; Derived by automated computational analysis using gene prediction method: Protein Homology*	0.963
DD95_24045	prgI	CY43_14490	CY43_15005	*Flagellin FliC; Structural flagella protein; individual Salmonella serotypes usually alternate between the production of 2 antigenic forms of flagella, termed phase 1 and phase 2, each specified by separate structural genes; Derived by automated computational analysis using gene prediction method: Protein Homology*	*Type III secretion system needle complex protein PrgI; With InvG, PrgH, and Prg K makes up the membrane spanning needle complex; Derived by automated computational analysis using gene prediction method: Protein Homology*	0.656
DD95_24045	DD95_19955	CY43_14490	CY43_10320	*Flagellin FliC; Structural flagella protein; individual Salmonella serotypes usually alternate between the production of 2 antigenic forms of flagella, termed phase 1 and phase 2, each specified by separate structural genes; Derived by automated computational analysis using gene prediction method: Protein Homology*	*Flagellin; Structural flagella protein; individual Salmonella serotypes usually alternate between the production of 2 antigenic forms of flagella, termed phase 1 and phase 2, each specified by separate structural genes; Derived by automated computational analysis using gene prediction method: Protein Homology*	0.803
DD95_19955	sptP	CY43_10320	CY43_15030	*Flagellin; Structural flagella protein; individual Salmonella serotypes usually alternate between the production of 2 antigenic forms of flagella, termed phase 1 and phase 2, each specified by separate structural genes; Derived by automated computational analysis using gene prediction method: Protein Homology*	*SPI-1 type III secretion system effector GTPase-activating protein SptP; GTP-activating protein/tyrosine phosphatase; facilitates bacterial survival in host cells; Derived by automated computational analysis using gene prediction method: Protein Homology*	0.558
DD95_19955	sipC	CY43_10320	CY43_15060	*Flagellin; Structural flagella protein; individual Salmonella serotypes usually alternate between the production of 2 antigenic forms of flagella, termed phase 1 and phase 2, each specified by separate structural genes; Derived by automated computational analysis using gene prediction method: Protein Homology*	*SPI-1 type III secretion system needle tip complex protein SipC; Cell invasion protein C; required for entry of the bacteria into the host cell; binds to actin; Derived by automated computational analysis using gene prediction method: Protein Homology*	0.558

DD95_ 19955	sipB	CY43_10320	CY43_15065	*Flagellin; Structural flagella protein; individual Salmonella serotypes usually alternate between the production of 2 antigenic forms of flagella, termed phase 1 and phase 2, each specified by separate structural genes; Derived by automated computational analysis using gene prediction method: Protein Homology*	*SPI-1 type III secretion system needle tip complex protein SipB; Cell invasion protein B; required for entry of the bacteria into the host cell; induces apoptosis in macrophages; Derived by automated computational analysis using gene prediction method: Protein Homology*	0.658
DD95_ 19955	sipA	CY43_10320	CY43_15050	*Flagellin; Structural flagella protein; individual Salmonella serotypes usually alternate between the production of 2 antigenic forms of flagella, termed phase 1 and phase 2, each specified by separate structural genes; Derived by automated computational analysis using gene prediction method: Protein Homology*	*SPI-1 type III secretion system effector SipA; Cell invasion protein A; actin-binding; required for entry of the Salmonella into the host cell; Derived by automated computational analysis using gene prediction method: Protein Homology*	0.700
DD95_ 19955	prgJ	CY43_10320	CY43_15000	*Flagellin; Structural flagella protein; individual Salmonella serotypes usually alternate between the production of 2 antigenic forms of flagella, termed phase 1 and phase 2, each specified by separate structural genes; Derived by automated computational analysis using gene prediction method: Protein Homology*	*Type III secretion system needle complex protein PrgJ; May be involved in capping the needle substructure; Derived by automated computational analysis using gene prediction method: Protein Homology*	0.963
DD95_ 19955	prgI	CY43_10320	CY43_15005	*Flagellin; Structural flagella protein; individual Salmonella serotypes usually alternate between the production of 2 antigenic forms of flagella, termed phase 1 and phase 2, each specified by separate structural genes; Derived by automated computational analysis using gene prediction method: Protein Homology*	*Type III secretion system needle complex protein PrgI; With InvG, PrgH, and Prg K makes up the membrane spanning needle complex; Derived by automated computational analysis using gene prediction method: Protein Homology*	0.596
DD95_ 19955	DD95_ 24045	CY43_10320	CY43_14490	*Flagellin; Structural flagella protein; individual Salmonella serotypes usually alternate between the production of 2 antigenic forms of flagella, termed phase 1 and phase 2, each specified by separate structural genes; Derived by automated computational analysis using gene prediction method: Protein Homology*	*Flagellin FliC; Structural flagella protein; individual Salmonella serotypes usually alternate between the production of 2 antigenic forms of flagella, termed phase 1 and phase 2, each specified by separate structural genes; Derived by automated computational analysis using gene prediction method: Protein Homology*	0.803
