# Supplementary material for: Genome analysis and virulence gene expression profile of a multi drug resistant Salmonella enterica serovar Typhimurium ms202
Source: Gut Pathog. 2022 Jun 28;14:28. doi: 10.1186/s13099-022-00498-w (PMC9237969; doi:10.1186/s13099-022-00498-w)
Supplement: Supplementary file 9 — Additional file 9: Table S9. List of primers used for qRT-PCR [file 13099_2022_498_MOESM9_ESM.pdf]

| **Table S9. List of primers for qRT-PCR used in the study** | | |
|---|---|---|
| **Gene** | **Sequence (5' to 3')** | **References** |
| 16s rRNA Fw | TTCCAGTGTGGCTGGTCATC | (65) |
| 16s rRNA Rw | TGCCTGATGGAGGGGGATAA | (65) |
| sitA Fw | AAAGCACCATATTCCCGCCA | This study |
| sitA Rw | TTGACGATGGTTTCGGTCGT | This study |
| sitB Fw | GCATAATCTCGGCTCCGTCA | This study |
| sitB Rw | AAATAAAGGGGCGCTCGTCA | This study |
| sitC Fw | TCGCTGACGATTCTGCTGTT | This study |
| sitC Rw | CGACGAGACAGATCACCAGG | This study |
| sitD Fw | GCGTTAATGGGCGATGCAAT | This study |
| sitD Rw | CGATGCCCATCACCGTATCA | This study |
| hilA Fw | GGGCAGATGATACCCGATGG | (66) |
| hilA Rw | AAGAGAGAAGCGGGTTGGTG | (66) |
| ompF Fw | ATTTCAAACCCGCGAAAGCC | This study |
| ompF Rw | TCACGTATGGACAACGACCG | This study |
| ssaB Fw | TGCTGCAAGCAGTAGTGTCA | (65) |
| ssaB Rw | AAGGCCGAAGGTAATAGCCG | (65) |
| ssaV Fw | ATCTCACCGTTGGGTTGGTC | This study |
| ssaV Rw | TCTGCATCGATAACTCCGGC | This study |
| sopB Fw | GAAGACTACCAGGCGCACTT | (66) |
| sopB Rw | GATGGCGGCGAACCCTATAA | (66) |
